# Regioselective and Stereodivergent Synthesis of Enantiomerically Pure *Vic*-Diamines from Chiral β-Amino Alcohols with 2-Pyridyl and 6-(2,2′-Bipyridyl) Moieties †

**DOI:** 10.3390/molecules25030727

**Published:** 2020-02-07

**Authors:** Marzena Wosińska-Hrydczuk, Przemysław J. Boratyński, Jacek Skarżewski

**Affiliations:** Chair of Organic and Medicinal Chemistry, Faculty of Chemistry, Wrocław University of Science and Technology, Wyb. Wyspiańskiego 27, 50-370 Wrocław, Poland; marzena.wosinska@pwr.edu.pl (M.W.-H.); przemyslaw.boratynski@pwr.edu.pl (P.J.B.)

**Keywords:** chiral β-amino alcohols, Mitsunobu reaction, 2-(2-pyridyl)aziridines, slow inversion at aziridine nitrogen, stereodivergent synthesis of chiral 1,2-diamines

## Abstract

In this report, we describe the synthetic elaboration of the easily available enantiomerically pure β-amino alcohols. Attempted direct substitution of the hydroxyl group by azido-functionality in the Mitsunobu reaction with hydrazoic acid was inefficient or led to a diastereomeric mixture. These outcomes resulted from the participation of aziridines. Intentionally performed internal Mitsunobu reaction of β-amino alcohols gave eight chiral aziridines in 45–82% yield. The structural and configuration identity of products was confirmed by NMR data compared to the DFT calculated GIAO values. For 1,2,3-trisubstituted aziridines slow configurational inversion at the endocyclic nitrogen atom was observed by NMR at room temperature. Moreover, when aziridine was titrated with Zn(OAc)_2_ under NMR control, only one of two N-epimers directly participated in complexation. The aziridines underwent ring opening with HN_3_ to form the corresponding azido amines as single regio- and diastereomers in 90–97% yield. Different results were obtained for 1,2-disubstituted and 1,2,3-trisubstituted aziridines. For the later aziridines ring closure and ring opening occurred at different carbon stereocenters, thus yielding products with two inverted configurations, compared to the starting amino alcohol. The 1,2-disubstituted aziridines produced azido amines of the same configuration as the starting β-amino alcohols. To obtain a complete series of diastereomeric *vic*-diamines, we converted the amino alcohols into cyclic sulfamidates, which reacted with sodium azide in S_N_2 reaction (25–58% overall yield). The azides obtained either way underwent the Staudinger reduction, giving a series of six new chiral *vic*-diamines of defined stereochemistries.

## 1. Introduction

Enantiomerically pure β-amino alcohols belong to a privileged group of important and easily available organocatalysts and chiral building blocks [1,2]. Some of them are directly accessible as natural products (Cinchona [3] and Ephedra [4] alkaloids), the others are obtainable in a few simple synthetic steps, e.g., from natural amino acids. Recently, we have developed the synthesis of new chiral β-amino alcohols, containing metal-complexing 2-pyridyl and 6-(2,2′-bipyridyl) fragments [5]. For their further transformations into the multi-interacting ligands, we explore the exchange of the hydroxyl group into the nitrogen-containing moieties. 

**Figure 1:**
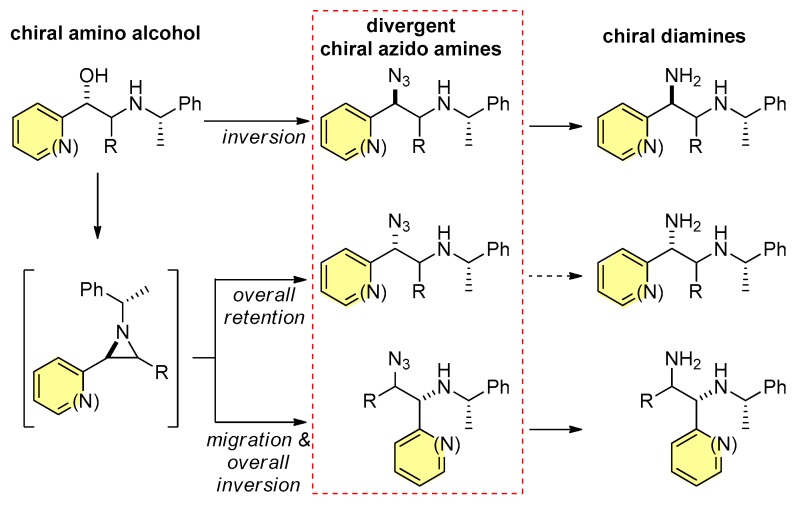
Outline of different regio- and stereochemistries observed in the conversion of amino alcohols to diamines. Only the pathway in the top row represents simple S_N_2 displacement.

The preparation of chiral vicinal diamines constitutes an important challenge and several synthetic approaches have been adopted [6]. Since chiral *vic*-diamines offer versatile transition-metal-complexing properties, we intended to develop the required procedures for the conversion of chiral amino alcohols. However, such modifications are often complicated by the participation of the neighboring amino group [7,8]. Therefore, different regio- and stereochemical outcomes can be expected [9,10]. In the present contribution different products, as outlined in Figure 1, were obtained, depending on the applied procedure and the structure of the starting amino alcohol. 

## 2. Results and Discussion

We decided to convert chiral amino alcohols into the corresponding diamines using nucleophilic displacement with an azide anion, followed by Staudinger reduction (Figure 1). In fact, our first attempts to substitute hydroxyl with azide under the Mitsunobu reaction conditions [11,12,13] suggested that aziridine intermediates could be involved. Thus, another pathway relied on the synthesis of aziridines and later subjecting them to the ring-opening reaction. A literature precedent for the regioselective ring-opening of 2-(2-pyridyl)-substituted aziridine by nitrogen, sulfur, and oxygen nucleophiles has already been published by the group of D. Savoia [14]. Finally, to enforce a direct S_N_2 pathway, we used cyclic sulfamidate intermediate products [15,16,17] that could be opened with sodium azide. 

### 2.1. Attempted Direct Substitution of the Hydroxyl Group by the External Nucleophile

**Scheme 1:**
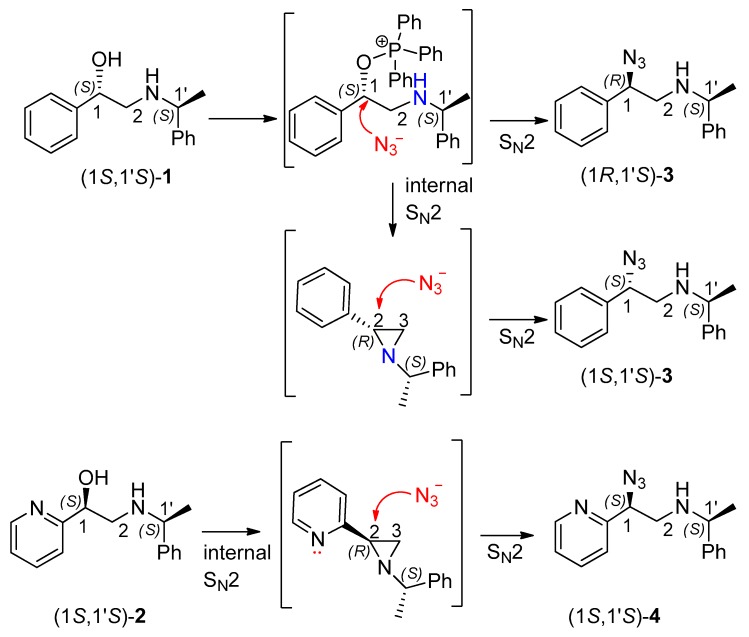
Direct synthesis of azides from amino alcohol (Ph_3_P, DEAD, HN_3_ in benzene at RT for 3 h).

To directly exchange the hydroxyl group of β-amino alcohols **1** and **2** for the azide we applied the Mitsunobu reaction conditions with hydrazoic acid [18,19]. From compound (1*S*,1′*S*)-**1** a mixture of both epimers of (1*R*,1′*S*)-**3** and (1*S*,1′*S*)-**3** in 1:1.25 ratio was obtained in 40% total yield (Scheme 1). However, from alcohol (1*S*,1′*S*)-**2** only 17% of (1*S*,1′*S*)-**4** could be isolated. Their structures were unambiguously confirmed by NMR (see Supporting Info, Figure S1). The outcomes suggested that while the product (1*R*,1′*S*)-**3** was formed via direct S_N_2 substitution of oxyphosphonium leaving group by the azide anion, the products (1*S*,1′*S*)-**3** and (1*S*,1′*S*)-**4** (overall retention) could result from ring-opening of the corresponding aziridines, as depicted in Scheme 1. Indeed, activation of the hydroxyl group resulted in internal ring-closure and formation of aziridine [20,21]. 

### 2.2. Synthesis of Aziridines

Under these circumstances, we turned to the synthesis of aziridines using the Mitsunobu reaction [22,23,24]. As has been reported for this type of process, we ran the reaction under reflux in dry toluene for 24 h [25], but a low yield of the product resulted. In an improved procedure, toluene was replaced with dry diethyl ether for the amino alcohols **1**, **2**, and **5**–**7** [5] (Method A). In the case of **2** and **5** the result was still unsatisfactory. However, applying an additional 0.5 eq of the Mitsunobu reagents (Method B) resulted in further improvement of the yield of aziridines **9** and **10** (Table 1). In an attempted exchange of the hydroxyl in **1** and **2** into the corresponding bromo-compounds using the Appel reaction [26] (2 eq of CBr_4_ and PPh_3_, then K_2_CO_3_), we again obtained aziridines **8** (a known compound [27]) and **9**. The products were the same as those obtained in the Mitsunobu reaction, so they were also formed by the direct internal substitution of the oxyphosphonium group by the secondary amine. Since only one diastereomer of the product was observed in all the cases, it is assumed that the ring closure process also occurred through S_N_2 type reaction, and inversion of the configuration should have been achieved.

### 2.3. Aziridine Structural Investigation by NMR

The ^1^H-NMR spectra of 1,2,3-trisubstituted aziridines **10** and **12** recorded at ambient temperatures demonstrated significant spectral broadening. At 283 K the NMR experiments reveal distinct sets of signals corresponding to two equilibrating species in 1:0.95 to 1:0.45 ratio, respectively (see Supporting Info, Figures S19–S28). This conclusion was further confirmed by positive-phase cross-peaks in the exchange-correlation spectroscopy experiment (Supporting Info, Figure S8). This observation can be attributed to the partially restricted inversion at the aziridine tertiary nitrogen atom [28]; thus, the observed species constituted respective *S*_N_ and *R*_N_ diastereomers. In the case of the aziridines **10** and **12**, the substituents at positions 2 and 3 are sterically similar though electronically different, and thus the chemical shifts of the corresponding nitrogen-epimers *S*_N_ vs. *R*_N_ are well separated while their quantities remain rather similar. 

The possible *N*-epimeric species were modeled and subjected to DFT geometry optimization at the B3LYP/CC-pVDZ level of theory utilizing Gaussian code [29]. For the most stable conformations, GIAO isotropic shielding values were calculated at the mPW1PW91/6-311+G(2d,p) level using the polarization continuum solvent model (PCM). The shieldings were then converted to chemical shifts using linear scaling factors [30]. These were compared with the experimental data for the very well resolved aliphatic region and showed good qualitative agreement, which made it possible to assign stereochemistry at nitrogen for both diastereomeric species (Table 2). RMS error for assignment of *S*_N_ and *R*_N_ configuration to the major and minor components, respectively, was 0.32 ppm, while the opposite assignment would produce 0.74 ppm RMS error. The energy difference between the *N*-diastereoisomers seems to be slightly overestimated by calculation (2.3 kcal/mol).

Furthermore, it is known that protonation [31] or incorporation in a metal chelate complex inhibits inversion at the nitrogen atom [32]. So far, the studied species have mostly been chelates involving aziridine nitrogen atom and heteroatoms attached to substituents at position 1 [33,34]. Several chelates involving functional groups attached to carbon atoms of the aziridine ring are also known [35,36]. In the case of compounds of type **10** such chelate would involve pyridine nitrogen as a donor atom. 

**Scheme 2:**
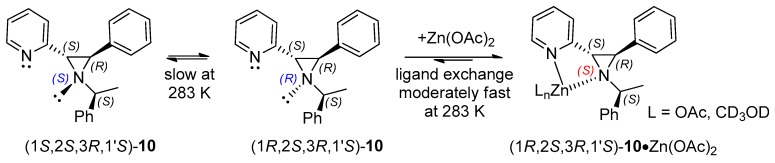
Equilibration of *N*-epimeric aziridine (2*S*,3*R,*1′*S*)-**10** in the presence of zinc acetate. Notation *S*_N_ in the complex results from CIP rules; in fact, the nitrogen configuration is the same as *R*_N_ in the free ligand.

Thus, in another experiment, a sample of (2*S*,3*R,*1′*S*)-**10** in deuterated methanol was titrated with zinc acetate and sequential ^1^H-NMR spectra were recorded. Immediately after the addition of zinc salt, one of the two sets of signals for diastereomeric species broadened significantly. The signals of the other *N*-epimer did not undergo noticeable changes in lineshape, although they linearly decreased in intensity as the quantity of zinc salt increased. With an excess of zinc acetate, one set of well-resolved signals appeared. The experiment indicated the formation of a thermodynamically sTable 1:1 zinc to ligand **10** complex (apparent equilibrium constant ca. 10^−3^ M). The complex undergoes a ligand exchange process at a moderately fast rate (approx. 10^3^ M^−1^ s^−1^ at 283 K) so coalescence is observed in the presence of excess ligand [37]. Only one *N*-epimer was shown to be directly involved in the formation of the complex. The configuration of this free ligand was assigned as *S*_N_ by means of comparison of DFT and experimental chemical shifts (*vide supra*). Here, such a configuration makes both heteroatom lone electron pairs available from one side of the aziridine ring, thus enabling chelation (Scheme 2). The complex with two acetate counterions was modeled with the help of DFT/B3LYP/CC-pVDZ level calculation, and chemical shifts were computed the same way as for the free ligand. While the general downfield change is predicted by the computed data, they display only fair similarity with the experiment, suggesting that the actual complex likely involves additional coordinated solvent molecules. Similar results were also obtained for the Zn(OAc)_2_ titration of bipyridine derivative (2*S*,3*R*,1′*S*)-**12**. There, the ligand exchange process was slower. Detailed results are shown in the Supporting Info (Figures S5–S7).

### 2.4. Synthesis of Diamines

To prepare the required diamines, we used two different routes. In the first, the azides were obtained by ring opening reaction of aziridines with HN_3._ In the second method, we obtained cyclic sulfamidates, which were transformed into the appropriate azides by the S_N_2 reaction with sodium azide. Both pathways were completed with the Staudinger reduction.

#### 2.4.1. Synthesis of Diamines by Ring Opening of Aziridines with Hydrazoic Acid

As a model reaction, we used aziridines **8** and **9**. In these cases, we observed a nucleophilic attack at the more substituted aziridine carbon (Table 3, attack **a**). From **8** we obtained azide **3** with complete regioselectivity. The pyridine-containing compound **9** gave mostly **4** (90%), but also regioisomer was identified in the crude product. In both cases, only one stereoisomer of the product was observed. A similar outcome has been reported for the reaction of pyridine-substituted aziridine with sodium azide [14]. 

**Figure 2:**
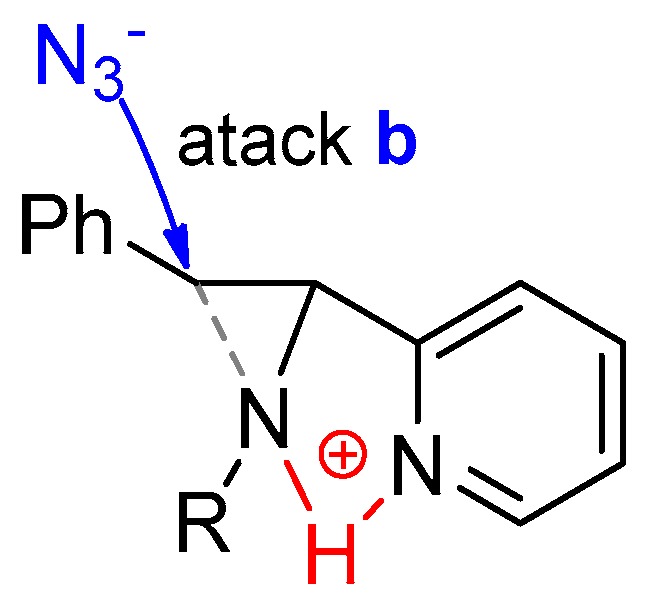
Postulated rationale for attack **b**.

In the reaction of 1,2,3-trisubstituted aziridines **10** and **12** with HN_3_, we observed a nucleophilic attack on the aziridine ring at the carbon atoms substituted with the phenyl group. In both cases this resulted in single regioisomer **13** and **14**, respectively (Table 3, attack **b**). The observed regioselectivity seems to be due to the protonation, thus activation of the phenyl-substituted carbon in the aziridine ring (Figure 2). The observed difference in the position of nucleophilic opening for analogous aziridines has already been reported [9,10,14,38].

#### 2.4.2. Synthesis of Diamines by Nucleophilic Substitution of Sulfamidates

**Scheme 3:**
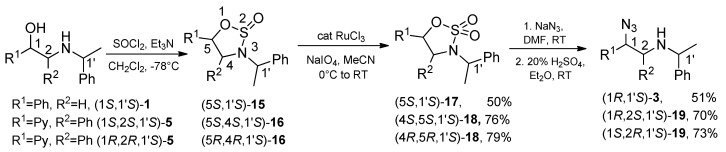
Three-step synthesis of β-azido amines from amino alcohols via sulfamidates.

To obtain different regioisomers, we used another procedure. The synthesis consisted of three steps (Scheme 3). Firstly oxathiazolidines **15** and **16** were obtained by reaction of appropriate amino alcohol **1** and **5** with thionyl chloride [39]. Then, they were oxidized to the cyclic sulfamidates **17** and **18** using sodium meta-periodate and a catalytic amount of ruthenium trichloride in acetonitrile. Next, the compounds **17** and **18** were opened [40] with sodium azide in a regio- and stereoselective reaction to give **3** and **19**. Azide **3** was obtained as the same compound as during ring opening of aziridine (2*S*,1′*S*)-**8** with HN_3_.

The structures of the azides (1*R*,1′*S*)-**3**, (1*S*,1′*S*)-**3**, (1*R*,2*R*,1′*S*)-**13**, (1S,2S,1′S)-**14**, (1*S*,2*R*,1′*S*)-**19**, and (1*R*,2*S*,1′*S*)-**19** were established based on NMR experiments. The ^1^H,^13^C HMBC correlations proved to be most informative. For all the products, the methine CH in the methylbenzylamine part correlated with the CH directly attached to the secondary amino group. For products **13** and **14**, resulting from ring opening of aziridines, this CH was found to be attached to the phenyl group while the other CH connected to the pyridine ring as seen by a ^3^*J* C-H correlations. Inverse connectivity was observed for products **19** obtained via sulfamidate (See Supporting Info, Figure S1). 

#### 2.4.3. Reduction of Azido Amines to *Vic*-Diamines

The azides **3**, **4**, **13**, **14**, and **19** were reduced to the primary amines **20**–**24** via the Staudinger [27,41] reaction using triphenylphosphine in aqueous dichloromethane in moderate to high yield (Table 4). 

## 3. Materials and Methods 

### 3.1. General

Solvents were distilled, and other reagents were used as received. Reactions were monitored by thin-layer chromatography (TLC) on silica gel 60 F-254 precoated plates, and spots were visualized with a UV lamp and/or Dragendorff reagent. Separation of products by chromatography was carried out on silica gel 60 (230–400 mesh) or Florisil (60–100 mesh). Observed rotations at 589 nm were measured using an Optical Activity Ltd. Model AA-5 automatic polarimeter (Huntington, UK). ^1^H- and ^13^C-NMR spectra (400, 600 MHz and 100, 151 MHz, respectively) were collected on Jeol 400yh and Bruker Avance II 600 instruments (Karlsruhe, Germany). The spectra were recorded in CDCl_3_ referenced to the respective residual signals of the solvent. Chemical shifts are given in parts per million (ppm) in a deuterated solvent and coupling constants (*J*) are in Hertz (Hz). High-resolution mass spectra were recorded using electrospray ionization on Waters LCT Premier XE TOF instrument (Milford, MA, USA).

### 3.2. General Procedure for the Synthesis of Aziridines

The synthesis of aziridines was performed according to a modified literature procedure [26].

**Method A**: Into the solution of the appropriate amino alcohol (1 eq, 0.5 mmol) and triphenylphosphine (1.5 eq 0.75 mmol, 197 mg) in the dry ether in an ice-water bath under an argon atmosphere, was slowly added diethyl azodicarboxylate (1.5 eq 0.75 mmol, 116 μL) via syringe. The stirring was continued at 0 °C for 15 min and then overnight at room temperature. Then the solvent was removed, and products were isolated from crude mixture by column chromatography [24].

**Method B**: The reaction was performed similarly to Method A; however, after 24 h additional 0.5 eq PPh_3_ and DEAD were added, and the reaction mixture was stirred again overnight. The product was isolated in the same way as in method A.

In the NMR we observed two *N*-epimers for aziridines (2*S*,3*R*,1′*S*)-**10**, (2*R*,3*S*,1′*S*)-**10,** (2*S*,3*R*,1′*R*)-**11**, (2*R*,3*S*,1′*S*)-**12** and (2S,3*R*,1′*S*)-**12** nitrogen atoms in different proportions.

(2*R*)-Phenyl-1-[(*S*)-1-phenylethyl]-aziridine (2*R*,1′*S*)-**8** (Method A): Colorless oil, 80 mg, 72% yield, ${\left[\alpha \right]}_{D}^{20}$ = −53 (c = 1.1, CHCl_3_), (lit. [27] ${\left[\alpha \right]}_{D}^{23}$ = −45 (c 1.5, CHCl_3_), purified by column chromatography (SiO_2_, 30% AcOEt in hexane). ^1^H-NMR (400 MHz, CDCl_3_) δ: 7.48–7.46 (m, 2H), 7.39–7.23 (m, 8H), 2.68 (q, *J* = 6.4 Hz, 1H), 2.55 (dd, *J* = 6.4, 3.4 Hz, 1H), 1.85 (d, *J* = 3.4 Hz, 1H), 1.70 (d, *J* = 6.4 Hz, 1H), 1.50 (d, *J* = 6.4 Hz, 3H). The NMR data are in agreement with the reported ones [27].

(2S)-Phenyl-1-[(*S*)-1-phenylethyl]-aziridine (2*S*,1′*S*)-**8** (Method A): White solid, 85 mg, 76% yield, m.p. 53–54 °C,$\;{\left[\alpha \right]}_{D}^{20}$ = 125 (c = 0.92, CHCl_3_), (lit. [27] ${\left[\alpha \right]}_{D}^{23}$ = 83 (c 1.3, CHCl_3_), purified by column chromatography (SiO_2_, 30% AcOEt in hexane). ^1^H-NMR (400 MHz, CDCl_3_) δ: 7.37–7.34 (m, 2H), 7.28–7.15 (m, 8H), 2.69 (q, *J* = 6.7 Hz, 1H), 2.41 (dd, *J* = 6.7, 3.4 Hz, 1H), 2.05 (d, *J* = 3.4 Hz, 1H), 1.83 (d, *J* = 6.7 Hz, 1H), 1.49 (d, *J* = 6.7 Hz, 3H). The NMR data are in agreement with the reported ones [27].

(2*R*)-Pyridin-2-yl-1-[(*S*)-1-phenylethyl]-aziridine (2*R*,1′*S*)-**9** (Method A and B): Brown oil, 57 mg, 82% yield (Method B), ${\left[\alpha \right]}_{D}^{20}$ = −63 (c = 0.38, CHCl_3_), purified by column chromatography (Florisil, 5% AcOEt in hexane). ^1^H-NMR (400 MHz, CDCl_3_) δ: 8.52 (d, *J* = 4.9 Hz, 1H), 7.62 (td, *J* = 7.6, 1.8 Hz, 1H), 7.47–7.44 (m, 2H), 7.36–7.31 (m, 3H), 7.28–7.24 (m, 1H), 7.15–7.12 (m, 1H), 2.74 (dd, *J* = 6.4, 3.0 Hz, 1H), 2.71 (d, *J* = 6.4 Hz, 1H), 1.95 (d, *J* = 3.0 Hz, 1H), 1.77 (d, *J* = 6.4 Hz, 1H), 1.47 (d, *J* = 6.4 Hz, 3H); ^13^C-NMR (101 MHz, CDCl_3_) δ: 160.2, 149.0, 144.6, 136.7, 128.4, 127.2, 127.0, 122.0, 120.3, 70.2, 43.0, 37.2, 23.6; HR-MS (ESI) [C_15_H_16_N_2_ + H]^+^ requires 225.1386; found 225.1383.

(2*S*,3*R*)-2-Pyridin-2-yl-3-phenyl-1-[(*S*)-1-phenylethyl]-aziridine (2*S*,3*R*,1′*S*)-**10** (Method A and B): We observed two epimers on nitrogen atom in ratio 1:0.5 (^1^H-NMR and ^13^C-NMR). Colorless oil, 57 mg, 76%, (Method B), ${\left[\alpha \right]}_{D}^{20}$ = 8.8 (c = 0.8, CHCl_3_), purified by column chromatography (Florisil, 5% AcOEt in hexane). ^1^H-NMR (600 MHz, 283K, CDCl_3_) major epimer δ: 8.61–8.60 (m, 1H), 7.57–7.7.56 (m, 2H), 7.42–7.39 (m, 2H), 7.32–7.01 (m, 9H) 3.97 (d, *J* = 2.9 Hz, 1H), 3.91 (q, *J* = 6.6 Hz, 1H), 3.27 (d *J* = 2.6 Hz, 1H) 1.51 (d, *J* = 6.6 Hz, 3H); minor epimer δ: 8.61–8.60 (m, 1H), 7.77–7.74 (m, 1H), 7.57–7.7.56 (m, 1H), 7.32–7.01 (m, 11H), 3.50 (dd, *J* = 19.4 2.9 Hz, 2H), 3.12 (q, *J* = 6.6 Hz, 1H), 1.45 (d, *J* = 6.6 Hz, 3H); ^13^C-NMR (151 MHz, 283K, CDCl_3_) δ:160.4, 154.8, 149.0, 148.0, 145.4, 140.8, 136.7, 135.6, 135.5, 133.4, 130.44, 130.40, 128.3, 127.7, 127.60, 127,59, 127.0, 126.9, 126.6, 126.5, 126.3, 126.2, 122.1, 121.9, 120.23, 120.18, 60.2, 58.1, 51.4, 51.0, 45.4, 45.3, 24.4, 24.2; HR-MS (ESI) [C_21_H_20_N_2_ + H]^+^ requires 301.1699; found 301.1703.

(2*R*,3*S*)-2-Pyridin-2-yl)-3-phenyl-1-[(*S*)-1-phenylethyl]-aziridine (2*R*,3*S*,1′*S*)-**10** (Method A and B): We observed two epimers on nitrogen atom in ratio ca. 1:0.95 (^1^H-NMR and ^13^C-NMR). White solid, 57 mg, 74% (Method B), m.p. 67–69 °C,$\;{\left[\alpha \right]}_{D}^{20}$ = –184.5 (c = 1.1, CHCl_3_), purified by column chromatography (Florisil, 5% AcOEt in hexane). ^1^H-NMR (600 MHz, 283K, CDCl_3_) major epimer δ: 8.71 (d, *J* = 4.0 Hz, 1H), 7.74 (td, *J* = 7.7, 1.8 Hz, 1H), 7.59–7.55 (m, 3H), 7.43–7.41 (m, 1H), 7.36–7.19 (m, 8H), 3.99 (q, *J* = 6.6 Hz, 1H), 3.45 (d, *J* = 3.3 Hz, 1H), 3.34 (d, *J* = 3.7 Hz, 1H), 1.06 (d, J = 6.6 Hz, 3H); minor epimer δ: 8.55 (d, *J* = 4.0 Hz, 1H), 7.63 (td, *J* = 7.7, 1.5 Hz, 1H), 7.47–7.45 (m, 4H), 7.36–7.19 (m, 7H), 7.16–7.14 (m, 1H), 3.79 (dd, *J* = 5.6, 2.9 Hz, 2H), 3.24 (q, *J* = 6.6 Hz, 1H), 1.19 (d, *J* = 6.6 Hz, 3H), ^13^C-NMR (151 MHz, 283K, CDCl_3_) δ: 159.3, 154.7, 149.3, 148.8, 145.3, 145.2, 140.1, 136.5, 136.2, 133.0, 130.4, 128.31, 128.26, 128.25, 128.23, 128.1, 127.1, 127.0, 126.83, 126.81, 126.80, 126.5, 126.0, 122.2, 122.0, 120.8, 59.9, 58.1, 51.4, 51.0, 45.5, 44.9, 23.6, 23.2; HR-MS (ESI) [C_21_H_20_N_2_ + H]^+^ requires 301.1699; found 301.1710.

(2*S*,3*R*)-2-Pyridin-2-yl-3-phenyl-1-[(*S*)-1-cyclohexylethyl]-aziridine (2*S*,3*R*,1′*R*)-**11** (Method A): Two epimers on nitrogen atom in ratio 1:0.8 were observed in ^1^H-NMR and ^13^C-NMR. Colorless oil, 70 mg, 45%, ${\left[\alpha \right]}_{D}^{20}$ = 98 (c = 0.98, CHCl_3_), purified by column chromatography (Florisil, 5% AcOEt in hexane). ^1^H-NMR (600 MHz, 283K, CDCl_3_) major epimer δ: 8.62–8.61 (m, 1H), 7.69–7.65 (m, 1H), 7.49–7.26 (m, 6H), 7.22–7.18 (m, 1H), 3.77 (d, *J* = 2.2 Hz, 1H), 3.22 (d, *J* = 2.6 Hz, 1H), 2.74–2.73 (m, 1H), 2.13–2.07 (m, 1H), 1.70–1.48 (m, 5H), 1.23–1.00 (m, 5H), 0.63 (d, *J* = 6.2 Hz, 3H); minor epimer 8.59–8.58 (m, 1H), 7.75–7.70 (m, 1H), 7.49–7.26 (m, 6H), 7.22–7.18 (m, 1H), 3.44 (d, *J* = 8.8 Hz, 2H), 1.93–1.83 (m, 2H), 1.70–1.48 (m, 5H), 1.23–1.00 (m, 5H), 0.73 (d, J = 5.6 Hz, 3H), ^13^C-NMR (151 MHz, 283K, CDCl_3_) δ: 167.9, 160.02, 155.32, 155.27, 149.2, 148.6, 148.5, 140.6, 136.7, 136.0, 130.1, 128.3, 128.0, 127.7, 126.8, 126.4, 125.9, 122.0, 121.9, 120.2, 68.2, 65.7, 60.0, 58.6, 49.84, 49.79, 46.8, 46.2, 44.0, 43.8, 36.7, 30.2, 30.1, 28.6, 28.3, 26.9, 26.7, 23.7, 16.3, 15.8; HR-MS (ESI) [C_21_H_26_N_2_ + H]^+^ requires 307.2169; found 307.2173.

(2*S*,3*R*)-2-(2,2′-Bipyridin-6-yl)-3-phenyl-1-[(*S*)-1-phenylethyl]-aziridine (2*S*,3*R*,1′*S*)-**12** (Method A): We observed two epimers on nitrogen atom in ratio 1:0.3 (^1^H-NMR and ^13^C-NMR). Colorless oil, 124 mg, 66%, ${\left[\alpha \right]}_{D}^{20}$ = −59 (c = 1.01, CHCl_3_), purified by column chromatography (Florisil, 5% AcOEt in hexane). ^1^H-NMR (600 MHz, 283K, CDCl_3_) major epimer δ: 8.75–8.74 (m, 1H), 8.54 (d, *J* = 8.0 Hz, 1H), 8.29 (d, *J* = 7.7 Hz. 1H), 7.96–7.94 (m, 1H), 7.61–7.59 (m, 2H),7.55 (q, *J* = 7.7 Hz, 1H), 7.43–7.39 (m, 3H), 7.34–7.31 (m, 1H), 7.24–7.21 (m, 2H), 7.11–7.09 (m, 3H), 7.07 (d, *J* = 7.7 Hz, 1H), 4.09 (d, *J* = 2.9 Hz, 1H), 4.06 (q, *J* = 6.6 Hz, 1H), 3.34 (d, *J* = 2.9 Hz, 1H), 1.55 (d, *J* = 6.6 Hz, 3H), minor epimer δ: 8.75–8.74 (m, 1H), 8.57 (d, *J* = 7.7 Hz, 1H), 8.31 (d, *J* = 7.3 Hz, 1H), 7.90–7.87 (m, 2H), 7.61–7.59 (m, 2H), 7.43–7.39 (m, 2H), 7.34–7.31 (m, 1H), 7.24–7.21 (m, 1H), 7.11–7.09 (m, 2H), 7.07 (d, *J* = 7.7 Hz, 1H), 7.00–6.98 (m, 3H), 3.65 (d, *J* = 2.2 Hz, 1H), 3.56 (d, *J* = 2.2 Hz, 1H), 3.17 (q, J = 6.2 Hz, 1H), 1.49 (d, *J* = 6.2 Hz, 3H);^13^C-NMR (151 MHz, 283K, CDCl_3_) δ: 159.9, 156.2, 155.3, 154.2, 154.1, 149.23, 149.20, 145.2, 140.7, 137.7, 137.2, 137.0, 137.01, 137.00, 136.7, 133.5, 130.4, 128.4, 127.8, 127.7, 127.6, 127.0, 126.9, 126.7, 126.60, 126.58, 126.5, 126.4, 126.1, 123.9, 123.7, 121.4, 121.1, 120.3, 119.5, 119.0, 60.2, 58.3, 51.1, 50.9, 45.9, 45.6, 24.4, 24.3; HR-MS (ESI) [C_26_H_23_N_3_ + H]^+^ requires 378.1965; found 378.1970.

(2*R*,3*S*)-2-(2,2′-bipyridin-6-yl)-3-phenyl-1-[(*S*)-1-phenylethyl]-aziridine (2*R*,3*S*,1′*S*)-**12** (Method A): Two epimers on nitrogen atom in ratio 1:0.4 were observed in ^1^H-NMR and ^13^C-NMR. Colorless oil, 132 mg, 70%, ${\left[\alpha \right]}_{D}^{20}$ = 163 (c = 1.02, CHCl_3_), purified by column chromatography (Florisil, 5% AcOEt in hexane). ^1^H-NMR (600 MHz, 283K, CDCl_3_) major epimer δ: 8.76 (d, *J* = 4.7 Hz, 1H), 8.56 (d, *J* = 8.0 Hz, 1H), 8.54 (d, *J* = 7.7 Hz, 1H), 7.93 (qd, *J* = 7.7, 1.8 Hz, 1H), 7.89 (t, *J* = 7.7 Hz, 1H), 7.62–7.60 (m, 1H), 7.50–7.44 (m, 3H), 7.42–7.36 (m, 3H), 7.33–7.21 (m, 5H), 4.20 (q, *J* = 6.2 Hz, 1H), 3.93 (d, *J* = 2.9 Hz, 1H), 3.52 (d, *J* = 2.9 Hz, 1H), 1.08 (d, *J* = 6.6 Hz, 3H); minor epimer δ: 8.68 (d, *J* = 4.4 Hz, 1H), 8.42 (d, *J* = 8.0 Hz, 1H), 8.24 (d, *J* = 7.7 Hz, 1H), 7.81 (q, *J* = 7.7 Hz, 2H), 7.62–7.60 (m, 2H), 7.50–7.44 (m, 1H), 7.42–7.36 (m, 4H), 7.33–7.21 (m, 5H), 3.81 (d, *J* = 2.9 Hz, 1H), 3.50 (d, *J* = 3.3 Hz, 1H), 3.28 (q, *J* = 6.2 Hz, 1H), 1.19 (d, *J* = 6.2 Hz, 3H), ^13^C-NMR (151 MHz, 283K, CDCl_3_) δ: 162.0, 159.1, 156.2, 156.1, 155.3, 154.7, 153.9, 149.3, 149.1, 145.5, 145.2, 140.2, 137.6, 137.3, 137.2, 136.9, 133.1, 130.5, 128.3, 128.1, 127.1, 127.0, 126.9, 126.8, 127.8, 126.5, 126.0, 124.02, 123.7, 121.5, 121.1, 121.0, 120.5, 120.4, 119.4, 119.3, 59.9, 57.9, 51.0, 50.8, 46.0, 45.3, 23.6, 23.3; HR-MS (ESI) [C_26_H_23_N_3_ + H]^+^ requires 378.1965; found 378.1971.

### 3.3. General Procedure for the Synthesis of Cyclic Sulfamidates

The synthesis of *S*,*S*-dioxides was performed according to a modified literature procedure [39,40]. To a solution of amino alcohol **1** or **5** (1 mmol) and triethylamine (3 mmol, 0.42 mL) in dry dichloromethane (3.5 mL) was added a solution of thionyl chloride (0.8 mmol, 58 μL) in dry dichloromethane (0.25 mL) at −78 °C over 20 min. The mixture was stirred at −78 °C for 20 min and at 0 °C for the next 20 min. The reaction mixture was partitioned between ether and water, the organic layer was washed with brine and dried over anhydrous sodium sulfate, filtered and the filtrate was concentrated in vacuo. The residue was dissolved in acetonitrile (4 mL), cooled to 0 °C and NaIO_4_ (1.2 mmol, 257 mg), RuCl_3_ · 3H_2_0 (ca. 2 mg) and water (10 μL) were added. The reaction mixture was stirred at room temperature for 1 h, then diluted with water and extracted 3 × Et_2_O. The combined organic extracts were washed with brine and dried over sodium sulfate. The residue was purified by column chromatography (SiO_2_, 10% AcOEt in hexane) to provide the cyclic sulfamidate.

(5*S*)-Phenyl-3-[(*S*)-1-phenylethyl]-1,2,3-oxathiazolidine-2,2-dioxide (5*S*,1′*S*)-**17**: White solid, 151 mg, 50%, m.p. 100–101 °C, ${\left[\alpha \right]}_{D}^{20}$ = −73 (c = 0.62 CHCl_3_), ^1^H-NMR (400 MHz, CDCl_3_) δ: 7.39–7.33 (m, 10H), 5.62 (dd, *J* = 10.1, 6.1 Hz, 1H), 4.35 (q, *J* = 6.4 Hz, 1H), 3.40 (dd, *J* = 10.1, 6.1 Hz, 1H), 3.19 (t, *J* = 9.8 Hz, 1H), 1.75 (d, *J* = 6.7 Hz, 3H); ^13^C-NMR (101 MHz, CDCl_3_) δ: 140.7, 134.7, 129.9, 129.1, 129.0, 128.6, 127.1, 126.5, 80.9, 59.1, 55.3, 20.8, HR-MS (ESI) [C_16_H_17_NO_3_S + Na]^+^ requires 326.0822; found 326.0826.

(4*S*,5*S*)-4-Phenyl-5-(pyridin-2-yl)-3-[(*S*)-1-phenylethyl]-1,2,3-oxathiazolidine-2,2-dioxide (4*S*,5*S,*1′*S*)-**18**: White solid, 288 mg, 76%, m.p. 68–70 °C, ${\left[\alpha \right]}_{D}^{20}$ = −11 (c = 0.73 CHCl_3_), ^1^H-NMR (400 MHz, CDCl_3_) δ: 8.32 (d, *J* = 4.9 Hz, 1H), 7.36–7.26 (m, 6H), 7.03–6.94 (m, 7H), 5.97 (d, *J* = 6.1 Hz, 1H), 4.96 (q, *J* = 6.7 Hz, 1H), 4.75 (d, *J* = 6.4 Hz, 1H), 1.50 (d, *J* = 6.7 Hz, 3H); ^13^C-NMR (101 MHz, CDCl_3_) δ: 152.6, 148.7, 138.5, 136.5, 136.1, 128.7, 128.6, 128.1, 128.0, 127.9, 126.9, 123.1, 120.9, 83.1, 65.8, 56.4, 20.2; HR-MS (ESI) [C_21_H_20_N_2_O_3_S + Na]^+^ requires 403.1087; found 403.1084.

(4*R*,5*R*)-4-Phenyl-5-(pyridin-2-yl)-3-[(*S*)-1-phenylethyl]-1,2,3-oxathiazolidine-2,2-dioxide (4*R*,5*R,*1′S)-**18**: White solid, 296 mg, 79%, ${\left[\alpha \right]}_{D}^{20}$ = −7 (c = 0.89 CHCl_3_), ^1^H-NMR (400 MHz, CDCl_3_) δ: 8.32 (d, *J* = 4.9 Hz, 1H), 7.37 (td, J = 7.6, 1.5 Hz, 1H), 7.28–7.21 (m, 5H), 7.01–6.97 (m, 7H), 6.08 (d, *J* = 6.1 Hz, 1H), 4.82 (d, *J* = 6.1 Hz, 1H), 4.33 (q, *J* = 6.7 Hz, 1H), 1.85 (d, *J* = 7.0 Hz, 3H); ^13^C-NMR (101 MHz, CDCl_3_) δ: 152.7, 148.7, 140.5, 136.6, 133.5, 128.9, 128.4, 128.33, 128.32, 128.2, 127.2, 123.2, 120.7, 82.5, 67.2, 57.0, 20.1; HR-MS (ESI) [C_21_H_20_N_2_O_3_S + H]^+^ requires 381.1261; found 381.1265.

### 3.4. Synthesis of Azides

**Method 1:** Synthesis of azides by ring opening of aziridines 

The solution of HN_3_ (1.53 M in C_6_H_6_, 0.14 mmol, 200 μL) was added to the aziridines (0.07 mmol) by a syringe and the reaction mixture was stirred at RT overnight. Then, the solvent was evaporated under reduced pressure to give the corresponding azide.

**Method 2:** Synthesis of azides by nucleophilic substitution of sulfamidates

The synthesis was performed according to a modified literature procedure [40]. To a solution of *S*,*S*-dioxide (0.5 mmol) in dry DMF (1.5 mL) sodium azide (2.5 mmol, 162 mg) was added and the mixture was stirred overnight at room temperature. The reaction mixture was concentrated in vacuo*,* and ether (1.6 mL) and 20% H_2_SO_4_ were added. The mixture was stirred for 5 h in room temperature, then neutralized with NaHCO_3_ and extracted 3× CHCl_3_. The combined organic layer was dried over sodium sulfate and concentrated. The residue was chromatographed on silica gel (10% AcOEt in hexane) to give a corresponding product.

**Method 3:** Synthesis of azides by Mitsunobu reaction conditions with hydrazoic acid

The synthesis was performed according to a modified literature procedure [18]. To a solution of amino alcohols **1**, or **2** (0.5 mmol) and triphenylphosphine (0.65 mmol, 170 mg) in dry benzene (1.6 mL) was added HN_3_ (1.53 M in C_6_H_6_, 0.65 mmol, 0.7 mL), and next solution of DEAD (0.75 mmol, 116 μL) in benzene (1 mL) at 0 °C. The reaction mixture was stirred at room temperature for 3 h (for amino alcohol **1**) or 24 h (for **2**). Then, the solvent was removed and the products were isolated by column chromatography (Florisil, 10% AcOEt in hexane).

*N*-[(*S*)-1-Phenylethyl]-(*R*)-2-azido-2-phenyl-ethylamine (1*R*,1′*S*)-**3** (Methods 1 and 2): Yellow oil, 18 mg, 97% (method 1), ${\left[\alpha \right]}_{D}^{20}$ = −129 (c = 1.02, CHCl_3_), ^1^H-NMR (600 MHz, CDCl_3_) δ: 7.38–7.24 (m, 10H), 4.65 (t, *J* = 6.6 Hz, 1H), 3.81 (q, *J* = 6.6 Hz, 1H), 2.74 (d, *J* = 2.9 Hz, 2H), 1.36 (d, *J* = 6.6 Hz, 3H); ^13^C-NMR (151 MHz, CDCl_3_) δ: 145.1, 138.1, 128.8, 128.6, 128.4, 127.1, 127.0, 126.5, 66.1, 57.8, 52.9, 24.4; HR-MS (ESI) [C_16_H_18_N_4_ + H]^+^ requires 267.1604; found 267.1604.

*N*-[(*S*)-1-Phenylethyl]-(*S*)-2-azido-2-phenyl-ethylamine (1*S*,1′*S*)-**3** (Method 1): Yellow oil, 60 mg, 90% (method 2), ${\left[\alpha \right]}_{D}^{20}$ = 97 (c = 0.91, CHCl_3_), ^1^H-NMR (600 MHz, CDCl_3_) δ: 7.37–7.30 (m, 7H), 7.27–7.23 (m, 3H), 4.61–4.58 (m, 1H), 3.84 (q, *J* = 6.6 Hz, 1H), 2.82 (dd, *J* = 12.5, 9.2 Hz, 1H), 2.69 (dd, J = 12.5, 4.8 Hz, 1H), 1.40 (d, *J* = 6.6 Hz, 3H); ^13^C-NMR (150 MHz, CDCl_3_) δ: 137.9, 128.9, 128.6, 128.5, 128.0, 127.2, 127.1, 126.7, 66.3, 58.5, 52.9, 24.3; HR-MS (ESI) [C_16_H_18_N_4_ + H]^+^ requires 267.1604; found 267.1606.

*N*-[(*S*)-1-Phenylethyl]-(*R/S*)-2-azido-2-phenyl-ethylamine (1*R*,1′*S*)-**3** and (1S,1′*S*)-**3** in ratio 0.8:1 (Method 3): Yellow oil, 106 mg, 40%, HR-MS (ESI) [C_16_H_18_N_4_ + H]^+^ requires 267.1604; found 267.1606.

*N*-[(*S*)-1-Phenylethyl]-(*S*)-2-azido-2-pyridin-2-yl-ethylamine (1*S*,1′*S*)-**4** (Methods 1 and 3): Yellow oil, 16 mg, 84% (method 1), ${\left[\alpha \right]}_{D}^{20}$ = 24 (c = 0.91 CHCl_3_), ^1^H-NMR (400 MHz, CDCl_3_) δ: 8.55 (d, *J* = 4.9 Hz, 1H), 7.70–7.66 (m, 1H), 7.34–7.19 (m, 7H), 4.69 (t, *J* = 7.6 Hz, 1H), 3.85 (q, *J* = 6.7 Hz, 1H), 2.95–2.91 (m, 2H), 1.39 (d, *J* = 6.7 Hz, 3H); ^13^C-NMR (101 MHz, CDCl_3_) δ: 157.7, 149.6, 137.1, 128.7, 127.3, 126.8, 123.3, 121.94, 121.87, 66.0, 58.5, 51.3, 24.2; HR-MS (ESI) [C_15_H_17_N_5_ + H]^+^ requires 268.1557; found 268.1559.

*N*-[(*S*)-1-Phenylethyl)-(1*R*,2*R*)-2-azido-2-phenyl-1-pyridin-2-yl-ethylamine (1*R*,2*R*,1′*S*)-**13** (Method 1): Yellow oil, 23 mg, 96%, ${\left[\alpha \right]}_{D}^{20}$ = −57 (c = 1.05, CHCl_3_), ^1^H-NMR (600 MHz, CDCl_3_) δ: 8.60 (d, *J* = 4.7 Hz, 1H), 4.78 (qd, *J* = 7.4, 1.5 Hz, 1H), 7.37–7.32 (m, 3H), 7.27–7.25 (m, 2H), 7.20–7.13 (m, 4H), 7.08–7.06 (m, 2H), 6.88 (d, *J* = 7.7 Hz, 1H), 4.95 (d, *J* = 7.7 Hz, 1H), 3.95 (d, *J* = 7.7 Hz, 1H), 3.47 (q, *J* = 6.2 Hz, 1H), 1.18 (d, *J* = 6.2 Hz, 3H); ^13^C-NMR (151 MHz, 283K, CDCl_3_) δ: 160.0, 149.4, 145.6, 137.7, 135.9, 128.5, 128.3, 128.2, 127.9, 126.8, 126.6, 123.6, 122.4, 70.0, 65.7, 56.0, 23.2; HR-MS (ESI) [C_21_H_21_N_5_ + H]^+^ requires 344.1870; 344.1880.

*N*-[(*S*)-1-Phenylethyl]-(1*S*,2*S*)-2-azido-1-[2,2′-bipyrid-6-yl]-2-phenyl-ethylamine (1*S*,2*S*,1′*S*)-**14** (Method 1): Yellow oil, 23 mg, 94%, ${\left[\alpha \right]}_{D}^{20}$ = 27 (c = 1.1, CHCl_3_), ^1^H-NMR (600 MHz, CDCl_3_) δ: 8.72 (d, *J* = 4.7 Hz, 1H), 8.49 (dt, *J* = 8.1, 1.1 Hz, 1H), 8.37 (dd, *J* = 7.7, 1.1 Hz, 1H), 7.87–7.84 (m, 1H), 7.76 (q, *J* = 7.7 Hz, 1H), 7.38–7.33 (m, 4H), 7.23–7.20 (m, 5H), 7.02 (d, *J* = 7.7 Hz, 1H), 6.95–6.93 (m, 2H), 4.86 (d, *J* = 8.4 Hz, 1H), 3.72 (d, J = 8.4 Hz, 1H), 3.44 (q, *J* = 6.6 Hz, 1H), 1.15 (d, *J* = 6.6 Hz, 3H); ^13^C-NMR (151 MHz, 283K, CDCl_3_) δ: 149.4, 156.3, 156.1, 149.2, 144.9, 137.8, 136.9, 136.8, 128.4, 128.31, 128.28, 128.2, 127.0, 126.9, 124.3, 123.8, 121.3, 119.9, 70.2, 64.9, 55.6, 25.4; HR-MS (ESI) [C_26_H_24_N_6_ + H]^+^ requires 421.2135; 421.2147.

*N*-[(*S*)-1-Phenylethyl]-(1*S*,2*R*)-2-azido-1-phenyl-2-pyridin-2-yl-ethylamine (1*R*,2*S*,1′*S*)-**19** (Method 2): Colorless oil, 120 mg, 70%, ${\left[\alpha \right]}_{D}^{20}$ = −4 (c = 0.54 CHCl_3_), ^1^H-NMR (400 MHz, CDCl_3_) δ: 8.57 (d, *J* = 4.6 Hz, 1H), 7.51 (td, J = 7.6, 1.8 Hz, 1H), 7.26–7.10 (m, 11H), 7.00–6.98 (m, 1H), 4.64 (d, *J* = 7.0 Hz, 1H), 4.34 (d, *J* = 7.0 Hz, 1H), 3.60 (q, *J* = 6.4 Hz, 1H), 1.27 (d, *J* = 6.4 Hz, 3H); ^13^C-NMR (101 MHz, CDCl_3_) δ: 157.3, 149.4, 146.1, 140.0, 136.3, 128.4, 128.3, 127.9, 127.5, 126.9, 126.7, 123.0, 122.9, 72.1, 64.0, 54.8, 22.0; HR-MS (ESI) [C_21_H_21_N_5_ + H]^+^ requires 344.1870; found 344.1867.

*N*-[(*S*)-1-Phenylethyl]-(1*R*,2*S*)-2-azido-1-phenyl-2-pyridin-2-yl-ethylamine (1S,2*R*,1′*S*)-**19** (Methods 2 and 3): Colorless oil, 125 mg, 70% (Method 2), ${\left[\alpha \right]}_{D}^{20}$ = −116 (c = 0.90 CHCl_3_), ^1^H-NMR (600 MHz, CDCl_3_) δ: 8.47 (d, *J* = 4.8 Hz, 1H), 7.54 (td, J = 7.3, 1.5 Hz, 1H), 7.26–7.12 (m, 9H), 7.05–7.04 (m, 1H), 6.96–6.94 (m, 2H), 4.60 (d, *J* = 6.6 Hz, 1H), 3.86 (d, *J* = 6.6 Hz, 1H), 3.47 (q, *J* = 6.6 Hz, 1H), 1.29 (d, *J* = 6.6 Hz, 3H); ^13^C-NMR (101 MHz, CDCl_3_) δ: 157.2, 149.2, 145.0, 139.9, 136.2, 128.4, 128.3, 128.0, 127.5, 126.8, 126.5, 122.8, 122.7, 72.2, 63.3, 54.7, 25.1; HR-MS (ESI) [C_21_H_21_N_5_ + H]^+^ requires 344.1870; found 344.1876.

### 3.5. General Procedure for the Synthesis of Diamines

The synthesis of diamines was performed according to a modified literature procedure [40]. To a solution of azide (0.05 mmol) in CH_2_Cl_2_ (0.05 mL) was added triphenylphosphine (0.055 mmol, 15 mg) and water (11 μL). The reaction mixture was stirred overnight and the solvent was removed under vacuum. Then the product was isolated from the crude mixture by column chromatography (Florisil, CHCl_3_:AcOEt:MeOH 1:1:0.25).

*N*-[(*S*)-1-Phenylethyl]-(1*R*)-phenyl-1,2-ethanediamine (1*R*,1′*S*)-**20**: Colorless oil, 75%, ${\left[\alpha \right]}_{D}^{20}$ = −97 (c = 0.96 CHCl_3_), ^1^H-NMR (400 MHz, CDCl_3_) δ: 7.23–7.19 (m, 10H), 4.0 (dd, *J* = 8.6, 4.6 Hz, 1H), 3.76 (q, *J* = 6.7 Hz, 1H), 2.72 (dd, *J* = 11.6, 4.3 Hz, 1H), 2.51 (dd, *J* = 11.9, 8.9 Hz, 1H), 1.34 (d, *J* = 6.4 Hz, 3H); ^13^C-NMR (101 MHz, CDCl_3_) δ: 143.4, 128.8, 128.7, 128.6, 127.6, 127.5, 126.9, 126.4, 58.3, 55.0, 54.6, 23.7; HR-MS (ESI) [C_16_H_20_N_2_ + H]^+^ requires 241.1699; found 241.1696.

*N*-[(*S*)-1-Phenylethyl]-(1*S*)-pyridin-2-yl-1,2-ethanediamine (1*S*,1′*S*)-**21**: Colorless oil, 75%, ${\left[\alpha \right]}_{D}^{20}$ = −27 (c = 0.67 CHCl_3_), ^1^H-NMR (600 MHz, CDCl_3_) δ: 8.55 (d, *J* = 4.8 Hz, 1H), 7.65 (td, *J* = 7.7, 1.8 Hz, 1H), 7.37–7.24 (m, 6H), 7.18 (dd, *J* = 4.8, 1.1 Hz, 1H), 4.15–4.13 (m, 1H), 3.88 (q, *J* = 6.7 Hz, 1H), 2.84 (dd, *J* = 11.7, 4.8 Hz, 1H), 2.76 (dd, *J* = 11.7, 8.0 Hz, 1H), 1.47 (d, *J* = 6.6 Hz, 1H); ^13^C-NMR (151 MHz, CDCl_3_) δ: 162.4, 149.1, 144.2, 136.7, 128.6, 127.3, 126.8, 122.3, 121.6, 58.7, 56.0, 54.0, 23.8; HR-MS (ESI) [C_15_H_19_N_3_ + H]^+^ requires 242.1652; found 1649.

*N*-[(*S*)-1-Phenylethyl]-(1*R*,2*R*)-1-pyridin-2-yl-2-phenyl-1,2-ethanediamine (1*R*, 2*R*, 1′*S*)-**22**: Colorless oil, 60%, ${\left[\alpha \right]}_{D}^{20}$ = −31 (c = 0.49 CHCl_3_), ^1^H-NMR (400 MHz, CDCl_3_) δ: 8.52 (d, *J* = 4.9 Hz, 1H), 7.39 (td, *J* = 7.6, 1.8 Hz, 1H), 7.24–7.14 (m, 8H), 7.09–7.05 (m, 3H), 6.71 (d, *J* = 7.6 Hz, 1H), 4.34 (d, *J* = 6.1 Hz, 1H), 3.94 (d, *J* = 6.1 Hz, 1H), 3.94 (d, *J* = 6.1 Hz. 1H), 3.62 (q, *J* = 6.4 Hz, 1H), 1.26 (d, *J* = 6.4 Hz, 3H); ^13^C-NMR (101 MHz, CDCl_3_) δ: 160.8, 149.1, 145.9, 143.3, 135.8, 128.30, 128.28, 128.2, 127.2, 127.1, 126.8, 123.6, 122.1, 67.1, 59.8, 55.9, 23.3; HR-MS (ESI) [C_21_H_23_N_3_ + H]^+^ requires 318.1965; found 318.1971.

*N*-[(*S*)-1-Phenylethyl]-(1*S*,2*S*)-1-(2,2′-bipyridin-6-yl)-2-phenyl-1,2-ethanediamine (1*S*,2*S*,1′*S*)-**23**: Colorless oil, 50%, ${\left[\alpha \right]}_{D}^{20}$ = −30 (c = 0.43 CHCl_3_), ^1^H-NMR (400 MHz, CDCl_3_) δ: 8.67 (d, *J* = 4.9 Hz, 1H), 8.33 (d, *J* = 7.9 Hz, 1H), 8.28 (d, *J* = 7.9 Hz, 1H), 7.81 (td, *J* = 7.3, 1.8 Hz, 1H), 7.65 (t, *J* = 7.6 Hz, 1H), 7.32–7.16 (m, 7H), 7.10–7.08 (m, 2H), 7.04–7.01 (m, 2H), 6.86 (d, *J* = 7.6 Hz, 1H), 4.29 (d, *J* = 6.4 Hz, 1H), 3.73 (d, *J* = 6.4 Hz, 1H), 3.46 (q, *J* = 6.4 Hz, 1H), 1.23 (d, *J* = 6.7 Hz, 3H); ^13^C-NMR (101 MHz, CDCl_3_) δ: 156.4, 155.7, 149.2, 145.2, 143.1, 137.0, 136.9, 132.1, 128.5, 128.4, 128.0, 127.4, 127.1, 127.0, 124.2, 123.8, 121.3, 119.5, 66.3, 60.6, 55.8, 25.4; HR-MS (ESI) [C_26_H_26_N_4_ + H]^+^ requires 395.2230; found 395.2256.

*NN*-[(*S*)-1-Phenylethyl]-(1*S*,2*R*)-1-pyridin-2-yl-2-phenyl-1,2-ethanediamine (1*S*,2*R*,1′*S*)-**24**: Yellow oil, 12 mg, 73%, ${\left[\alpha \right]}_{D}^{20}$ = −45 (c = 0.52 CHCl_3_), ^1^H-NMR (400 MHz, CDCl_3_) δ: 8.43 (d, *J* = 4.9 HZ, 1H), 7.32 (td, *J* = 7.6, 1.8, Hz, 1H), 7.25–7.16 (m, 7H), 7.09–7.07 (m, 2H), 7.03–7.00 (m, 3H), 3.99 (d, *J* = 7.6 Hz, 1H), 3.51–3.48 (m, 2H), 1.31 (d, *J* = 6.7 Hz, 3H); ^1^^3^C-NMR (101 MHz, CDCl_3_) δ: 148.8, 144.9, 135.8, 128.54, 128.47, 128.3, 127.9, 127.8, 127.2, 126.9, 126.7, 122.9, 122.0, 65.7, 62.4, 55.0, 25.0; HR-MS (ESI) [C_21_H_23_N_3_ + H]^+^ requires 318.1965; found 318.1972.

*N*-[(*S*)-1-Phenylethyl]-(1*R*,2*S*)-1-pyridin-2-yl-2-phenyl-1,2-ethanediamine (1*R*, 2*S*, 1′*S*)-**24**: Yellow oil, 11 mg, 70%, ${\left[\alpha \right]}_{D}^{20}$ = −83 (c = 0.87 CHCl_3_), ^1^H-NMR (600 MHz, CDCl_3_) δ: ^1^H-NMR (600 MHz, CDCl_3_) δ: 8.56 (d, *J* = 4.8 HZ, 1H), 7.40 (td, *J* = 7.3, 1.8, Hz, 1H), 7.29–7.08 (m, 8H), 7.10–7.08 (m, 3H), 6.79–6.78 (m, 1H), 4.08 (d, *J* = 8.0 Hz, 1H), 4.04 (d, *J* = 7.7 Hz, 1H), 3.63 (q, *J* = 6.6 Hz, 1H), 2.90 (s, 3H), 1.35 (d, *J* = 6.6 Hz, 3H) ^1^^3^C-NMR (151 MHz, CDCl_3_) δ: 161.0; 149.0, 145.8, 141.0, 135.9, 128.3, 128.1, 127.7, 127.1, 126.9, 126.7, 123.1, 122.2, 66.4, 62.6, 54.9, 21.8; HR-MS (ESI) [C_21_H_23_N_3_ + H]^+^ requires 318.1965; found 318.1966.

## 4. Conclusions

**Scheme 4:**
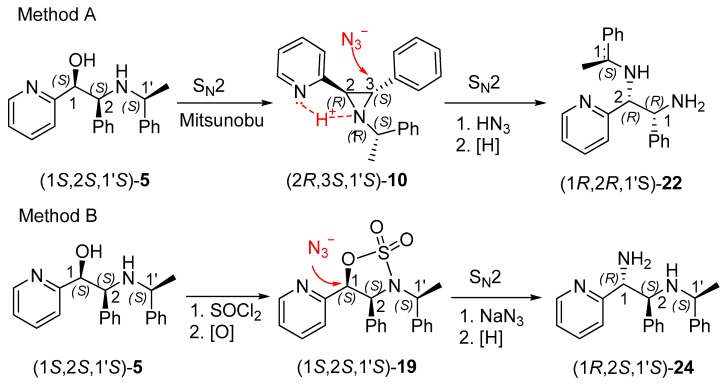
Stereodivergent synthesis of chiral *vic*-diamines as exemplified by the transformations of (1*S*,2*S*,1′*S*)-5.

We developed two different transformations of pyridine-containing chiral β-amino alcohols to the respective isomeric *vic*-diamines (Scheme 4). The first method used an internal Mitsunobu S_N_2 reaction to furnish intermediate aziridine, followed by the ring opening with hydrazoic acid and Staudinger reduction (Scheme 4, method A). The 1,2,3-trisubstituted aziridines exist as observable N-epimeric forms that exhibit disparate affinity towards zinc ions in the NMR. For these aziridines ring closure and ring opening occurred at different carbon stereocenters, thus yielding products with two inverted configurations, compared to the starting amino alcohol. The protonation likely activates the respective carbon in the aziridine ring leading to the observed regioselectivity. The 1,2-disubstituted aziridines produced diamines of the same configuration as the starting β-amino alcohols because of double inversion at the same stereogenic center. 

In the second method (Scheme 4, method B) we adopted the formation of cyclic sulfamidate, which reacted with sodium azide in S_N_2 manner and the obtained azide was reduced, thus providing products with the inverted configuration at one stereogenic center. The usefulness of both methods is documented by a high-yielding preparation of 6 new chiral *vic*-diamines.

## Figures and Tables

**Table 1:**
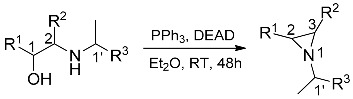
Synthesis of aziridines by Mitsunobu reaction (Methods A and B).

Configuration of Amino Alcohol	R^1^	R^2^	R^3^	Configuration of Products	Yield ^a^ (%)
(1*R*,1′*S*)-**1**	Ph	H	Ph	(2*S*,1′*S*)-**8**	76
(1S,1′*S*)-**1**	Ph	H	Ph	(2*R*,1′*S*)-**8**	72
(1*S*,1′*S*)-**2**	Py	H	Ph	(2*R*,1′*S*)-**9**	45, 82 ^b^
(1*S*,2*S*,1′*S*)-**5**	Py	Ph	Ph	(2*R*,3*S*,1′*S*)-**10**	33, 74 ^b^
(1*R*,2*R*,1′*S*)-**5**	Py	Ph	Ph	(2*S*,3*R,*1′*S*)-**10**	33, 76 ^b^
(1*R*,2*R*,1′*R*)-**6**	Py	Ph	cyclohexyl	(2*S*,3*R*,1′*R*)-**11**	45
(1*S*,2*S*,1′*S*)-**7**	Bpy	Ph	Ph	(2*R*,3*S*,1′*S*)-**12**	65
(1*R*,2*R*,1′*S*)-**7**	Bpy	Ph	Ph	(2*S*,3*R*,1′*S*)-**12**	70

^a^ Method A: To the solution of amino alcohol in Et_2_O 1.5 eq of PPh_3_ and DEAD was added, the mixture was stirred under argon atmosphere overnight. ^b^ Method B: An additional 0.5 eq of PPh_3_ and DEAD was added and the mixture was stirred overnight. For the details, see Experimental. Py = pyridin-2-yl, Bpy = 2,2′-bipyridin-6-yl.

**Table 2:** Comparison of experimental and DFT calculated NMR chemical shifts (GIAO DFT/mPW1PW91/6-311+G(2d,p)) for (2*S*,3*R*,1′*S*)-**10**.

Signal	DFT *δ*, ppm		Experiment *δ*, ppm	
	(1*S*_N_)-10	(1*R*_N_)-10	Major	Minor
H-2	3.16	3.74	3.27	3.52
H-3	4.20	3.49	3.97	3.49
1-CH	4.75	3.10	3.91	3.12
CH_3_	1.59	1.46	1.50	1.44

**Table 3:**
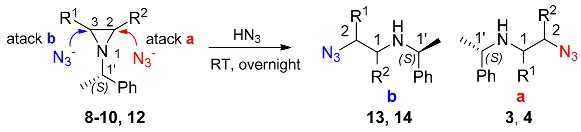
Regioselective ring opening of aziridines with HN_3._

Configuration of Aziridines	R^1^	R^2^	Configuration of Azides	Yield of Azides (%)
(2*S*,1′*S*)-**8**	H	Ph	(1*R*,1′*S*)-**3**	97
(2*R*,1′*S*)-**8**	H	Ph	(1*S*,1′*S*)-**3**	90
(1*R*,1′*S*)-**9**	H	Py	(2*S*,1′*S*)-**4**	93 ^a^
(2*R*,3*S*,1′*S*)-**10**	Ph	Py	(1*R*,2*R*,1′*S*)-**13**	95
(2S,3*R*,1′*S*)-**12**	Ph	Bpy	(1*S*,2*S*,1′*S*)-**14**	94

^a^ For the ring opening of aziridine **9** in the crude mixture 10% of minor regioisomer was identified in NMR. Yield is given for the mixture.

**Table 4:**
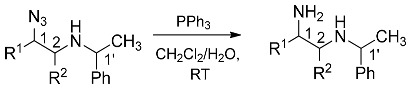
Synthesis of diamines via the Staudinger reaction.

Configuration of Azides	R^1^	R^2^	Configuration of Diamines	Yield of Diamines (%)
(1*R*,1′*S*)-**3**	Ph	H	(1*R*,1′*S*)-**20**	74
(1*S*,1′*S*)-**4**	Py	H	(1*S*,1′*S*)-**21**	75
(1*R*,2*R*,1′*S*)-**13**	Ph	Py	(1*R*,2*R*,1′*S*)-**22**	60
(1*S*,2*S*,1′*S*)-**14**	Ph	Bpy	(1*S*,2*S*,1′*S*)-**23**	50
(1*R*,2*S*,1′*S*)-**19**	Py	Ph	(1*R*,2*S*,1′*S*)-**24**	70
(1*S*,2*R*,1′*S*)-**19**	Py	Ph	(1*S*,2*R*,1′*S*)-**24**	73

## Supplementary Materials

The following are available online, NMR assignments for azido amines, DFT computation details, additional NMR experiments, copies of ^1^H and ^13^C-NMR spectra.
