# Supplementary material for: Regioselective and Stereodivergent Synthesis of Enantiomerically Pure Vic-Diamines from Chiral β-Amino Alcohols with 2-Pyridyl and 6-(2,2′-Bipyridyl) Moieties
Source: Molecules. 2020 Feb 7;25(3):727. doi: 10.3390/molecules25030727 (PMC7037692; doi:10.3390/molecules25030727)
Supplement: Supplementary file 1 [file molecules-25-00727-s001.pdf]

molecules

Supporting information

Regioselective and stereodivergent synthesis of enantiomerically pure vic-diamines from chiral β-amino alcohols with 2-pyridyl and 6-(2,2′-bipyridyl) moieties

Marzena Wosińska-Hrydczuk [1], Przemyslaw J. Boratyński [1], and Jacek Skarżewski [1]*

[1] Department of Organic Chemistry, Faculty of Chemistry, Wrocław University of Technology, Wyb. Wyspiańskiego 27, 50-370 Wrocław, Poland,

* Correspondence: jacek.skarzewski@pwr.edu.pl; Tel.: +48 71 320 2464

Contents

1. Structure of ring opening products 4

3. NMR spectra for titration of (1*S*/1*R*,2*S*,3*R*)-**10** and (1*S*/1*R*,2*R*,3*S*)-**12** with zinc acetate 8

4. EXSY experiment for (2*S*,3*R*,1’*S*)-**10** and ^{1}H,^{13}C HSQC and HMBC spectra for azides **3**, **13**, **14** and **19** 10

5. Copies of NMR Spectra 14

6. DFT/B3LYP/CC-pVDZ geometries listings 61

1. Structure of ring opening products

The structures of the azides (1*R*,1’*S*)-**3a**, (1*R*,2*R*,1’*S*)-**13**, (1S,2S,1’S)-**14**, (1*S*,2*R*,1’*S*)-**19** and (1*R*,2*S*,1’*S*)-**19** and thus the regiochemistry of the reactions was determined by a stet of NMR experiments, where the $^{1}H,^{13}C$ HMBC proved to be most revealing.

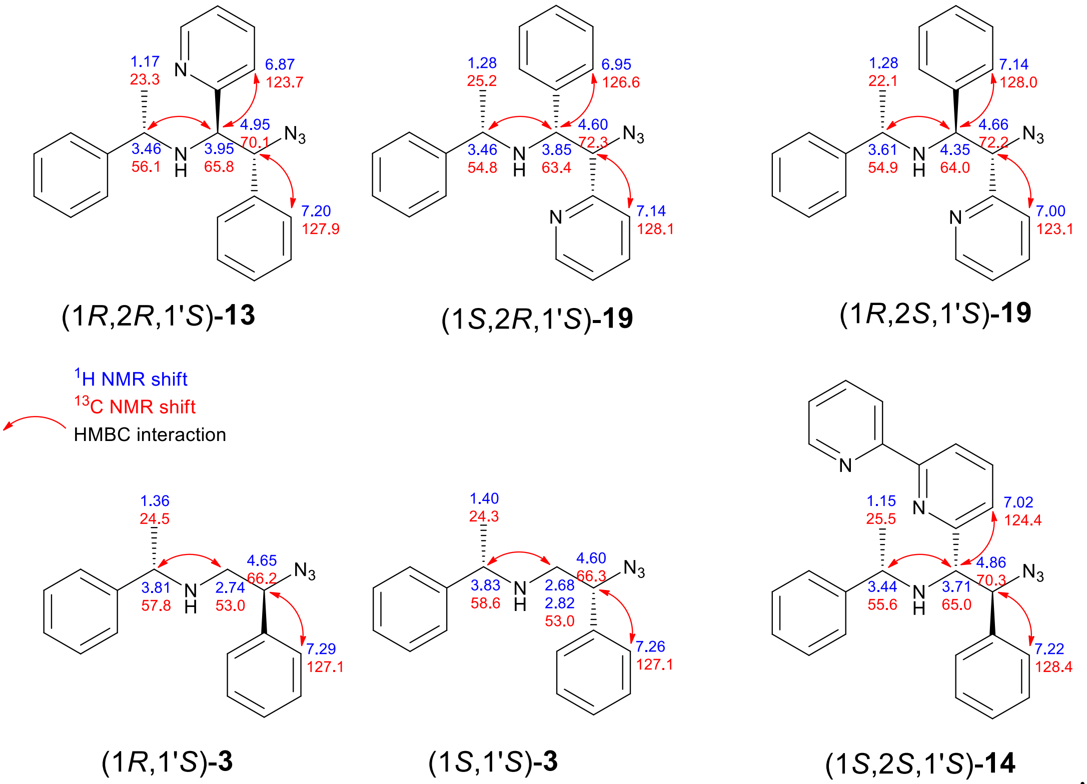

Figure S1. Assignment of selected ^{1}H and ^{13}C NMR signals and $^{1}H,^{13}C$ HMBC correlations for the establishment of regiochemistry.

2. DFT computations for aziridines **10** and **12**

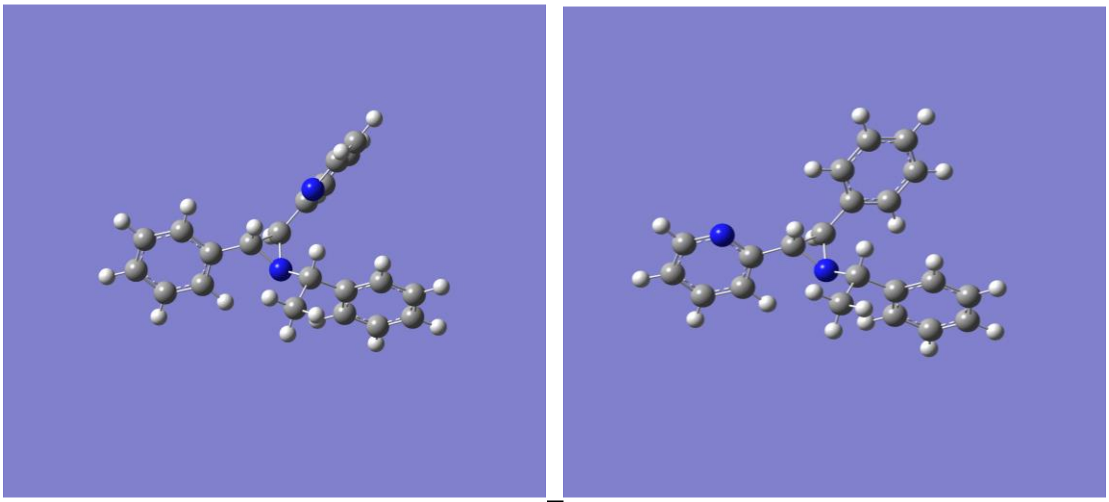

Figure S2. Molecular geometry of lowest energy structures optimized at the DFT/B3LYP/CC-pVDZ level for (1*S*,2*S*,3*R*)-**10**(left) and (1*R*,2*S*,3*R*)-**10** (right).

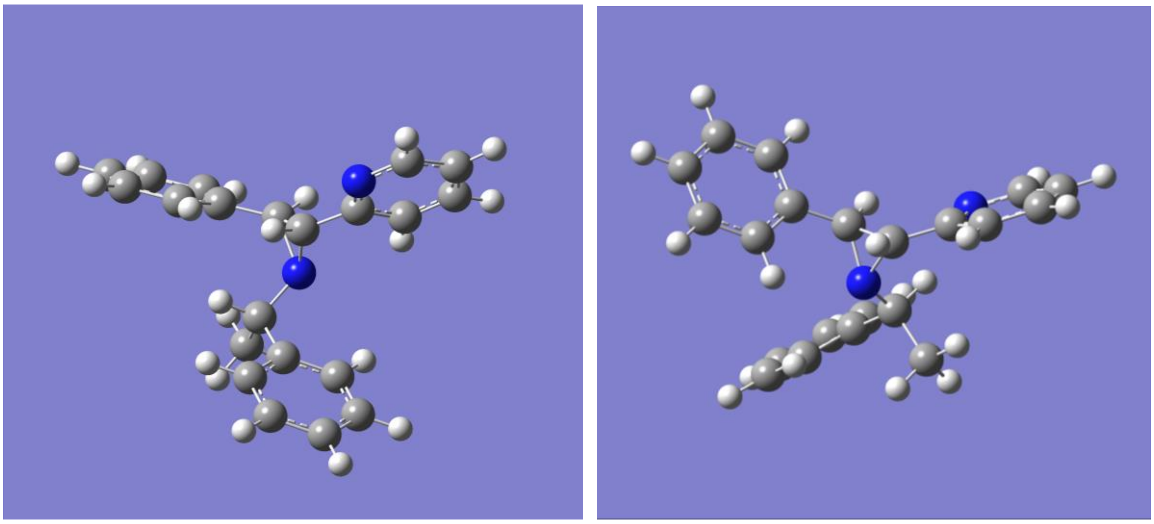

Figure S3. Molecular geometry of lowest energy structures optimized at the DFT/B3LYP/CC-pVDZ level for (1*S*,2*R*,3*S*)-**10** (left) and (1*S*,2*R*,3*S*)-**10** (right).

Table S1. Comparison of experimental and DFT calculated NMR chemical shifts (GIAO DFT/mPW1PW91/6-311+G(2d,p)) for (2*S*,3*R*,1'*S*)-**10**

2*S*,3*R*-**10** Signal	DFT *δ*, ppm		Experiment *δ*, ppm	
	$1S^N$	$1R^N$	Major (52%)	Minor (48%)
H-2	3.68	3.36	3.45	3.34
H-3	3.76	3.89	3.79	3.78
H-1'	3.56	4.62	3.99	3.24
1'-CH_3	1.21	1.22	1.06	1.18

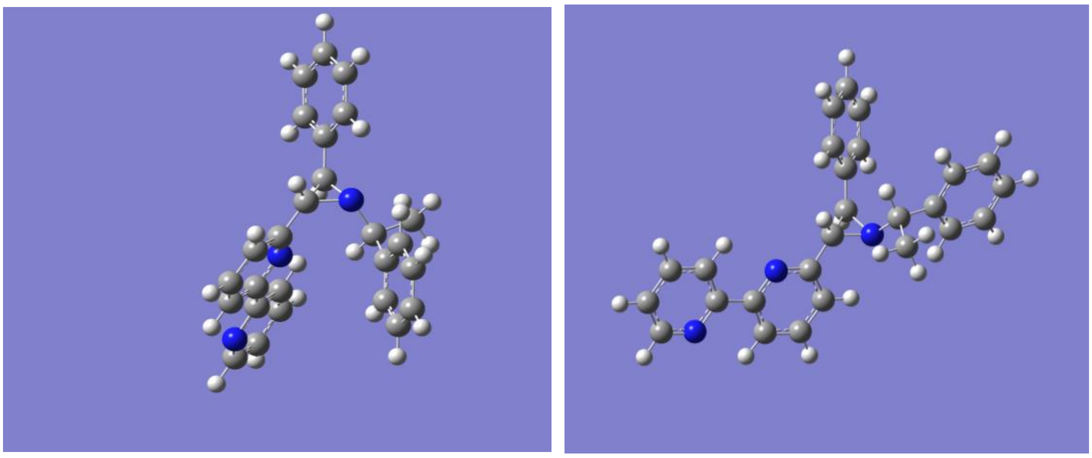

Figure S4. Molecular geometry of lowest energy structures optimized at the DFT/B3LYP/CC-pVDZ level for (1*S*,2*S*,3*R*)-**12** (left) and (1*R*,2*S*,3*R*)-**12** (right).

Table S2. Comparison of experimental and DFT calculated NMR chemical shifts (GIAO DFT/mPW1PW91/6-311+G(2d,p)) for (2*S*,3*R*,1'S)-**12**

2*R*,3*S*-**12** Signal	DFT δ, ppm		Experiment δ, ppm	
	1*S*^{N}	1*R*^{N}	Major (76%)	Minor (24%)
H-2	3.29	4.03	3.33	3.64
H-3	4.32	3.53	4.09	3.56
H-1'	4.52	3.61	4.05	3.17
1'-CH_3	1.63	1.59	1.55	1.48

Table S3. Comparison of experimental and DFT calculated NMR chemical shifts (GIAO DFT/mPW1PW91/6-311+G(2d,p)) for 2*S*,3*R*-**10**·Zn(OAc)$_2$

	DFT, δ (ppm)	Experiment, δ (ppm)
H-2	3.41	4.00
H-3	4.52	4.74
H-1'	5.87	3.64
1'-CH_3	1.70	1.61

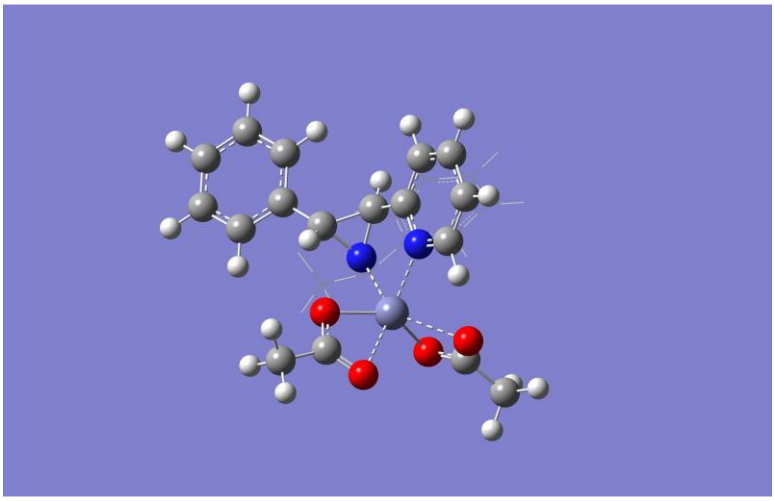

Figure S5. DFT computed structure of zinc ligand (1*S*,2*S*,3*R*)-**10** acetate complex. For clarity, methylbenzyl group is shown as wireframe, Zn-heteroatom distances of 2.15-2.51 Å are shown as dashed lines.

3. NMR spectra for titration of (1*S*/1*R*,2*S*,3*R*)-**10** and (1*S*/1*R*,2*R*,3*S*)-**12** with zinc acetate

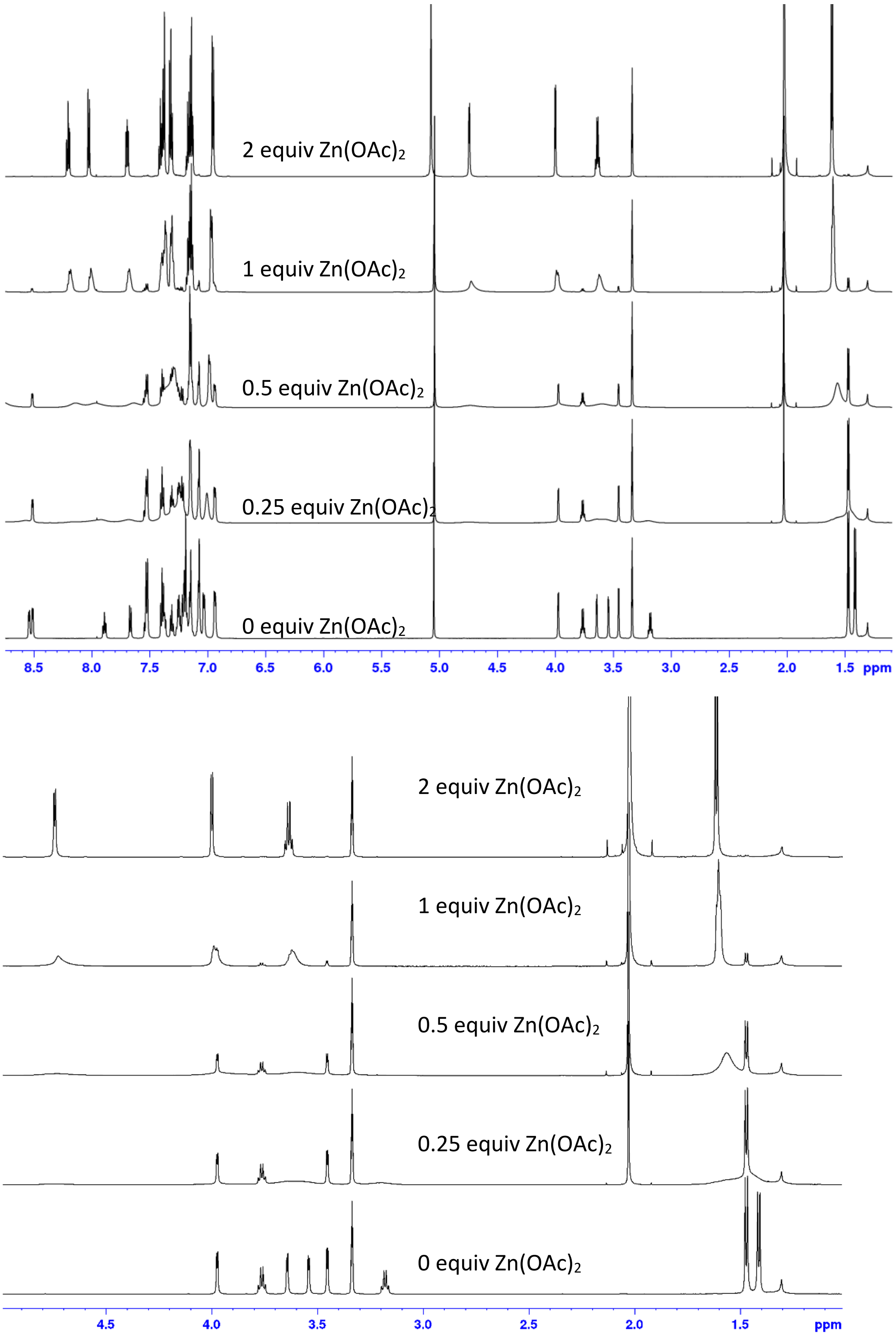

Figure S6. NMR spectra (600 MHz, CD_3OD) collected during titration of (1*S*/1*R*,2*S*,3*R*)-**10** with zinc acetate: full view (top) and expanded aliphatic region (bottom); signal intensity is maintained versus internal standard (dichloromethane).

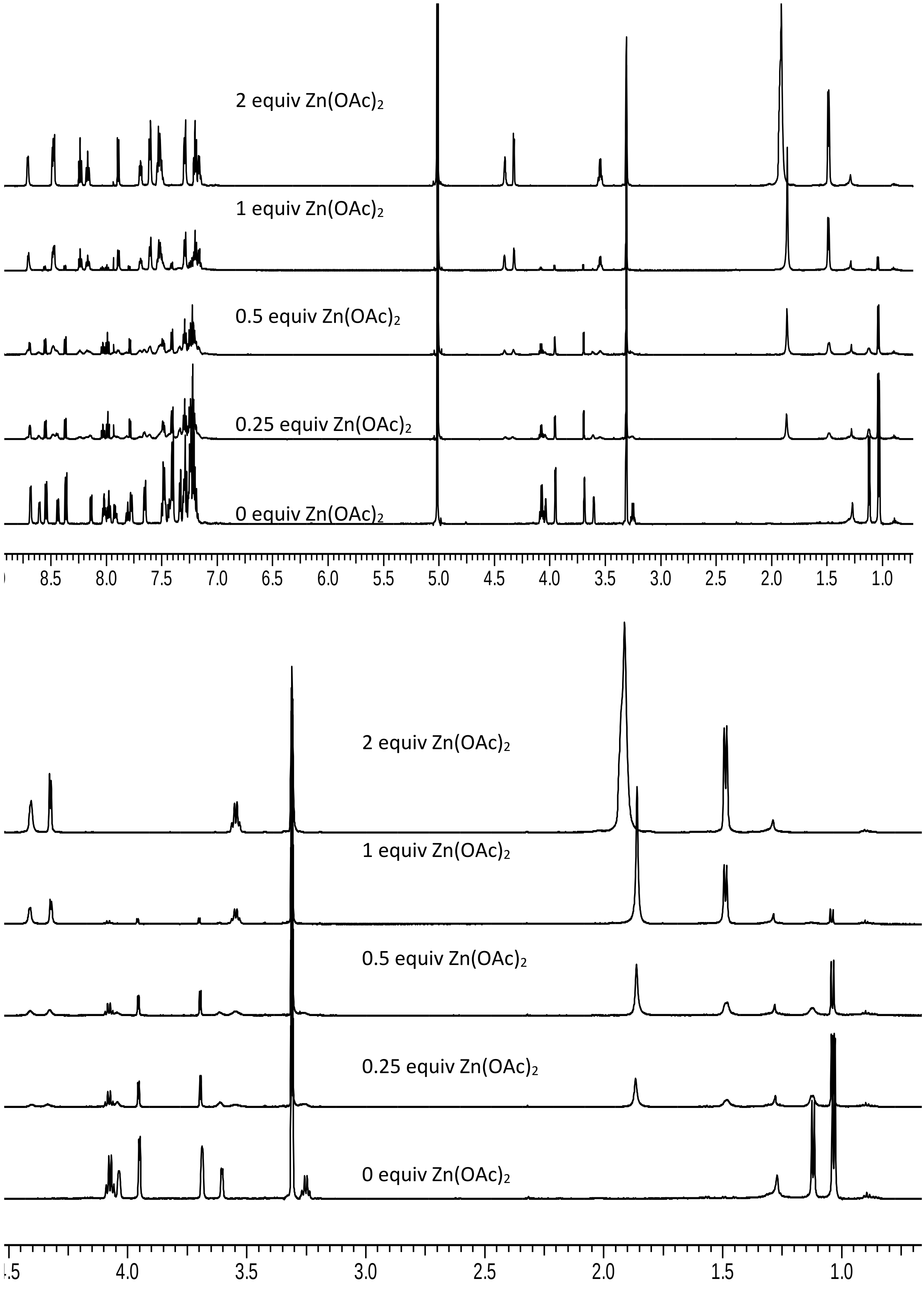

Figure S7. NMR spectra (600 MHz, CD_3OD) collected during titration of (1*S*/1*R*,2*R*,3*S*)-**12** with zinc acetate: full view (top) and expanded aliphatic region (bottom). signal intensity is maintained versus internal standard (dichloromethane).

4. EXSY experiment for (2*S*,3*R*,1'*S*)-**10** and $^{1}H,^{13}C$ HSQC and HMBC spectra for azides **3**, **13**, **14** and **19**

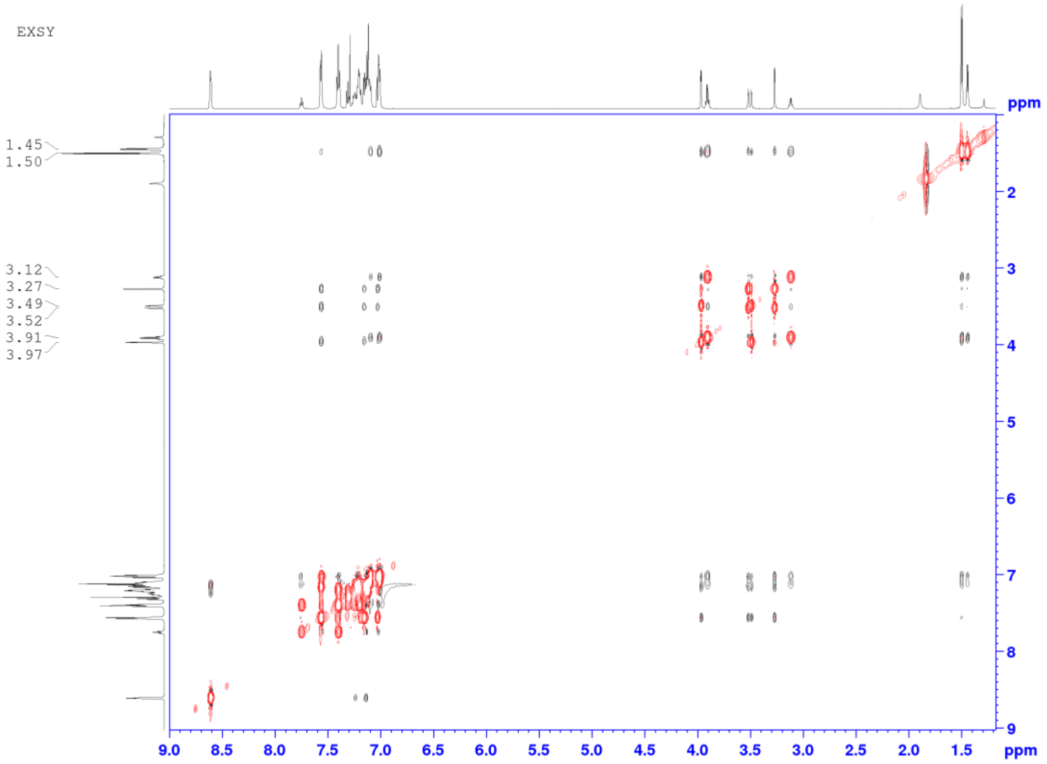

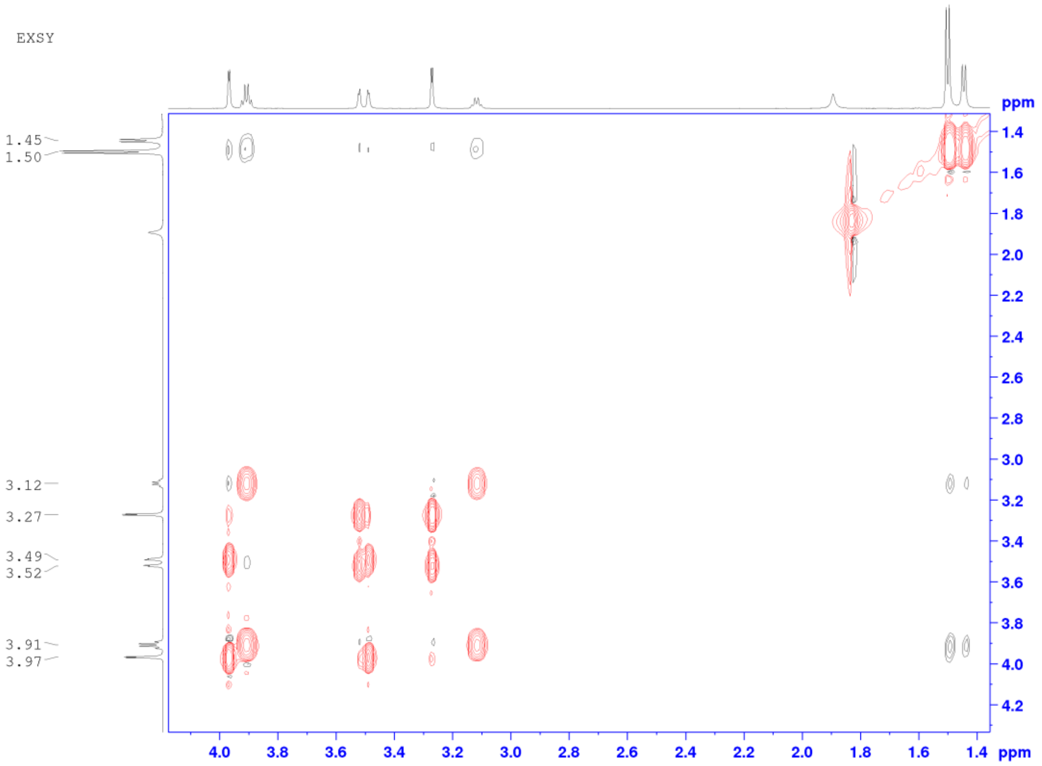

Figure S8. EXSY experiment for (2*S*,3*R*,1'*S*)-**10**, full (top) and expanded view (bottom). Positive phase correlations are drawn in red.

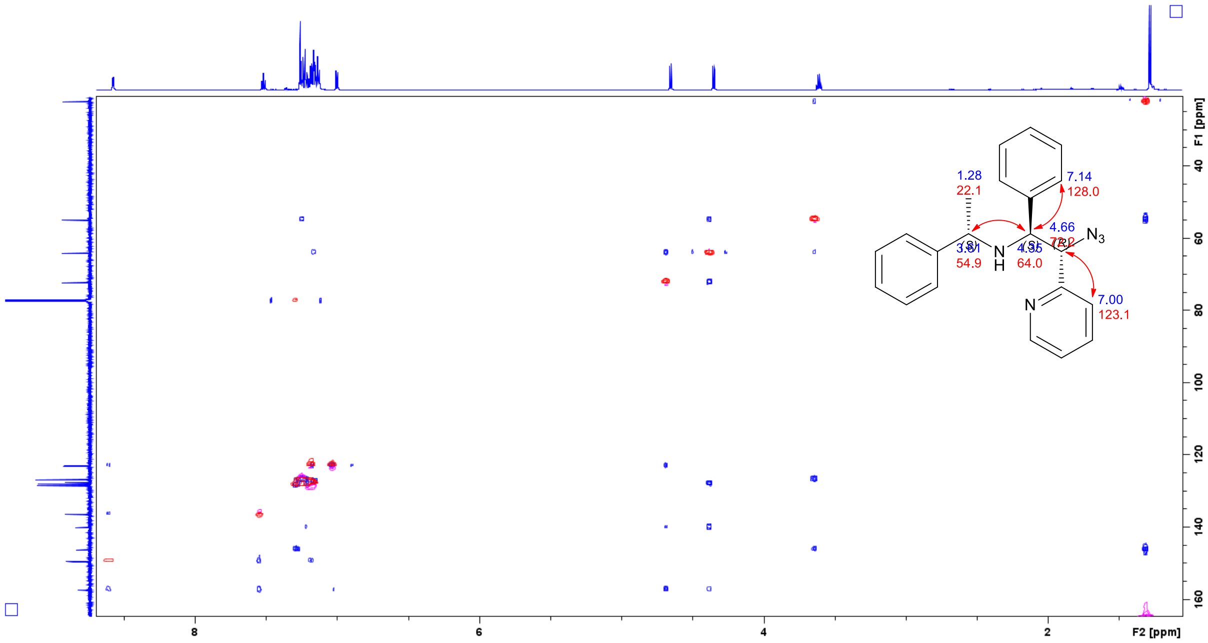

Figure S9. Overlay of $^{1}H,^{13}C$ HSQC (red) and HMBC (blue) experiments for (1*R*,2S,1'*S*)-**19** in $CDCl_3$ (600, 151 MHz). Inset structure drawing shows the assignment of selected ^{1}H NMR shifts in blue, ^{13}C NMR shifts in red, and HMBC correlations as arrows.

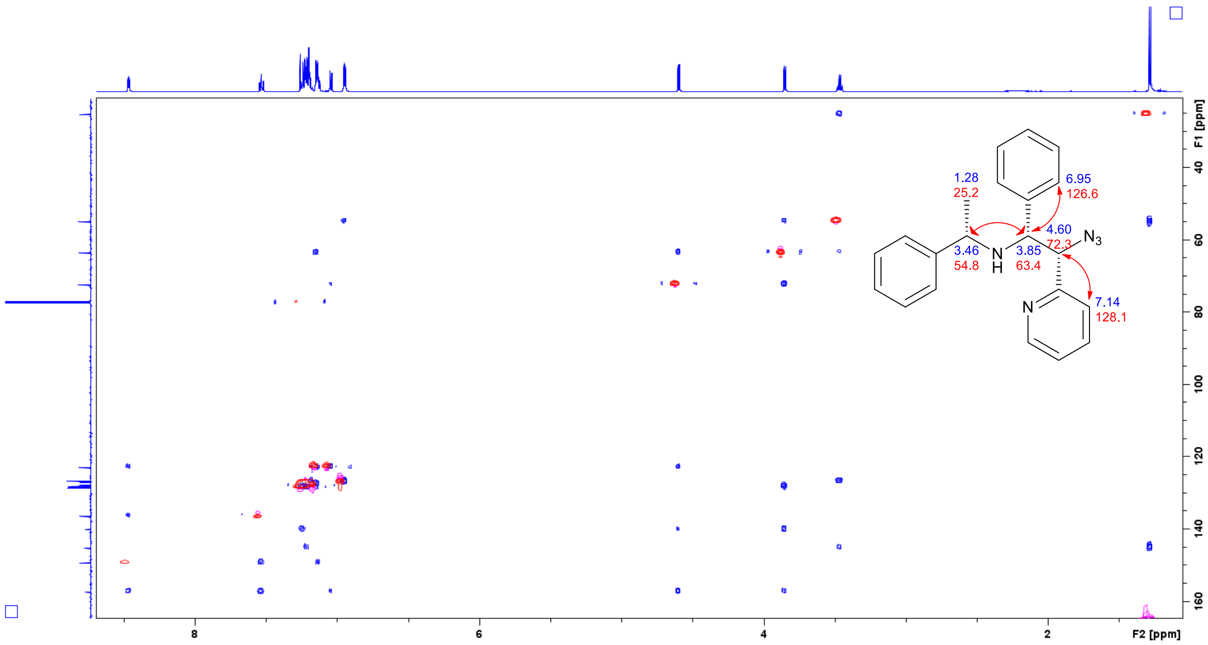

Figure S10. Overlay of $^{1}H,^{13}C$ HSQC (red) and HMBC (blue) experiments for (1*S*,2*R*,*1'S*)-**19** in $CDCl_3$ (600, 151 MHz). Inset structure drawing shows the assignment of selected ^{1}H NMR shifts in blue, ^{13}C NMR shifts in red, and HMBC correlations as arrows.

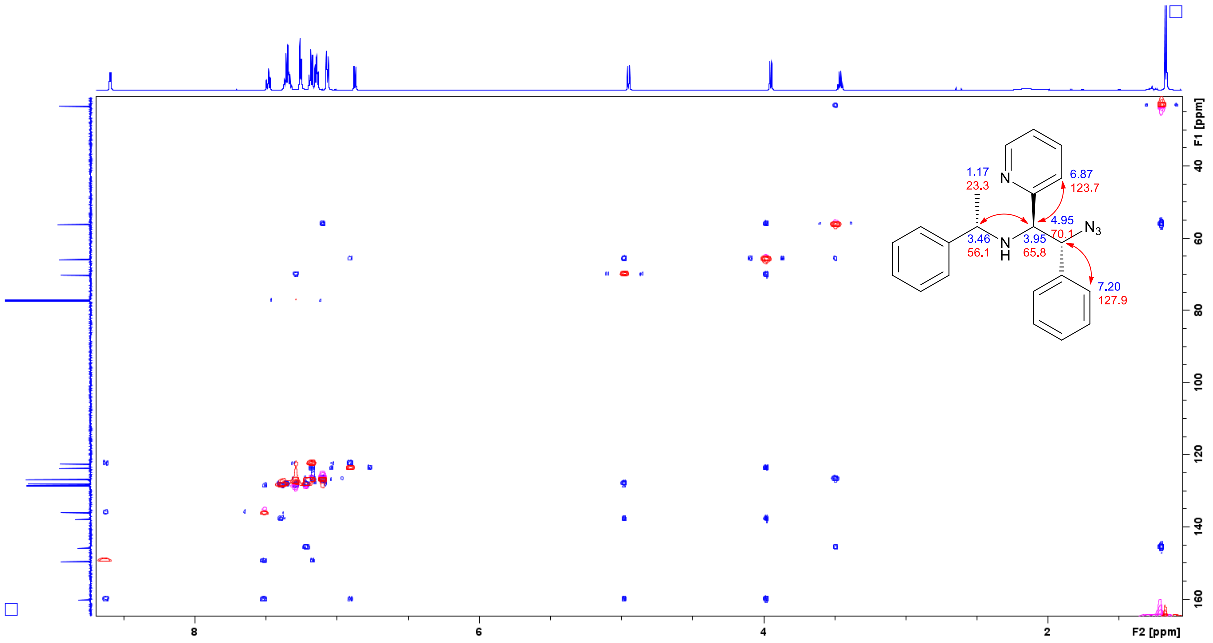

Figure S11. Overlay of ^{1}H,^{13}C HSQC (red) and HMBC (blue) experiments for (1*R*,2*R*,1'*S*)-**13** in $CDCl_3$ (600, 151 MHz). Inset structure drawing shows the assignment of selected ^{1}H NMR shifts in blue, ^{13}C NMR shifts in red, and HMBC correlations as arrows.

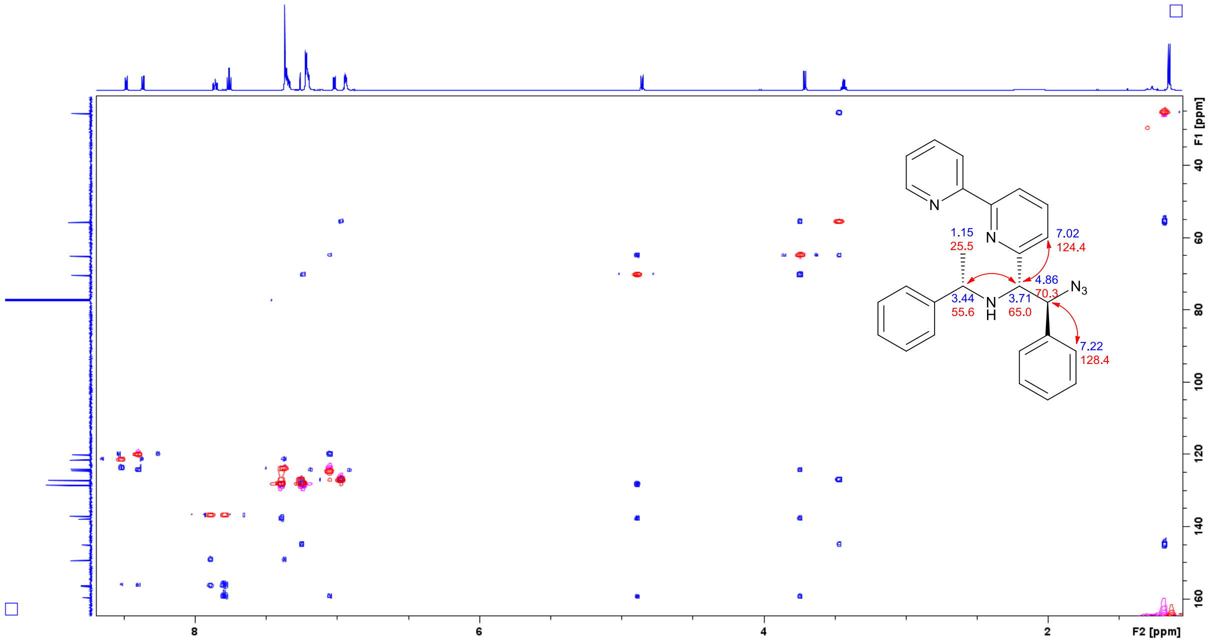

Figure S12. Overlay of ^{1}H,^{13}C HSQC (red) and HMBC (blue) experiments for (1*S*,2*S*,1'*S*)-**14** in $CDCl_3$ (600, 151 MHz). Inset structure drawing shows the assignment of selected ^{1}H NMR shifts in blue, ^{13}C NMR shifts in red, and HMBC correlations as arrows.

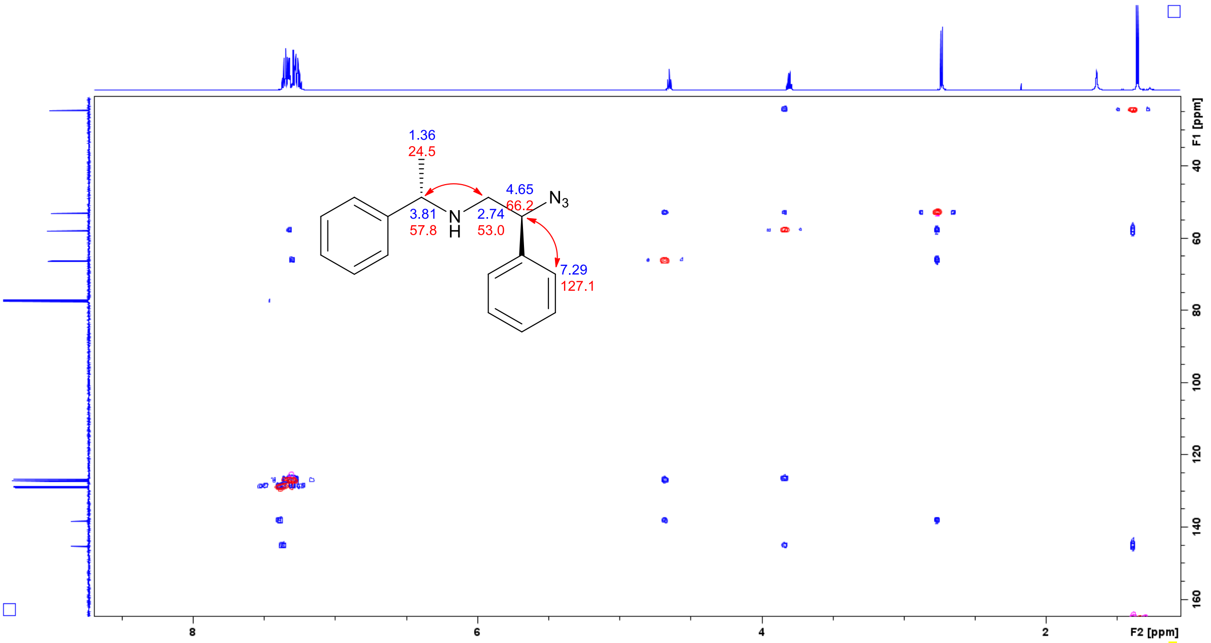

Figure S13. Overlay of ^{1}H,^{13}C HSQC (red) and HMBC (blue) experiments for (1*R*,1'*S*)-**3** in CDCl$_3$ (600, 151 MHz). Inset structure drawing shows the assignment of selected ^{1}H NMR shifts in blue, ^{13}C NMR shifts in red, and HMBC correlations as arrows.

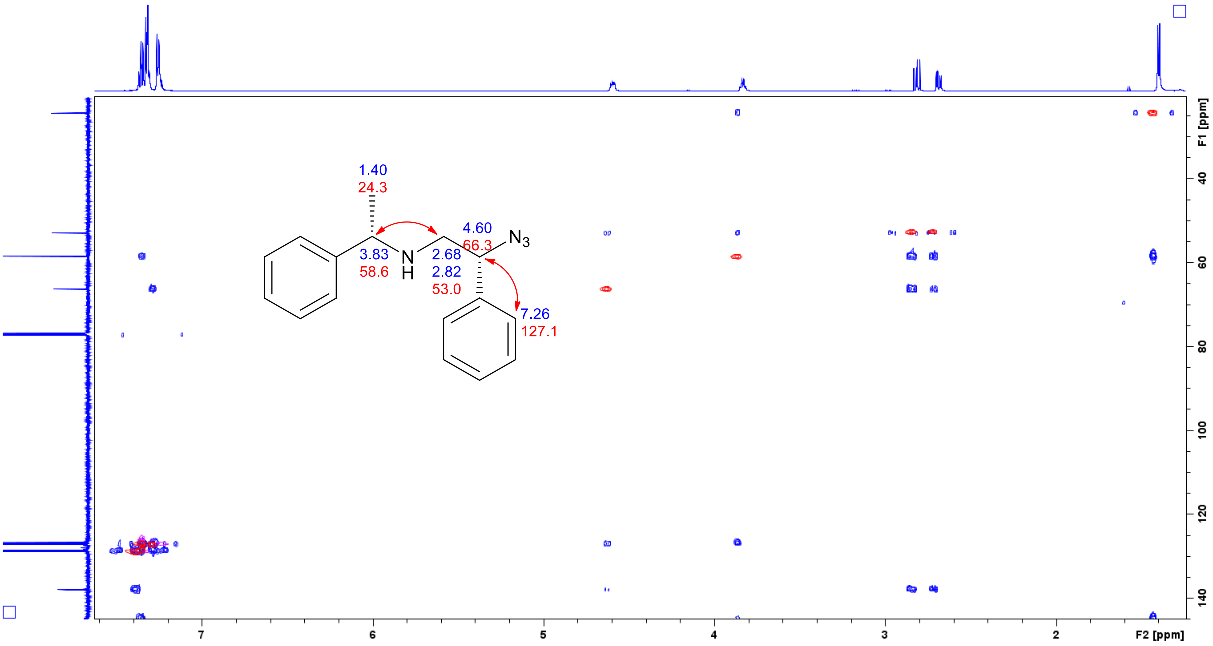

Figure S14. Overlay of ^{1}H,^{13}C HSQC (red) and HMBC (blue) experiments for (1*S*,1'*S*)-**3** in CDCl$_3$ (600, 151 MHz). Inset structure drawing shows the assignment of selected ^{1}H NMR shifts in blue, ^{13}C NMR shifts in red, and HMBC correlations as arrows.

5. Copies of NMR Spectra

This report was created by ACD/NMR Processor Academic Edition. For more information go to www.acdlabs.com/nmrproc/

Acquisition Time (sec)	3.2716	Comment	single_pulse	Date	05 Dec 2018 08:31:57		
Date Stamp	05 Dec 2018 08:30:08			File Name	C:\Users\Marcin\Documents\widma NMR\MW-210-119-6_Proton-1-1.jdf		
Frequency (MHz)	399.78	Nucleus	1H	Number of Transients	16	Origin	ECA
Original Points Count	32768	Owner	Delta	Points Count	32768	Pulse Sequence	proton.jxp
Solvent	CHLOROFORM-d			Spectrum Offset (Hz)	2398.6931	Sweep Width (Hz)	10016.03
Temperature (degree C)	21.700						

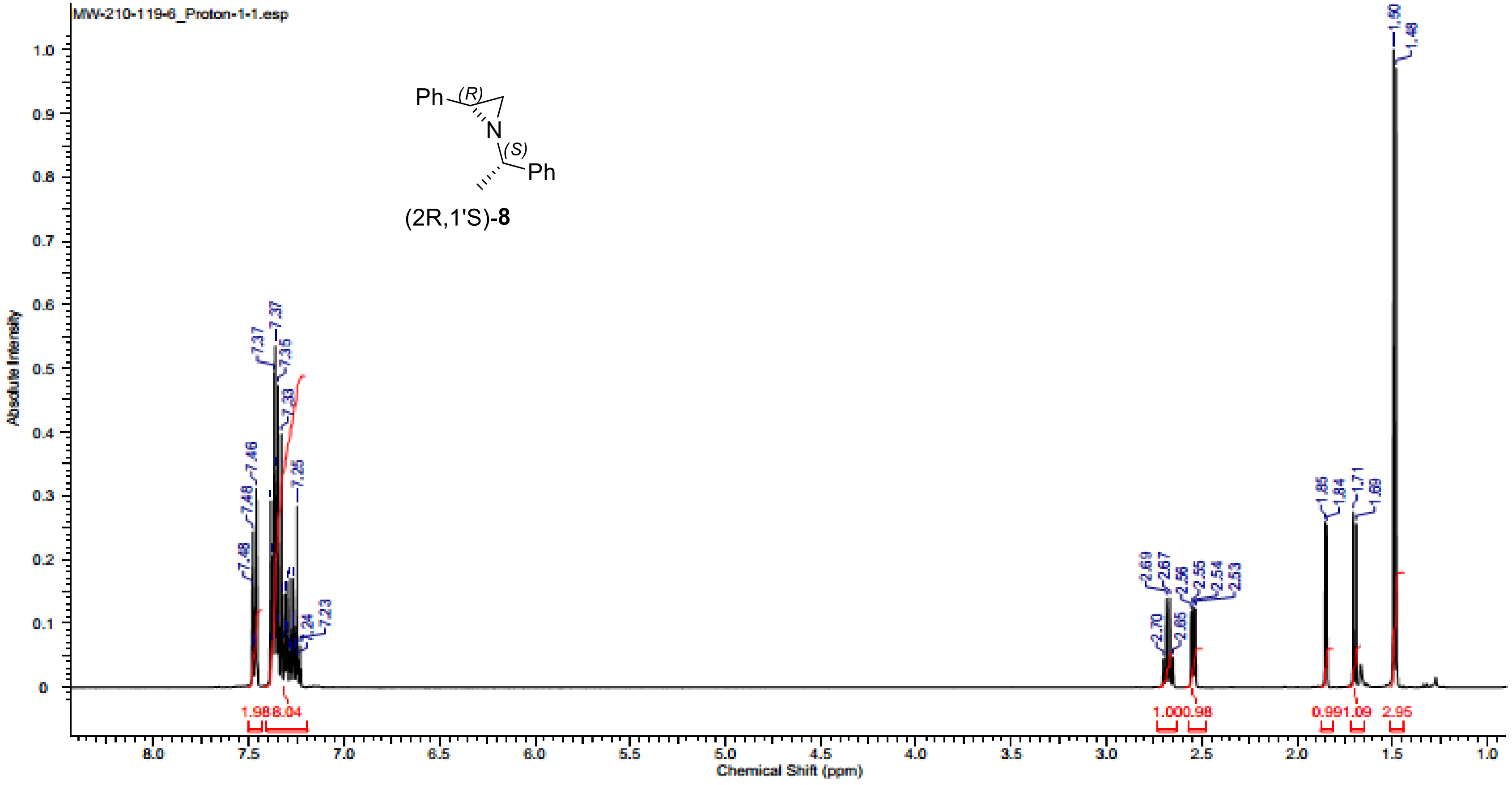

C:\Users\Marcin\Documents\widma NMR\MW-210-119-6_Proton-1-1.esp

Figure S15. ^{1}H NMR spectrum (400 MHz, $CDCl_3$) for (2*R*,1'*S*)-**8**

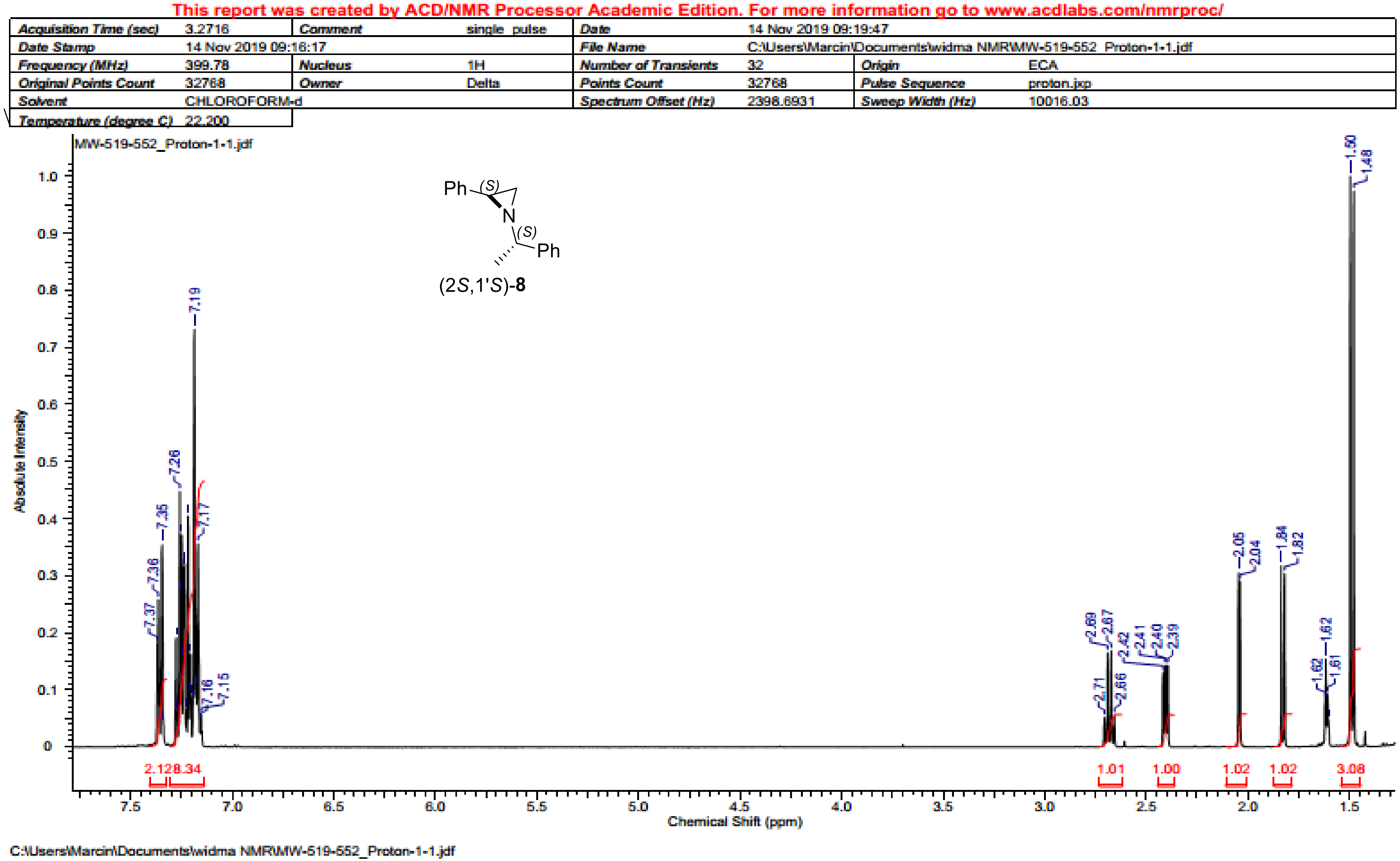

Figure S16. ^{1}H NMR spectrum (400 MHz, $CDCl_3$) for (2*S*,1'*S*)-**8**

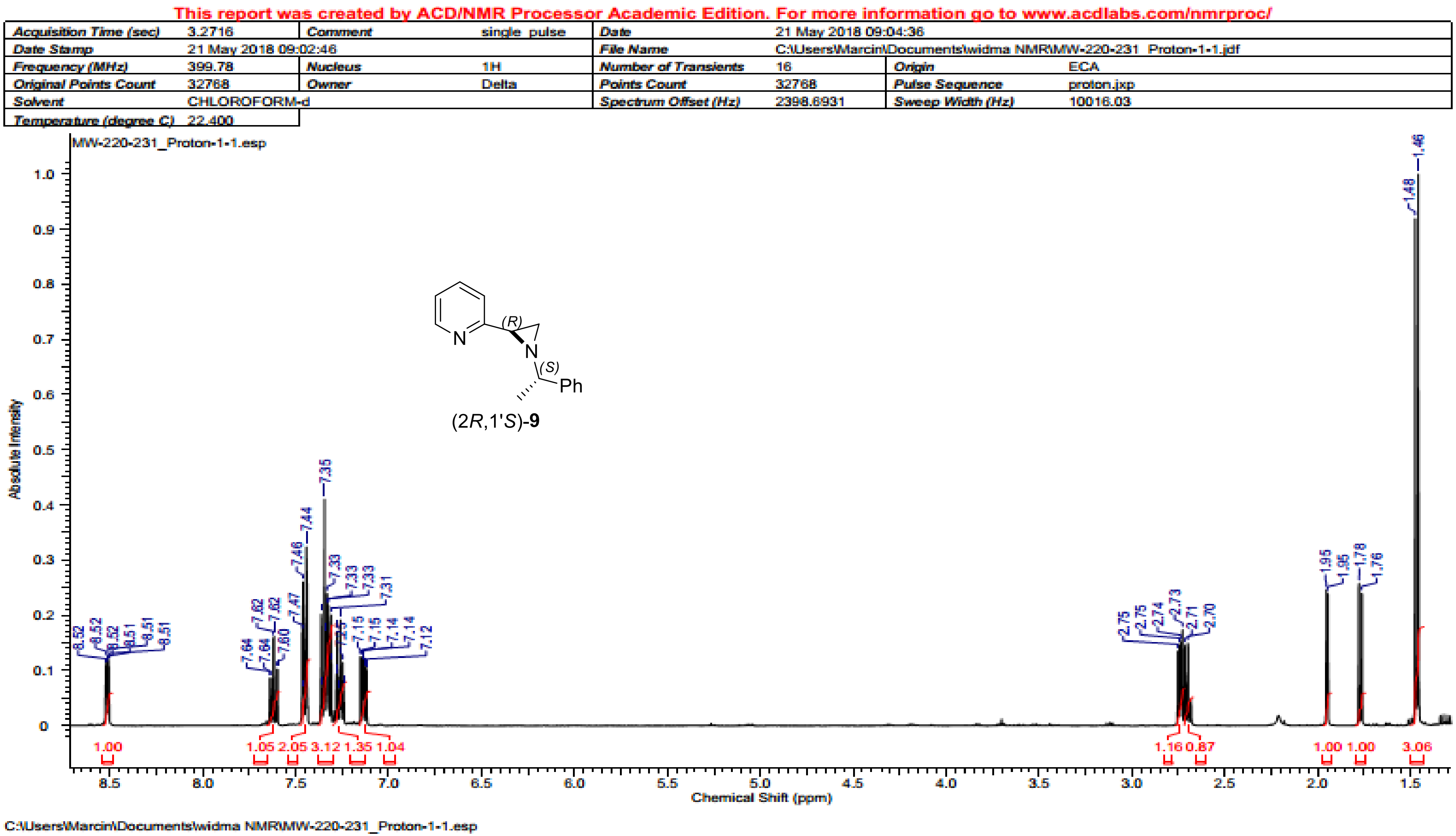

Figure S17. ^{1}H NMR spectrum (400 MHz, $CDCl_3$) for (2*R*,1'*S*)-**9**

This report was created by ACD/NMR Processor Academic Edition. For more information go to www.acdlabs.com/nmrproc/

Acquisition Time (sec)	1.7406	*Comment*	single pulse decoupled gated NOE			*Date*	23 May 2018 08:36:25
Date Stamp	23 May 2018 08:20:08			*File Name*	C:\Users\Marcin\Documents\widma NMR\MW-220-231_carbon-1-1.jdf		
Frequency (MHz)	100.53	*Nucleus*	13C	*Number of Transients*	338	*Origin*	ECA
Original Points Count	65536	*Owner*	Delta	*Points Count*	65536	*Pulse Sequence*	carbon.jxp
Solvent	CHLOROFORM-d			*Spectrum Offset (Hz)*	10052.5303	*Sweep Width (Hz)*	37650.60
Temperature (degree C)	22.300						

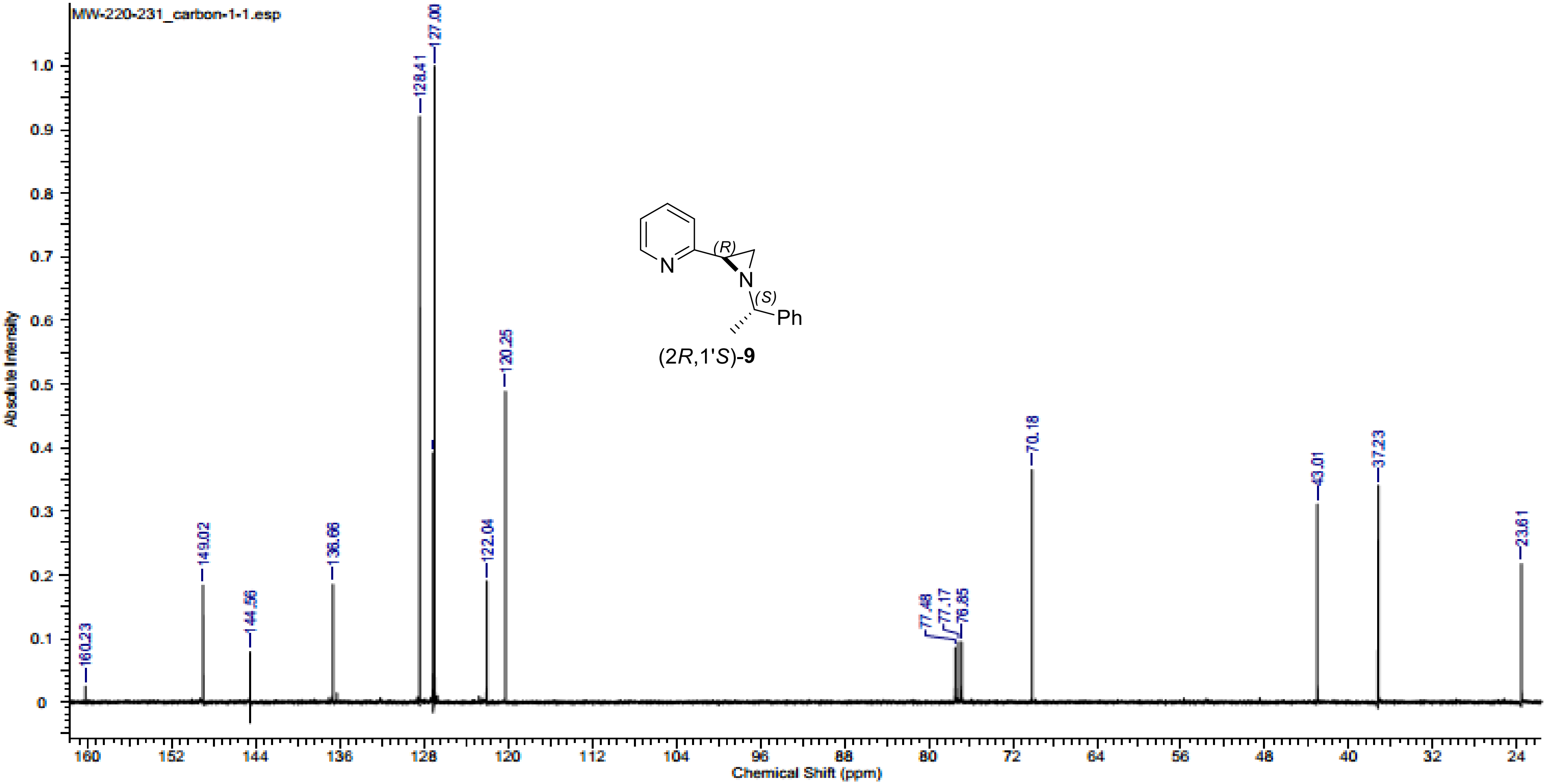

C:\Users\Marcin\Documents\widma NMR\MW-220-231_carbon-1-1.esp

Figure S18. ^{13}C NMR spectrum (101 MHz, $CDCl_3$) for (2*R*,1'*S*)-**8**

This report was created by ACD/NMR Processor Academic Edition. For more information go to www.acdlabs.com/nmrproc/

Acquisition Time (sec)	2.7263	Comment	5 mm PABBO BB-1H/D Z-GRD Z847801/0325		Date	13 Nov 2019 10:16:48
Date Stamp	13 Nov 2019 10:16:48		File Name	C:\Users\Marcin\Documents\widma NMR\MW-507-535-nt-283K\1\fid		
Frequency (MHz)	600.58	Nucleus	1H	Number of Transients 32	Origin	spect
Original Points Count	32768	Owner	nmrsu	Points Count 32768	Pulse Sequence	zg30
Receiver Gain	114.00	SW(cyclical) (Hz)	12019.23	Solvent CHLOROFORM-d		
Spectrum Offset (Hz)	3708.5806	Spectrum Type	STANDARD	Sweep Width (Hz) 12018.86	Temperature (degree C)	10.000

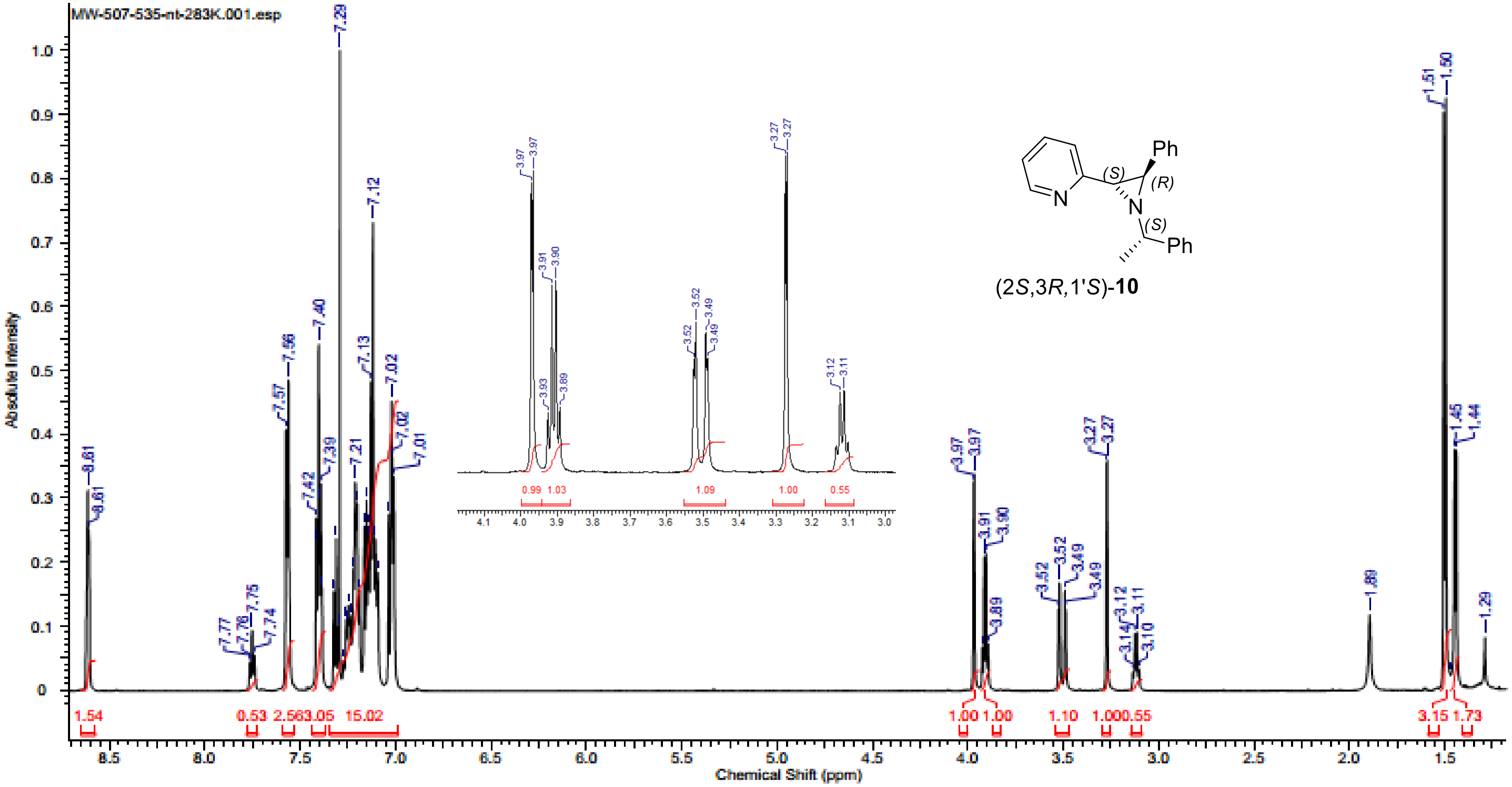

C:\Users\Marcin\Documents\widma NMR\MW-507-535-nt-283K\MW-507-535-nt-283K.001.esp

Figure S19. ^{1}H NMR spectrum (283K, 600 MHz, $CDCl_3$) for (2*S*,3*R*,1'*S*)-**10**

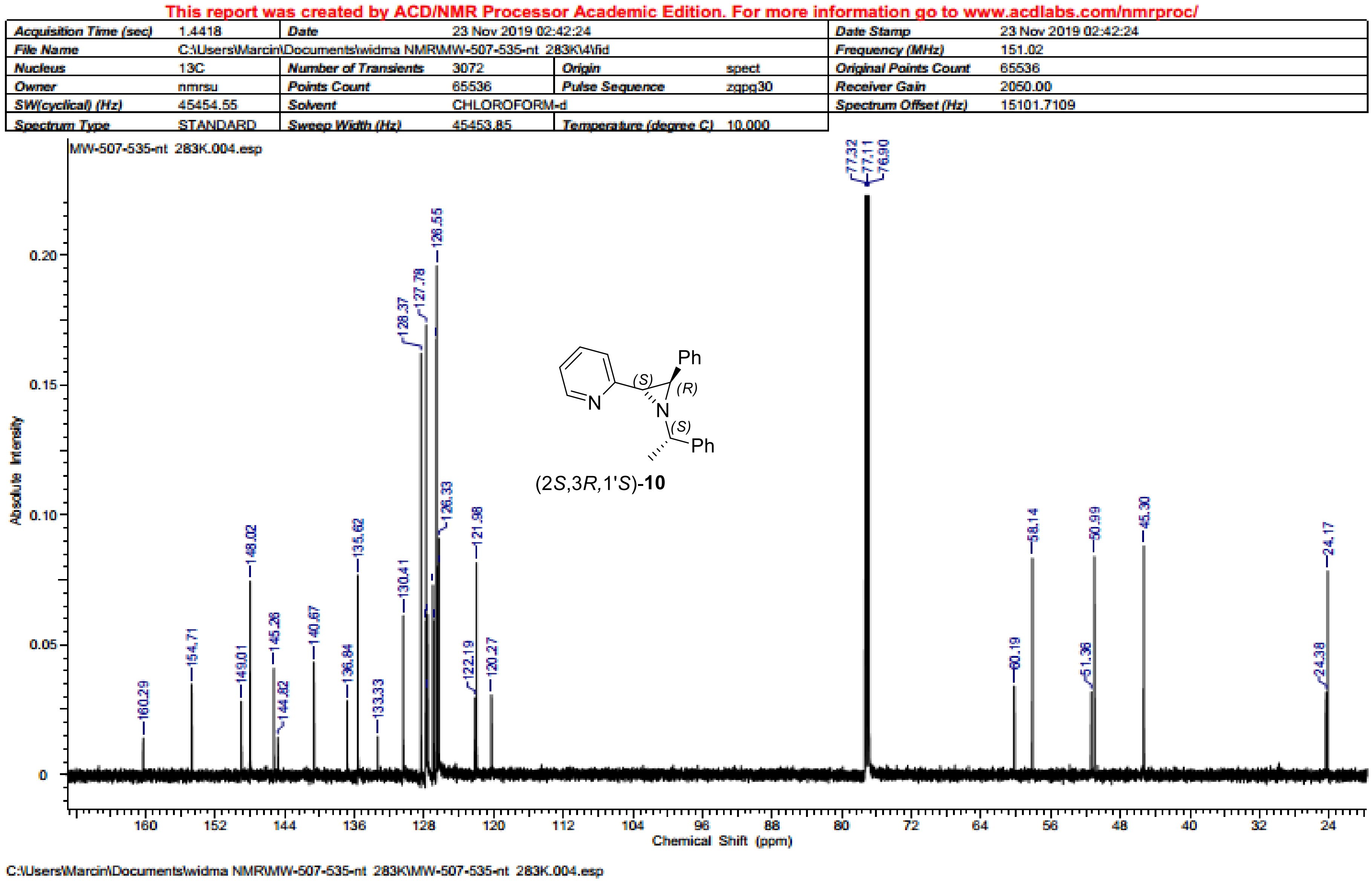

Figure S20. ^{13}C NMR spectrum (283K, 151 MHz, $CDCl_3$) for (2*S*,3*R*,1'*S*)-**10**

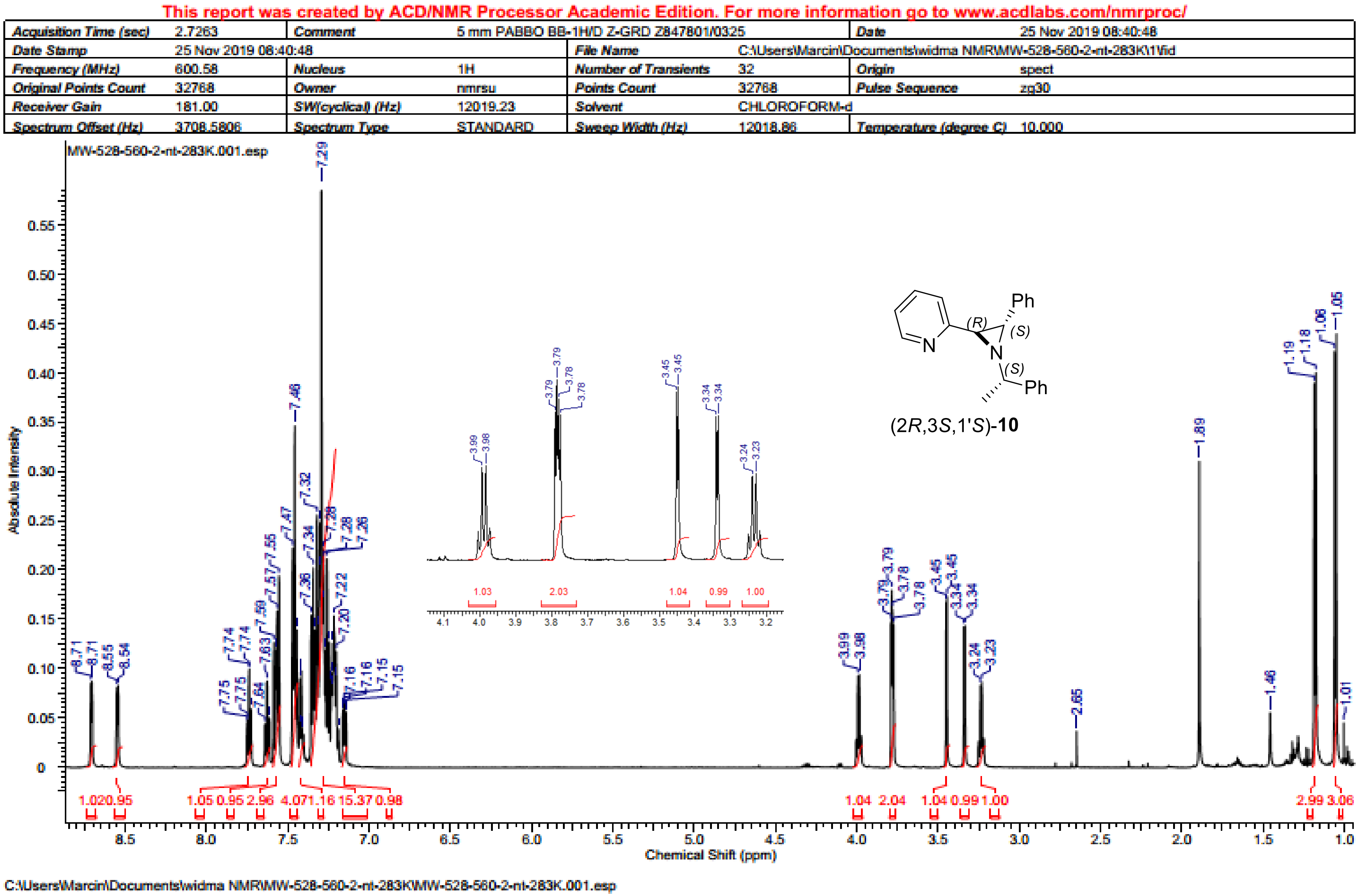

Figure S21. ^{1}H NMR spectrum (283K, 600 MHz, $CDCl_3$) for (2*R*,3*S*,1'*S*)-**10**

This report was created by ACD/NMR Processor Academic Edition. For more information go to www.acdlabs.com/nmrproc/

Acquisition Time (sec)	1.4418	Date	25 Nov 2019 09:46:56			Date Stamp	25 Nov 2019 09:46:56
File Name	C:\Users\Marcin\Documents\widma NMR\MW-528-560-2-nt-283K\2\fid					Frequency (MHz)	151.02
Nucleus	13C	Number of Transients	2002	Origin	spect	Original Points Count	65536
Owner	nmrsu	Points Count	65536	Pulse Sequence	zgpg30	Receiver Gain	2050.00
SW(cyclical) (Hz)	45454.55	Solvent	CHLOROFORM-d			Spectrum Offset (Hz)	15101.7109
Spectrum Type	STANDARD	Sweep Width (Hz)	45453.85	Temperature (degree C)	10.000		

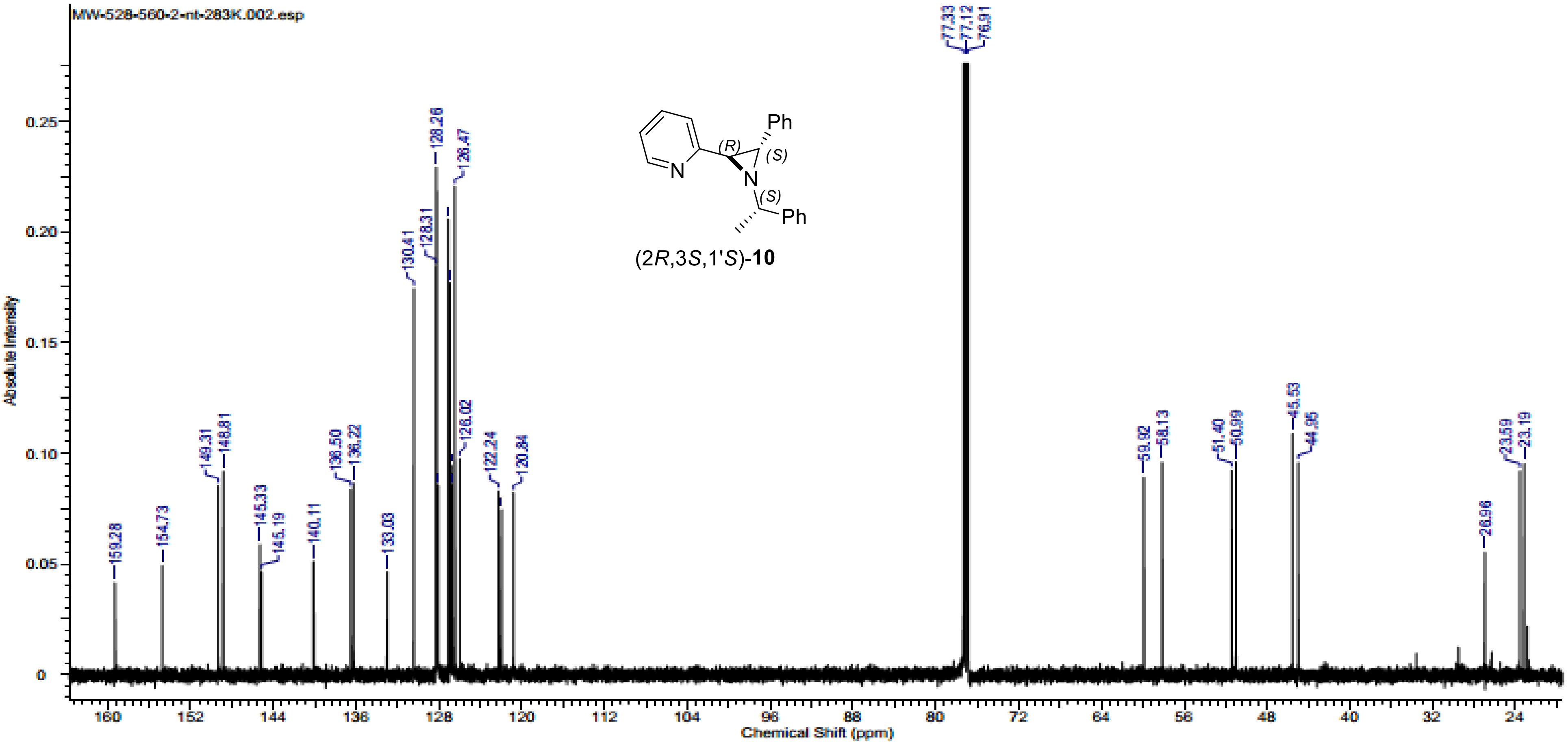

C:\Users\Marcin\Documents\widma NMR\MW-528-560-2-nt-283K\MW-528-560-2-nt-283K.002.esp

Figure S22. ^{13}C NMR spectrum (283K, 151 MHz, $CDCl_3$) for (2*R*,3*S*,1'*S*)-**10**

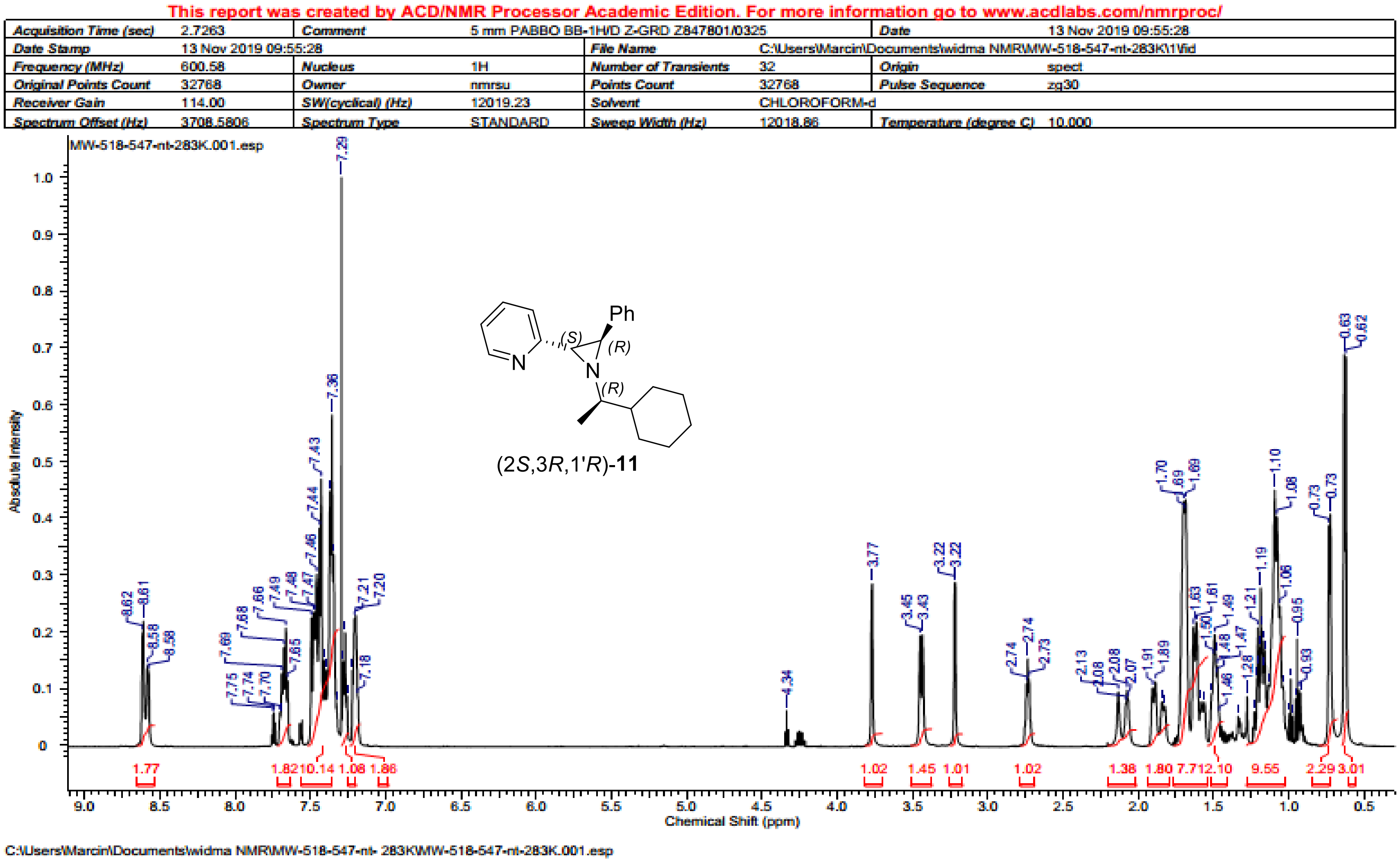

Figure S23. ^{1}H NMR spectrum (283K, 600 MHz, $CDCl_3$) for (2*S*,3*R*,1'*R*)-**11**

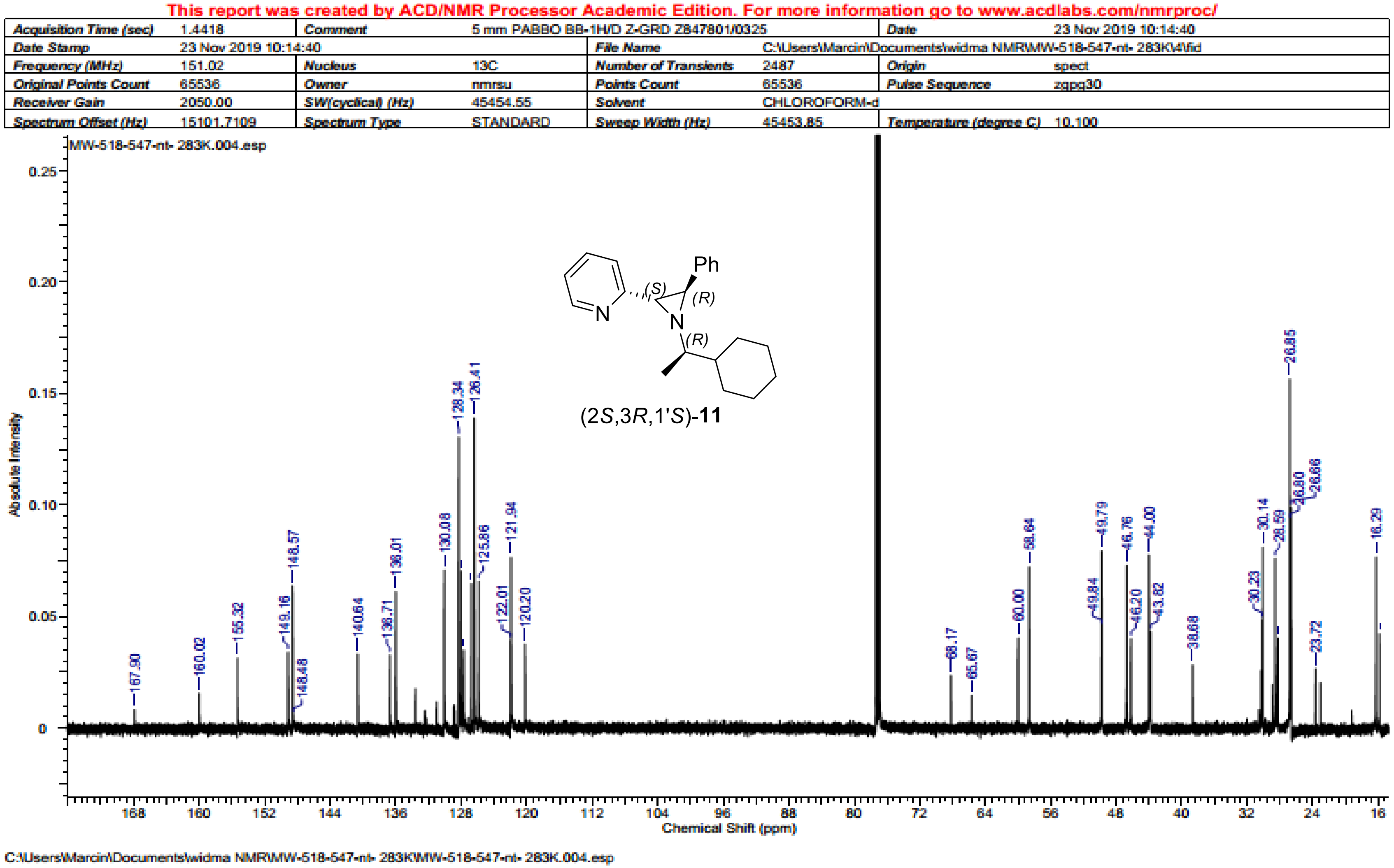

Figure S24. ^{13}C NMR spectrum (283K, 151 MHz, $CDCl_3$) for (2*S*,3*R*,1'*R*)-**11**

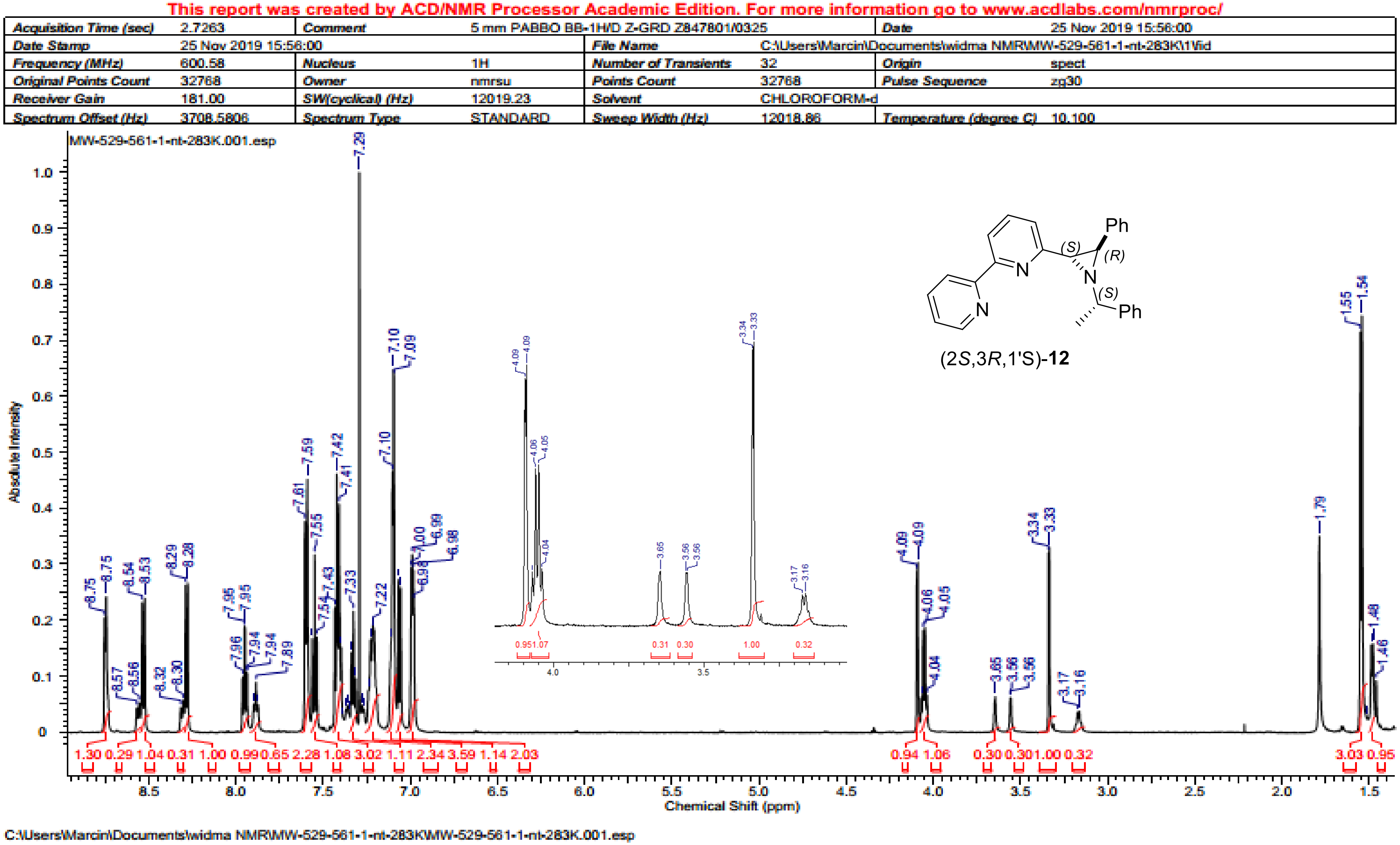

Figure S25. ^{1}H NMR spectrum (283K, 600 MHz, $CDCl_3$) for (2*S*,3*R*,1'*S*)-**12**

This report was created by ACD/NMR Processor Academic Edition. For more information go to www.acdlabs.com/nmrproc/

Acquisition Time (sec)	1.4418	*Date*	25 Nov 2019 16:10:56			*Date Stamp*	25 Nov 2019 16:10:56
File Name	C:\Users\Marcin\Documents\widma NMR\MW-529-561-1-nt-283K\2\fid					*Frequency (MHz)*	151.02
Nucleus	13C	*Number of Transients*	2786	*Origin*	spect	*Original Points Count*	65536
Owner	nmrsu	*Points Count*	65536	*Pulse Sequence*	zgpg30	*Receiver Gain*	2050.00
SW(cyclical) (Hz)	45454.55	*Solvent*	CHLOROFORM-d			*Spectrum Offset (Hz)*	15101.7109
Spectrum Type	STANDARD	*Sweep Width (Hz)*	45453.85	*Temperature (degree C)*	10.200		

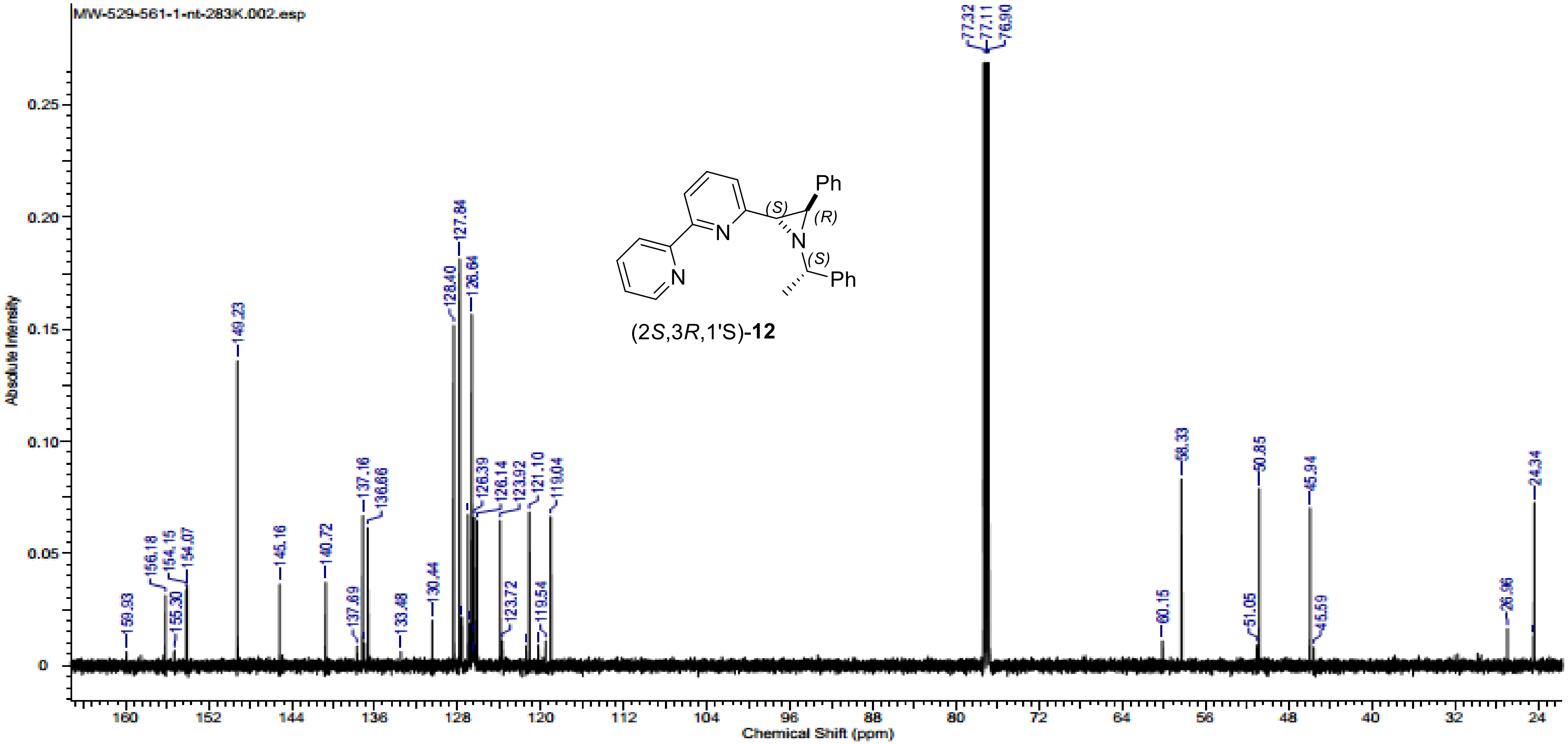

C:\Users\Marcin\Documents\widma NMR\MW-529-561-1-nt-283K\MW-529-561-1-nt-283K.002.esp

Figure S26. ^{13}C NMR spectrum (283K, 151 MHz, $CDCl_3$) for (2*S*,3*R*,1'*S*)-**12**

This report was created by ACD/NMR Processor Academic Edition. For more information go to www.acdlabs.com/nmrproc/

Acquisition Time (sec)	2.7263	Comment	5 mm PABBO BB-1H/D Z-GRD Z847801/0325			Date	25 Nov 2019 10:53:04
Date Stamp	25 Nov 2019 10:53:04			File Name	C:\Users\Marcin\Documents\widma NMR\MW-530-562-1-nt-283K\1\fid		
Frequency (MHz)	600.58	Nucleus	1H	Number of Transients	32	Origin	spect
Original Points Count	32768	Owner	nmrsu	Points Count	32768	Pulse Sequence	zg30
Receiver Gain	181.00	SW(cyclical) (Hz)	12019.23	Solvent	CHLOROFORM-d		
Spectrum Offset (Hz)	3708.5806	Spectrum Type	STANDARD	Sweep Width (Hz)	12018.86	Temperature (degree C)	10.000

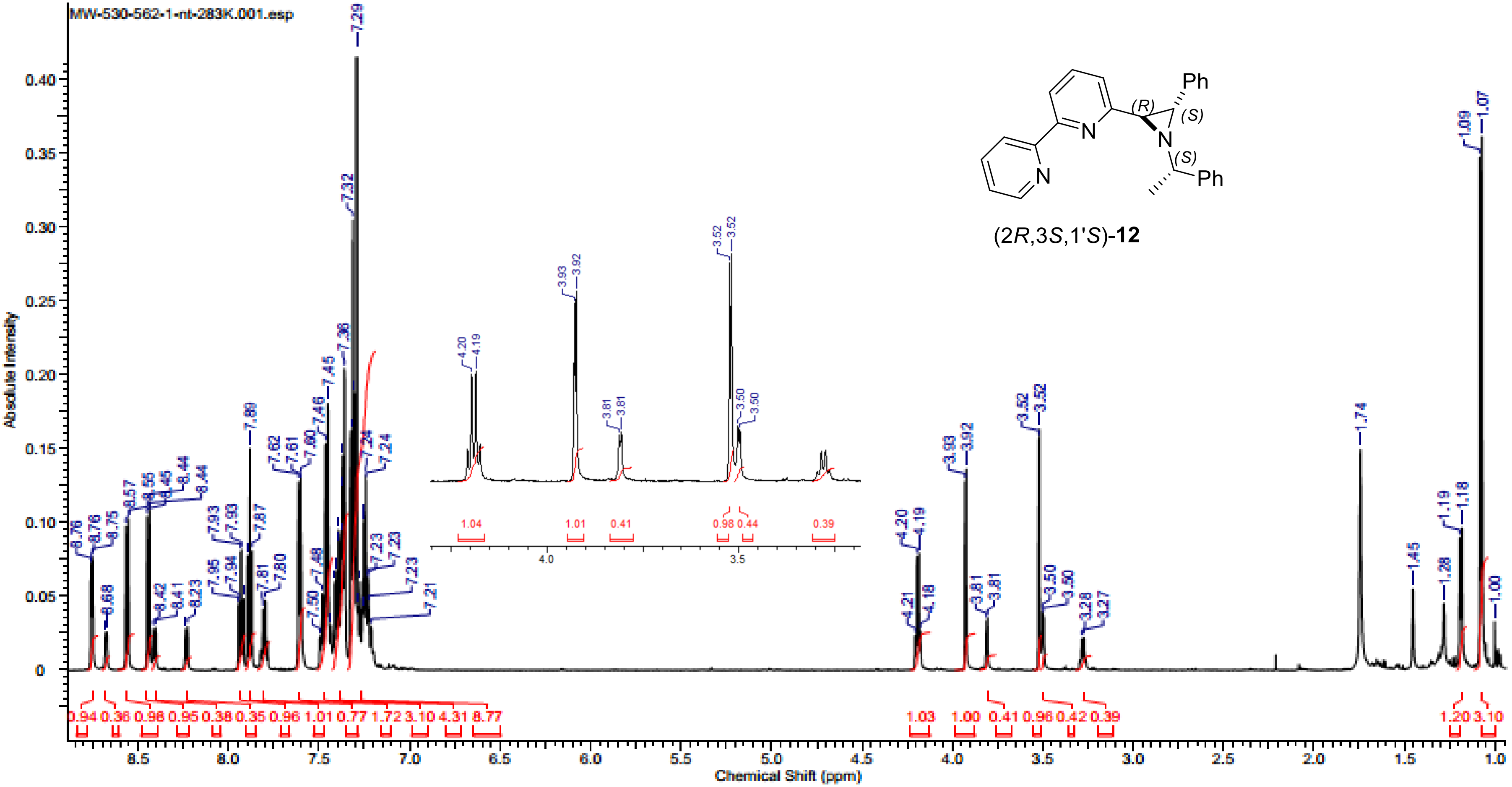

C:\Users\Marcin\Documents\widma NMR\MW-530-562-1-nt-283K\MW-530-562-1-nt-283K.001.esp

Figure S27. ^{1}H NMR spectrum (283K, 600 MHz, $CDCl_3$) for (2*R*,3*S*,1'*S*)-**12**

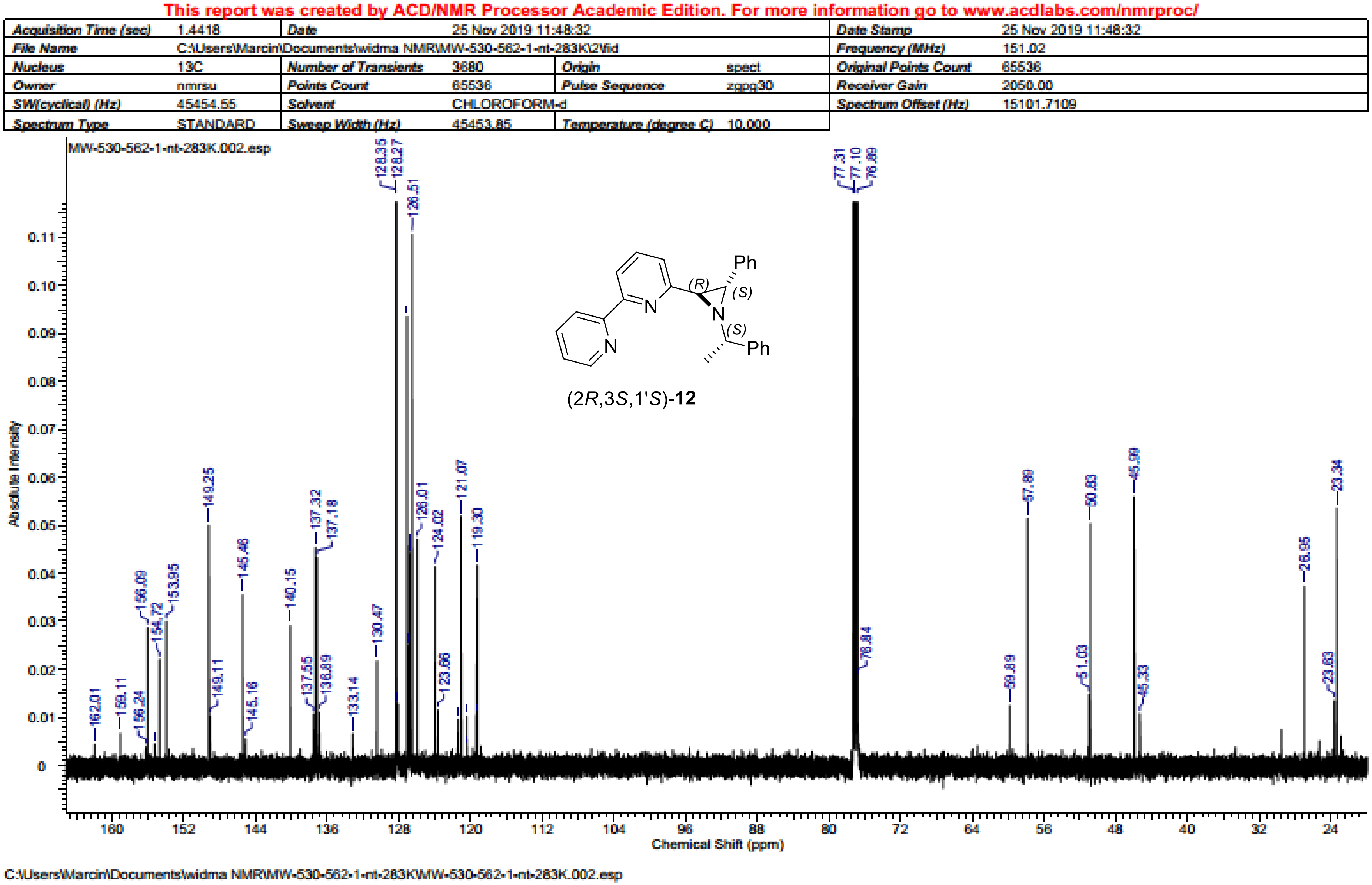

Figure S28. ^{13}C NMR spectrum (283K, 151 MHz, $CDCl_3$) for (2*R*,3*S*,1'*S*)-**12**

This report was created by ACD/NMR Processor Academic Edition. For more information go to www.acdlabs.com/nmrproc/

Acquisition Time (sec)	2.7263	*Comment*	MBA-NH-CH(Ph)-CH2-N3			*Date*	28 Nov 2019 16:47:12
Date Stamp	28 Nov 2019 16:47:12			*File Name*	C:\Users\Marcin\Documents\widma NMR\MW-536-573\1\fid		
Frequency (MHz)	600.58	*Nucleus*	1H	*Number of Transients*	32	*Origin*	spect
Original Points Count	32768	*Owner*	nmrsu	*Points Count*	32768	*Pulse Sequence*	zg30
Receiver Gain	203.00	*SW(cyclical) (Hz)*	12019.23	*Solvent*	CHLOROFORM-d		
Spectrum Offset (Hz)	3690.0164	*Spectrum Type*	STANDARD	*Sweep Width (Hz)*	12018.86	*Temperature (degree C)*	25.100

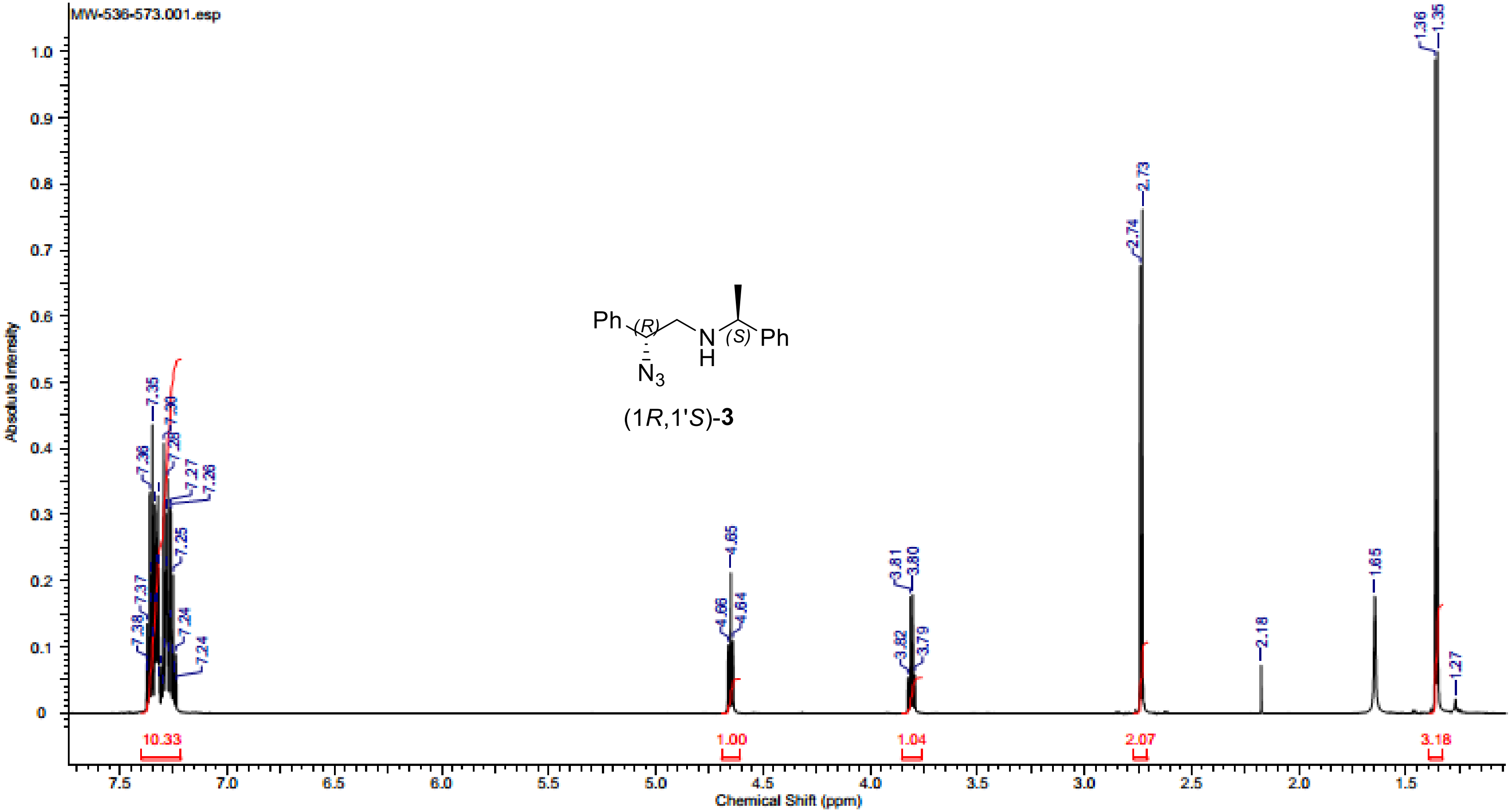

C:\Users\Marcin\Documents\widma NMR\MW-536-573\MW-536-573.001.esp

Figure S29. ^{1}H NMR spectrum (600 MHz, $CDCl_3$) for (1*R*,1'*S*)-**3**

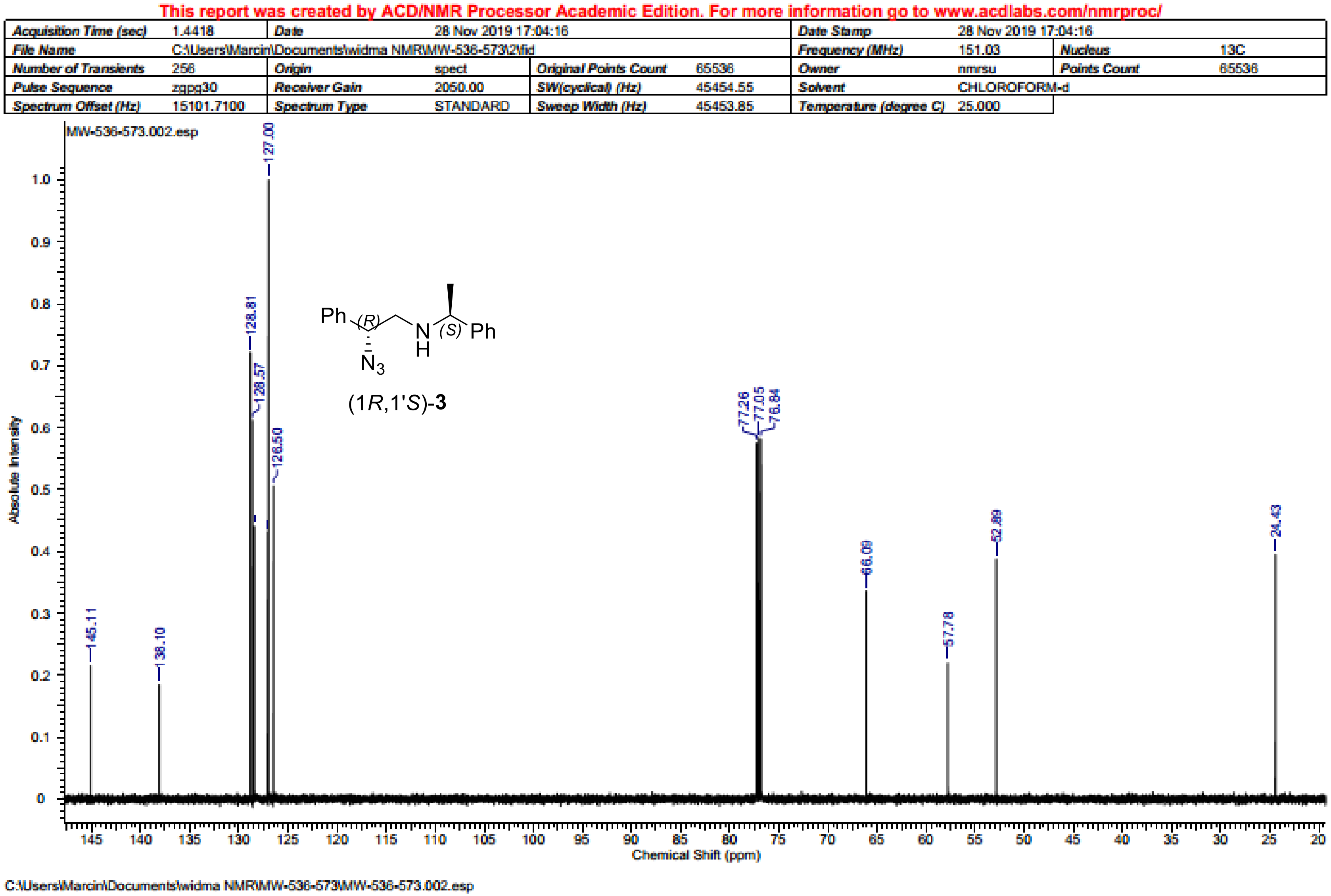

Figure S30. ^{13}C NMR spectrum (151 MHz, $CDCl_3$) for (1*R*,1'*S*)-**3**

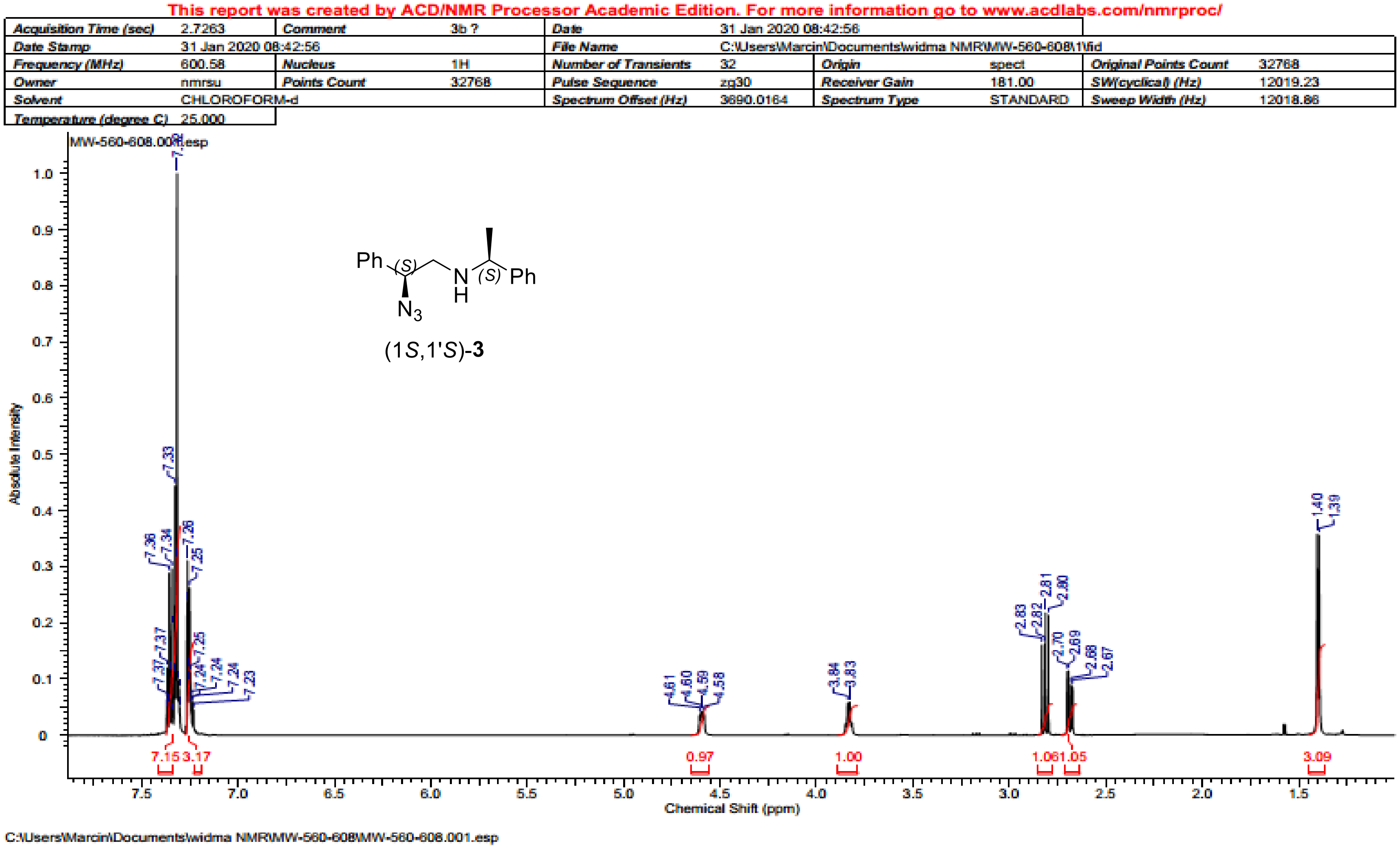

Figure S31. ^{1}H NMR spectrum (600 MHz, $CDCl_3$) for (1*S*,1'*S*)-**3**

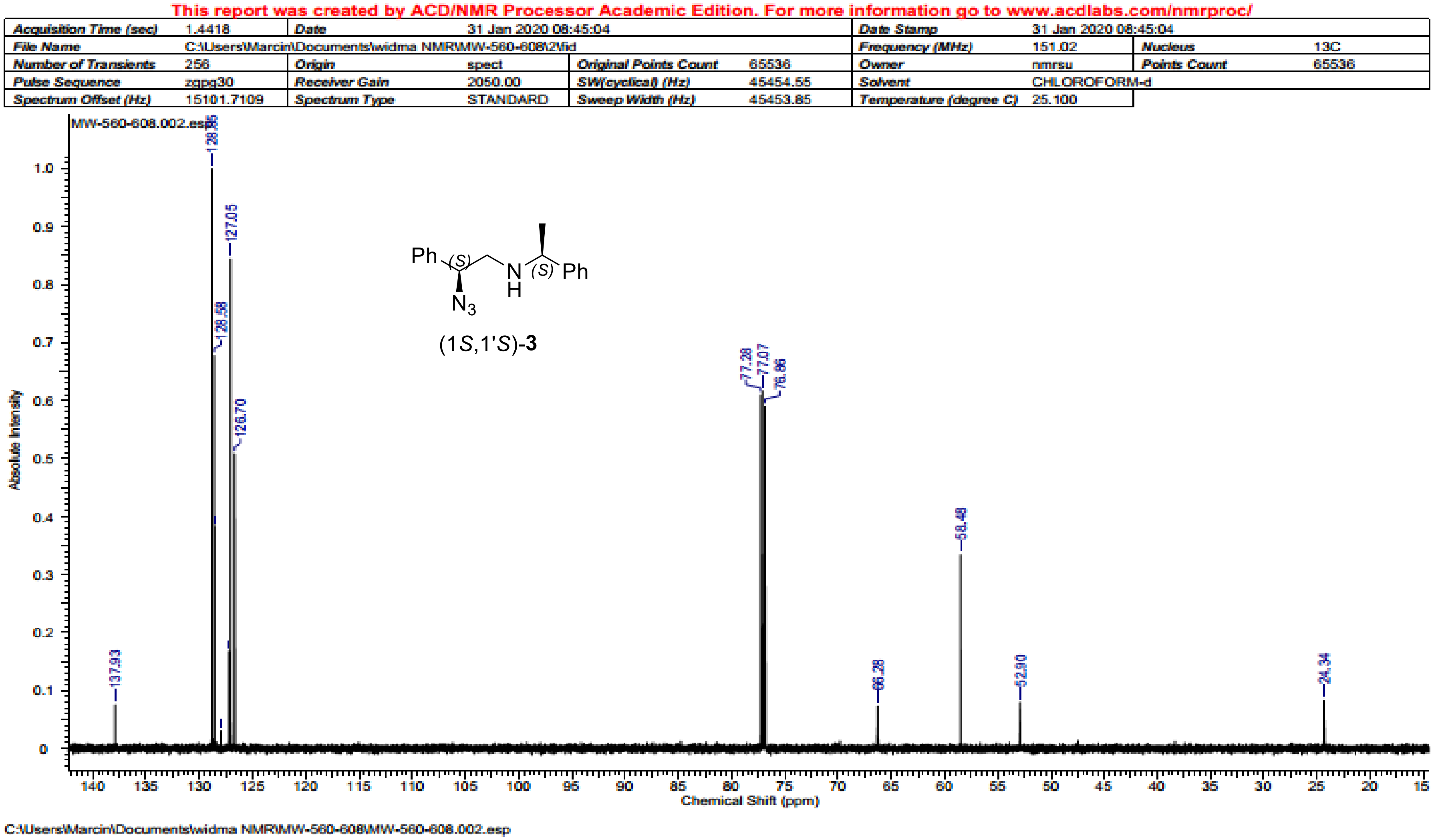

Figure S32. ^{13}C NMR spectrum (151 MHz, $CDCl_3$) for (1*S*,1'*S*)-**3**

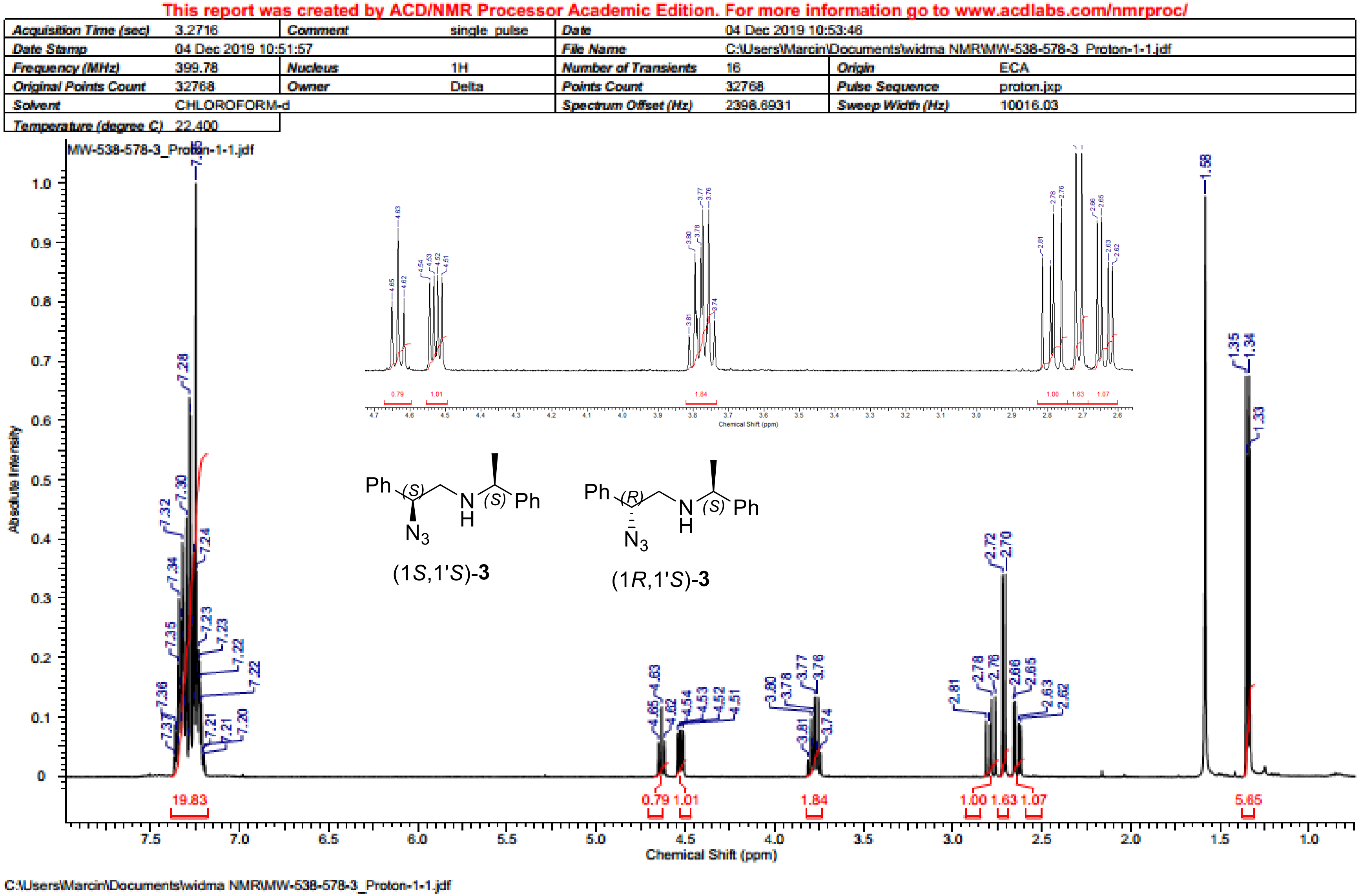

Figure S33. ^{1}H NMR spectrum (400 MHz, $CDCl_3$) for (1*R*,1’*S*)-**3** and (1*R*,1’*S*)-**3**

This report was created by ACD/NMR Processor Academic Edition. For more information go to www.acdlabs.com/nmrproc/

Acquisition Time (sec)	3.2716	*Comment*	single pulse	*Date*	11 Dec 2019 11:20:13		
Date Stamp	11 Dec 2019 11:18:23			*File Name*	C:\Users\Marcin\Documents\widma NMR\MW-546-587-1_Proton-2-1.jdf		
Frequency (MHz)	399.78	*Nucleus*	1H	*Number of Transients*	16	*Origin*	ECA
Original Points Count	32768	*Owner*	Delta	*Points Count*	32768	*Pulse Sequence*	proton.jxp
Solvent	CHLOROFORM-d			*Spectrum Offset (Hz)*	2398.6931	*Sweep Width (Hz)*	10016.03
Temperature (degree C)	22.700						

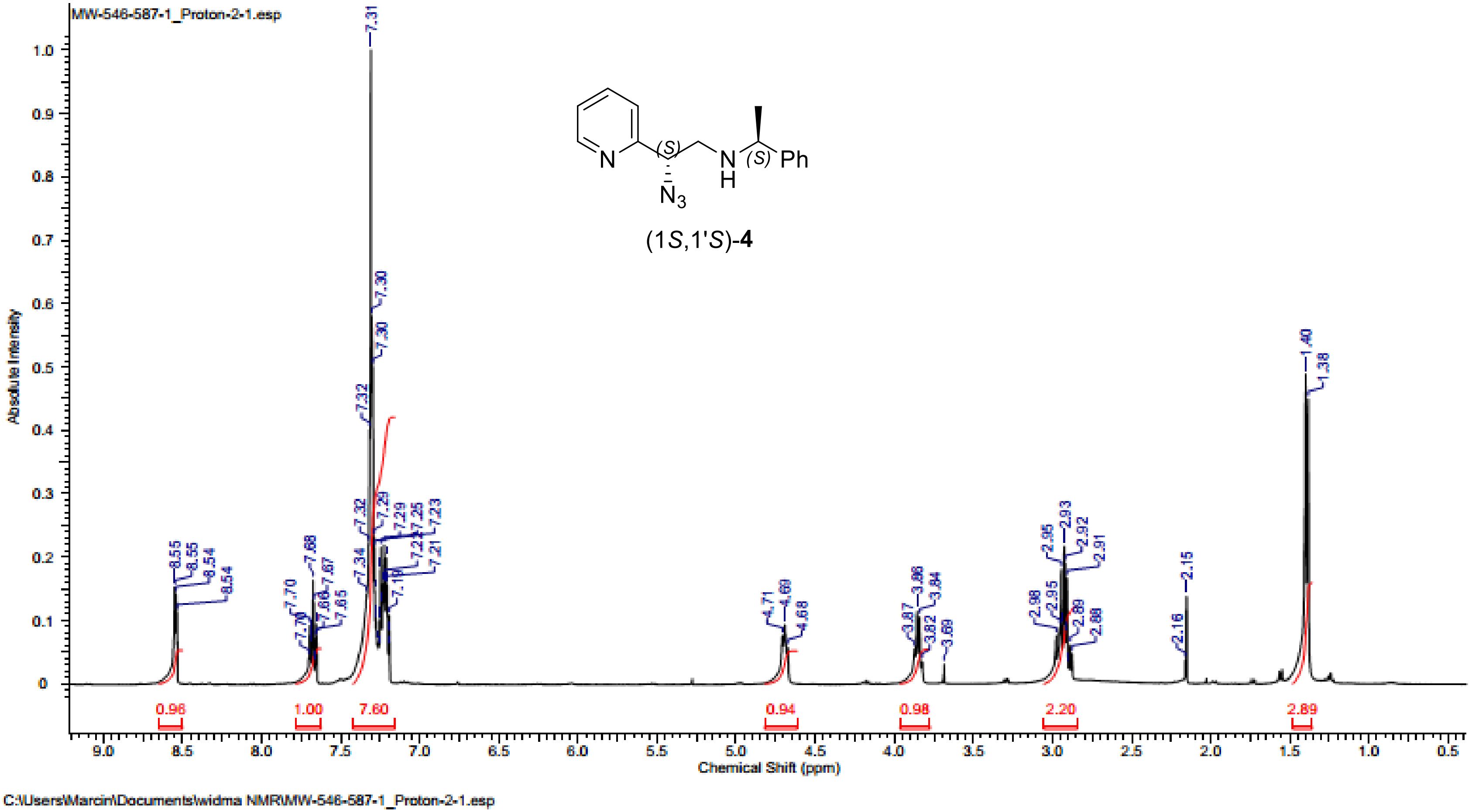

Figure S34. ^{1}H NMR spectrum (400 MHz, $CDCl_3$) for (1*S*,1’*S*)-**4**

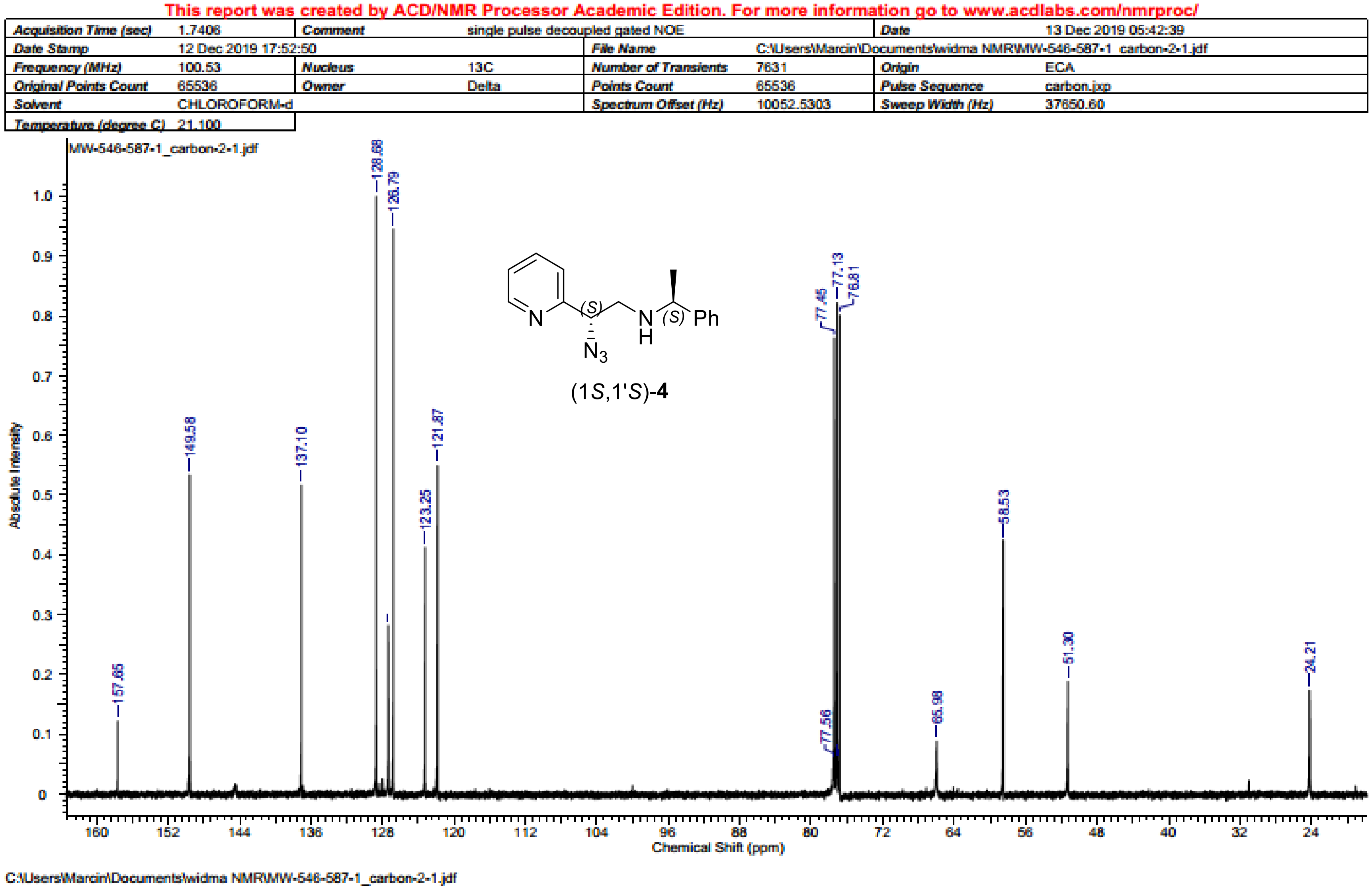

Figure S35. ^{13}C NMR spectrum (101 MHz, $CDCl_3$) for (1*S*,1'*S*)-**4**

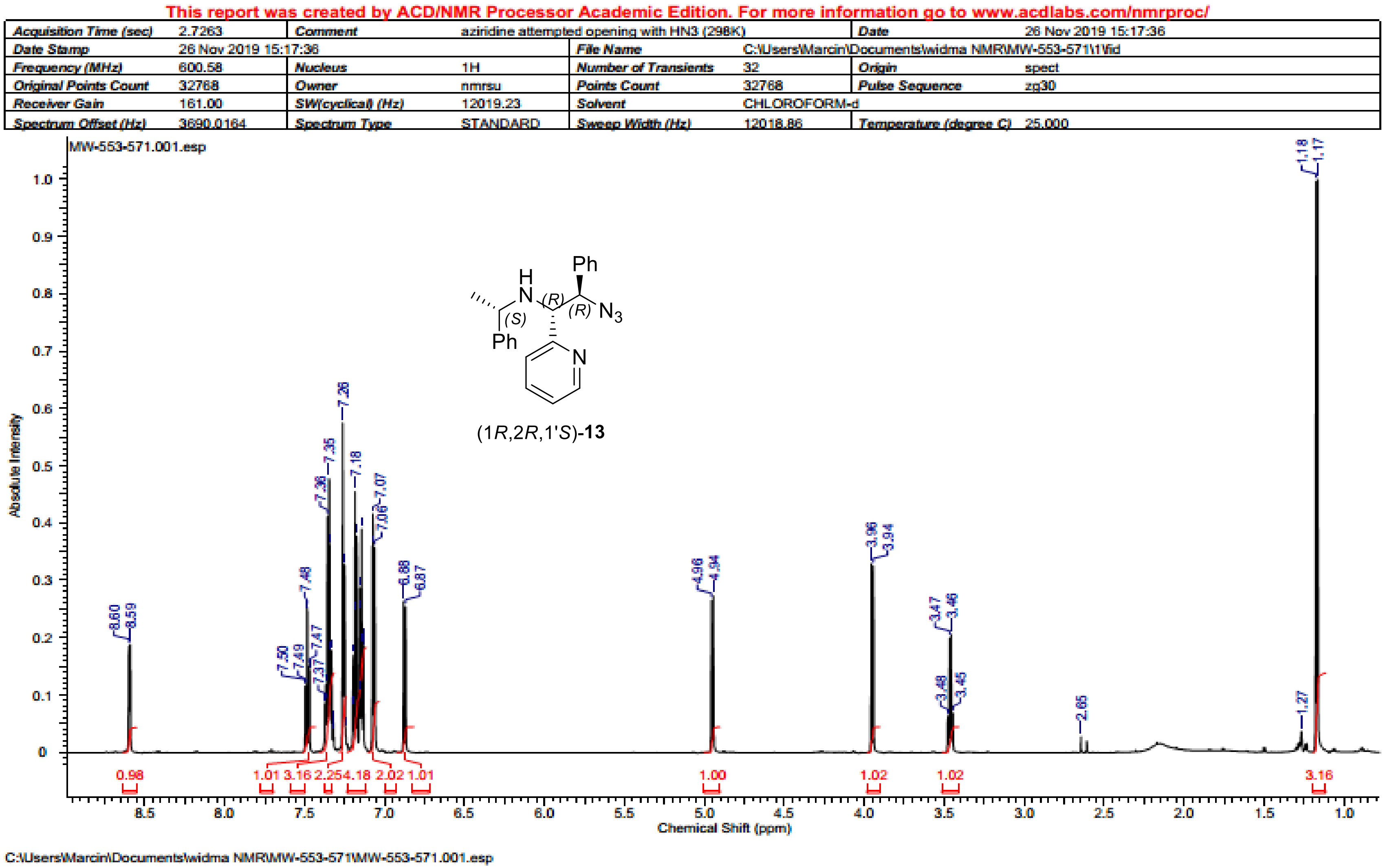

Figure S36. ^{1}H NMR spectrum (600 MHz, $CDCl_3$) for (1*R*,2*R*,1'*S*)-**13**

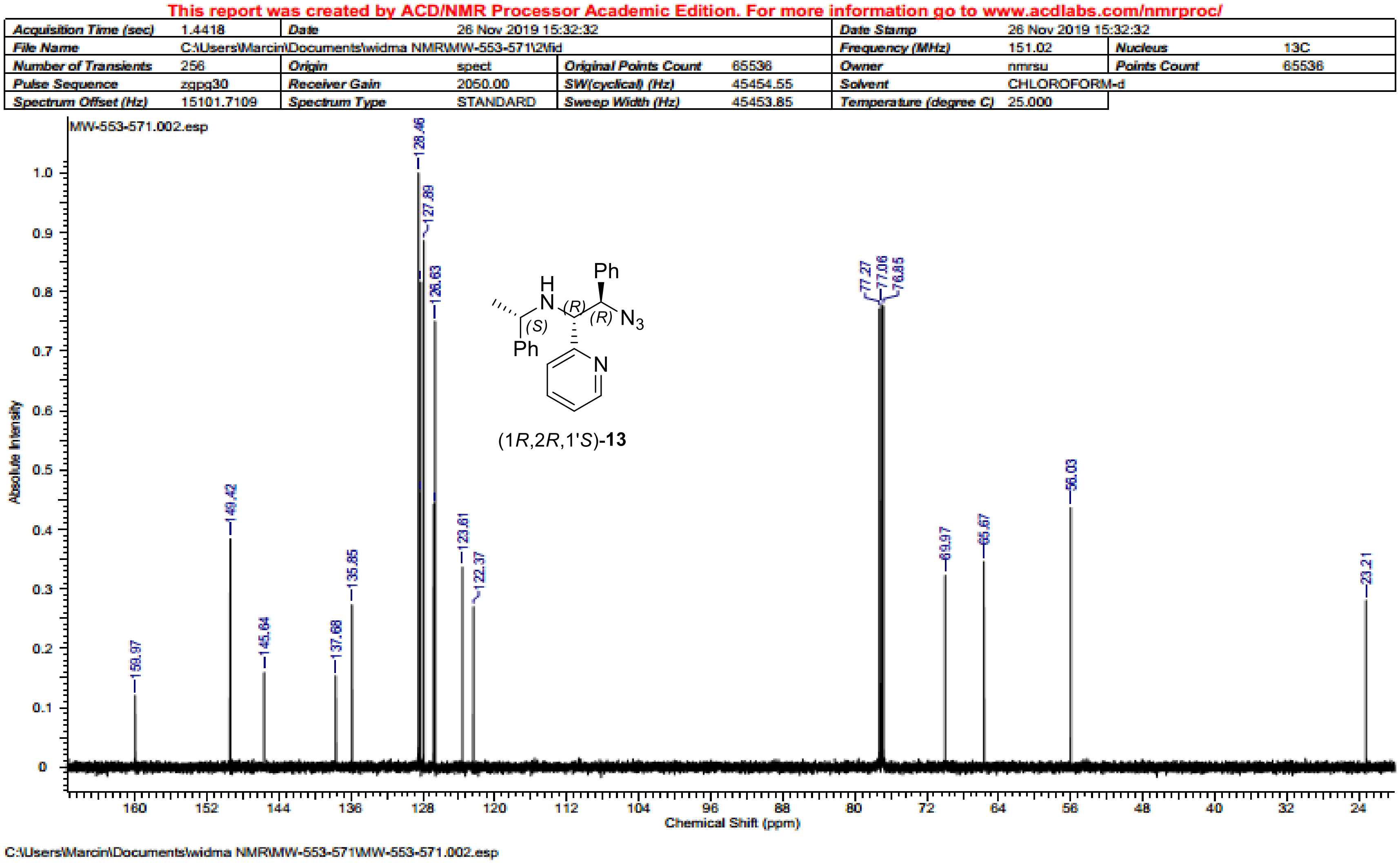

Figure S37. ^{13}C NMR spectrum (151 MHz, $CDCl_3$) for (1*R*,2*R*,1'*S*)-**13**

This report was created by ACD/NMR Processor Academic Edition. For more information go to www.acdlabs.com/nmrproc/

Acquisition Time (sec)	2.7263	*Comment*	MBA-NH-CH(Bipy)-CH(Ph)-N3 or regioisom.			*Date*	28 Nov 2019 17:49:04
Date Stamp	28 Nov 2019 17:49:04			*File Name*	C:\Users\Marcin\Documents\widma NMR\MW-585-572\1\fid		
Frequency (MHz)	600.58	*Nucleus*	1H	*Number of Transients*	32	*Origin*	spect
Original Points Count	32768	*Owner*	nmrsu	*Points Count*	32768	*Pulse Sequence*	zg30
Receiver Gain	203.00	*SW(cyclical) (Hz)*	12019.23	*Solvent*	CHLOROFORM-d		
Spectrum Offset (Hz)	3690.0164	*Spectrum Type*	STANDARD	*Sweep Width (Hz)*	12018.86	*Temperature (degree C)*	25.000

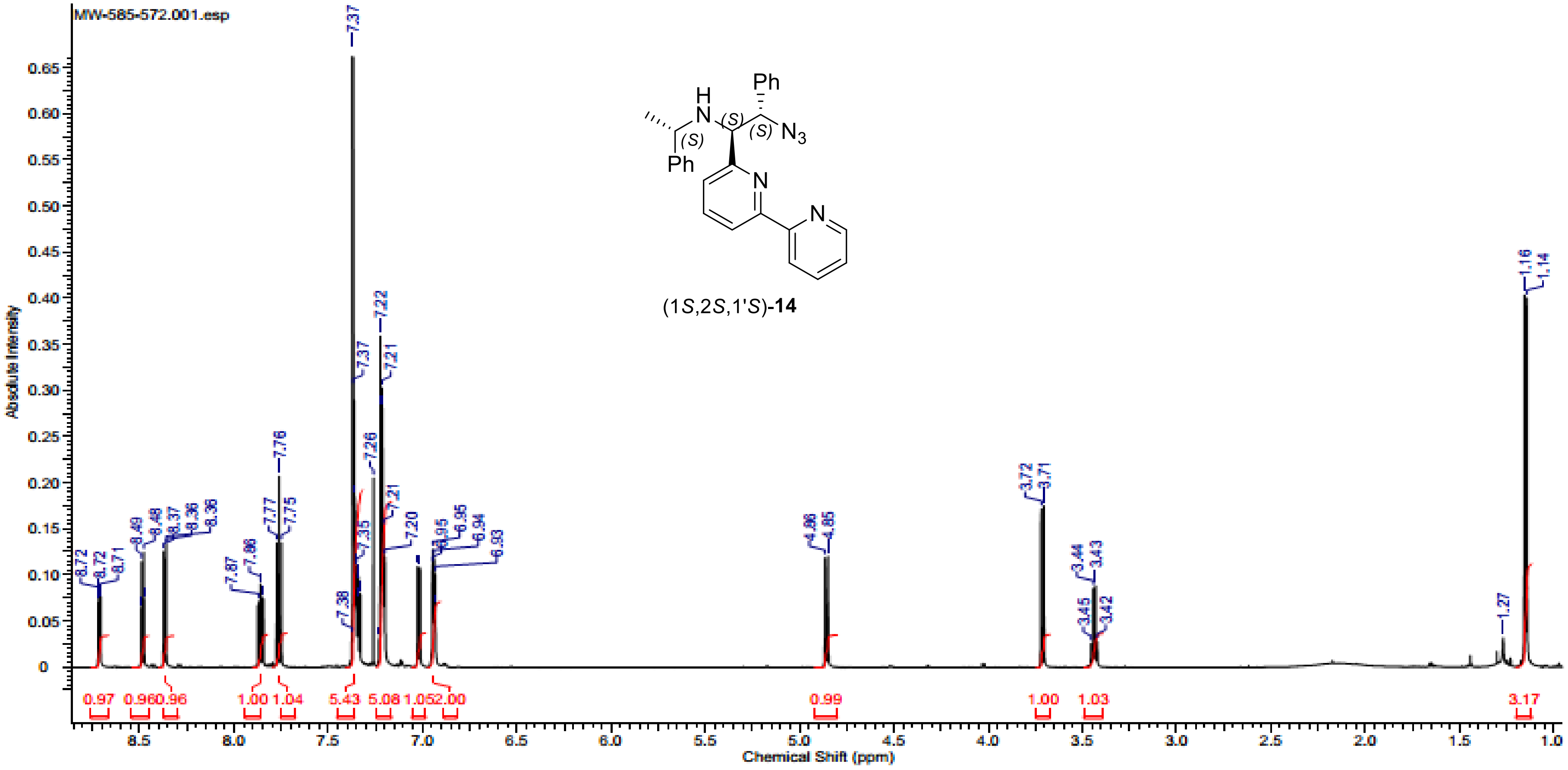

C:\Users\Marcin\Documents\widma NMR\MW-585-572\MW-585-572.001.esp

Figure S38. ^{1}H NMR spectrum (600 MHz, $CDCl_3$) for (1*S*,2*S*,1'*S*)-**14**

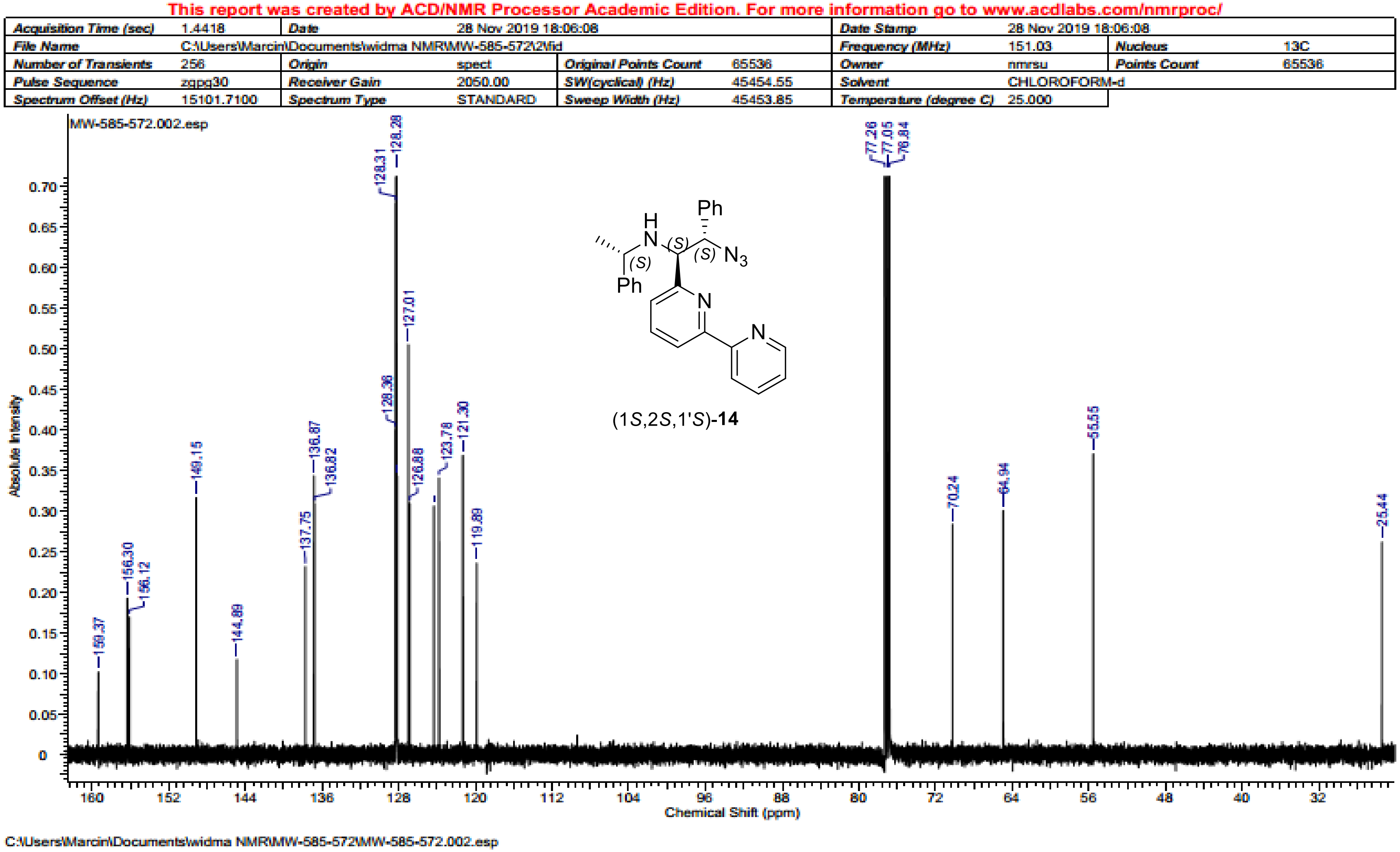

Figure S39. ^{13}C NMR spectrum (151 MHz, $CDCl_3$) for (1*S*,2*S*,1'*S*)-**14**

This report was created by ACD/NMR Processor Academic Edition. For more information go to www.acdlabs.com/nmrproc/

Acquisition Time (sec)	3.2716	*Comment*	single_pulse	*Date*	28 Aug 2019 08:00:53		
Date Stamp	28 Aug 2019 07:59:04			*File Name*	C:\Users\Marcin\Documents\widma NMR\MW-479-498-2_Proton-1-1.jdf		
Frequency (MHz)	399.78	*Nucleus*	1H	*Number of Transients*	16	*Origin*	ECA
Original Points Count	32768	*Owner*	Delta	*Points Count*	32768	*Pulse Sequence*	proton.jxp
Solvent	CHLOROFORM-d			*Spectrum Offset (Hz)*	2398.6931	*Sweep Width (Hz)*	10016.03
Temperature (degree C)	23.000						

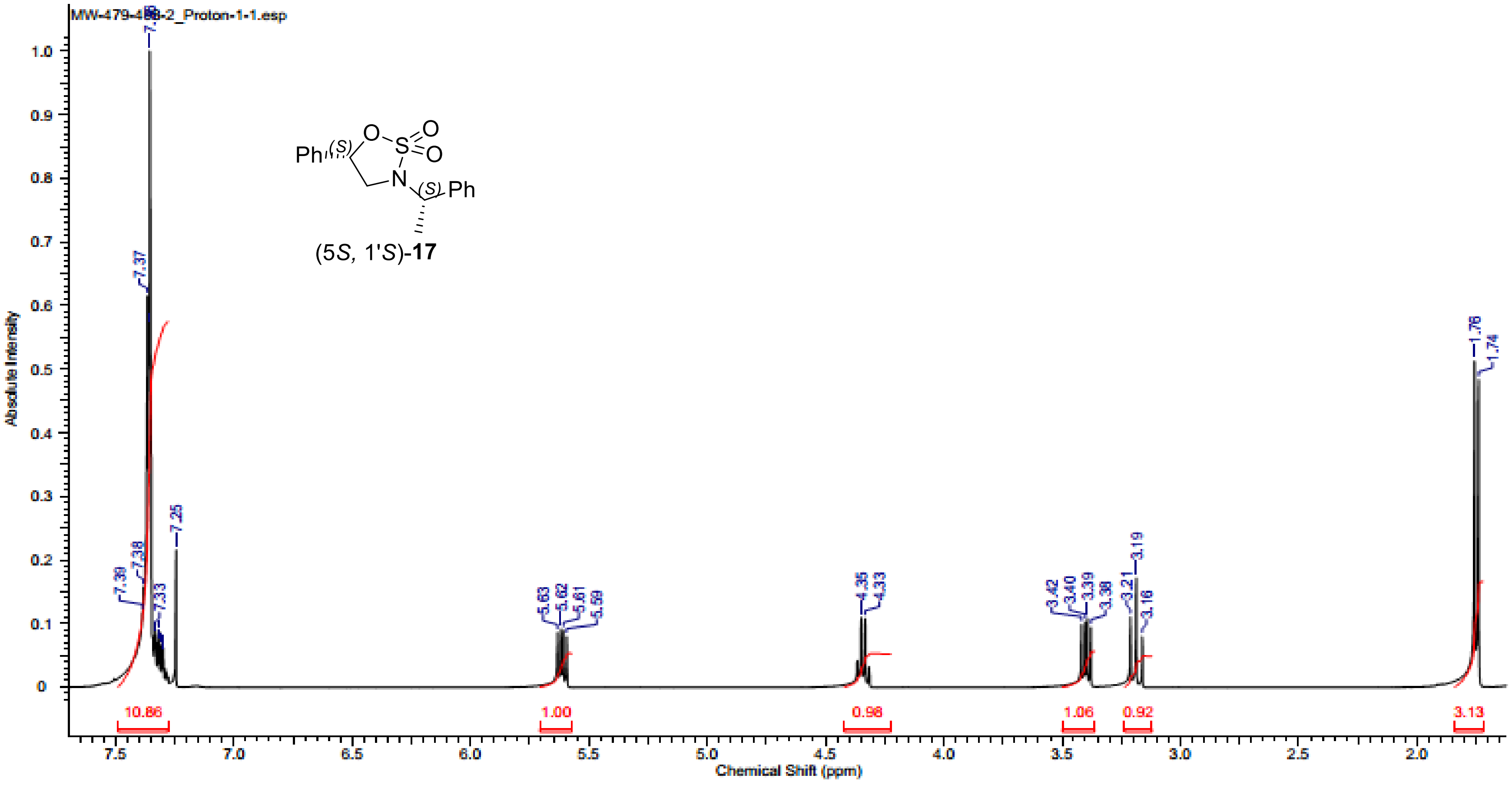

C:\Users\Marcin\Documents\widma NMR\MW-479-498-2_Proton-1-1.esp

Figure S40. ^{1}H NMR spectrum (400 MHz, $CDCl_3$) for (5*S*,1'*S*)-**17**

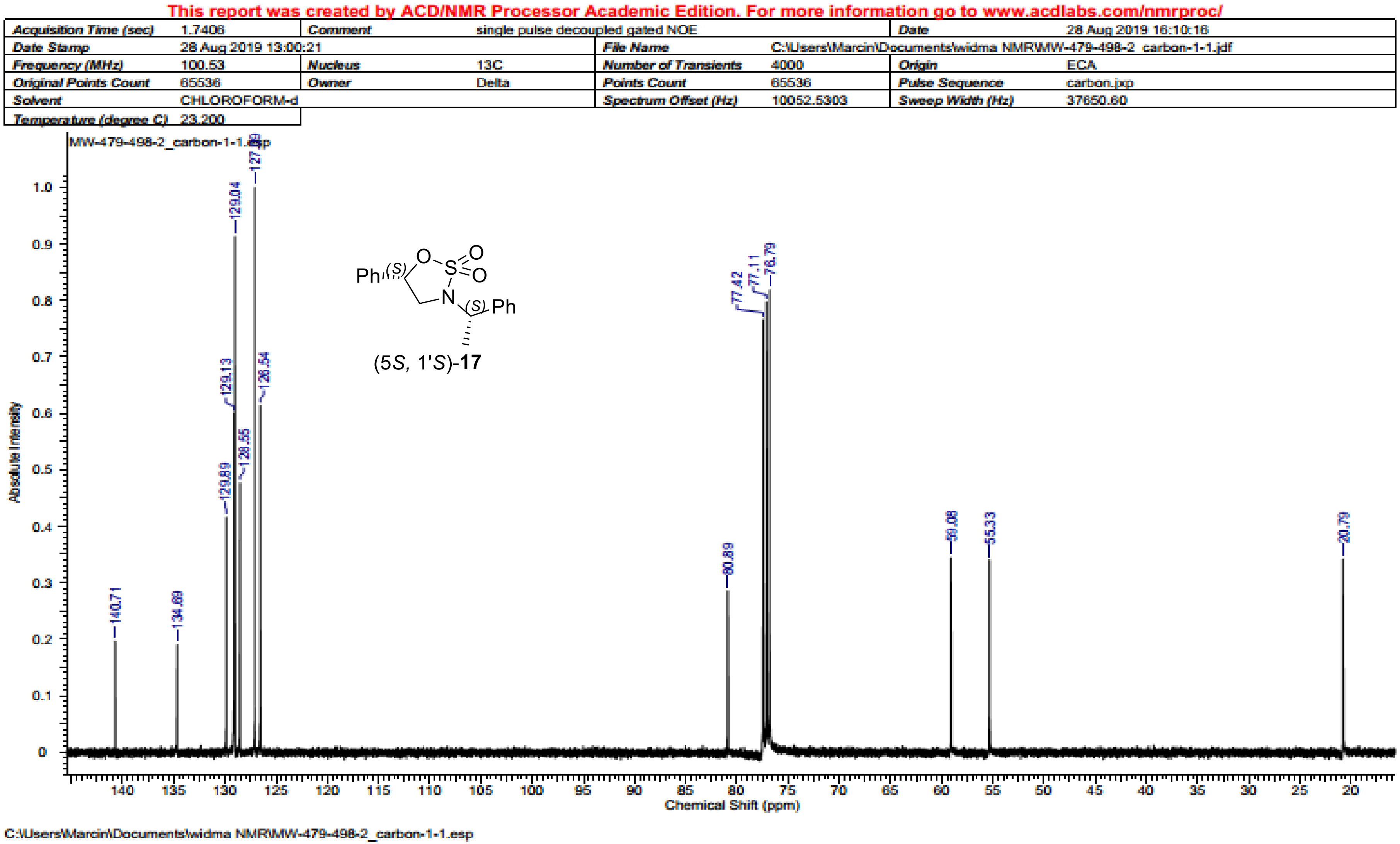

Figure S41. ^{13}C NMR spectrum (101 MHz, $CDCl_3$) for (5*S*,1’*S*)-**17**

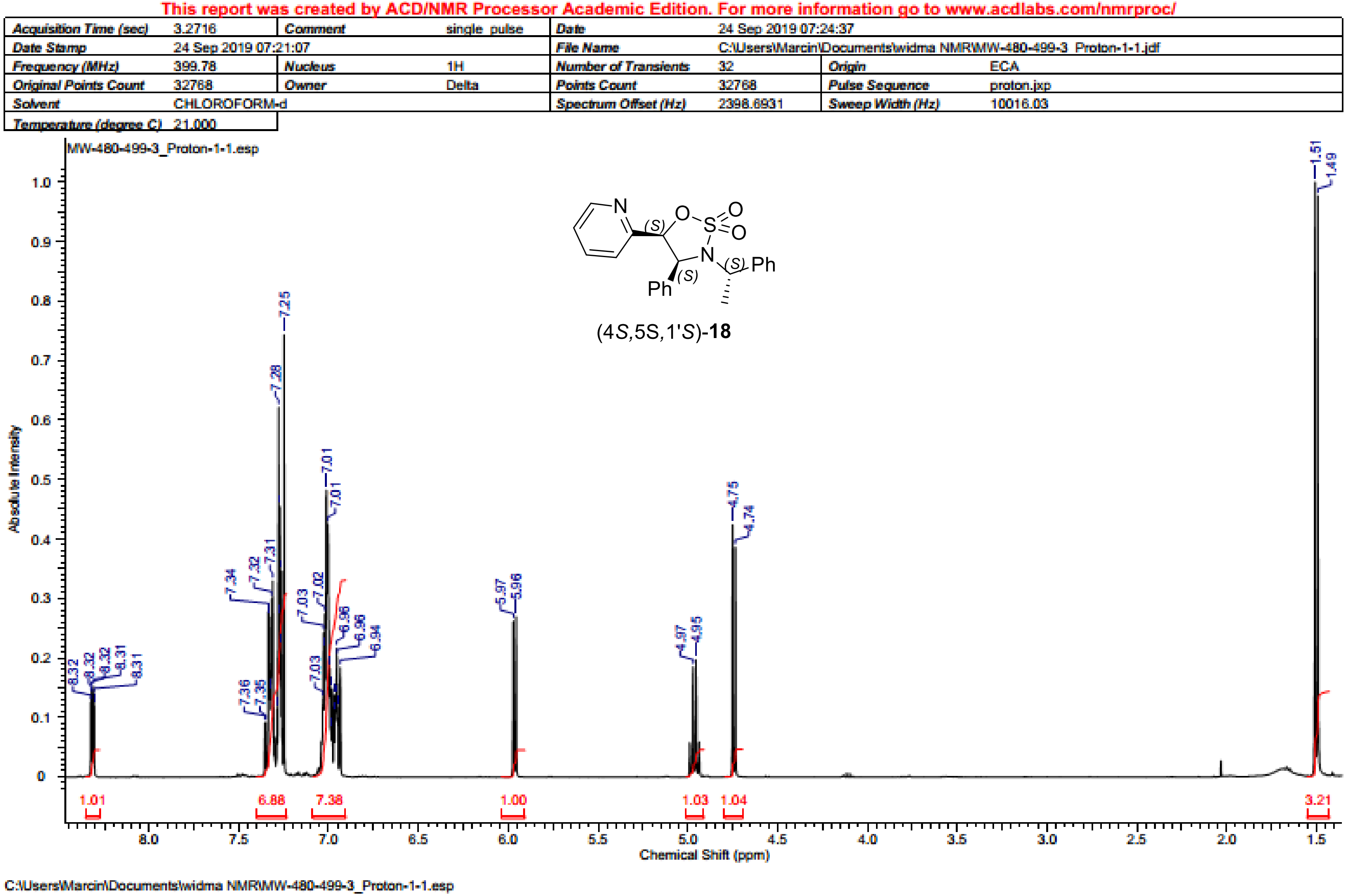

Figure S42. ^{1}H NMR spectrum (400 MHz, $CDCl_3$) for (4*S*,5*S*,1'*S*)-**18**

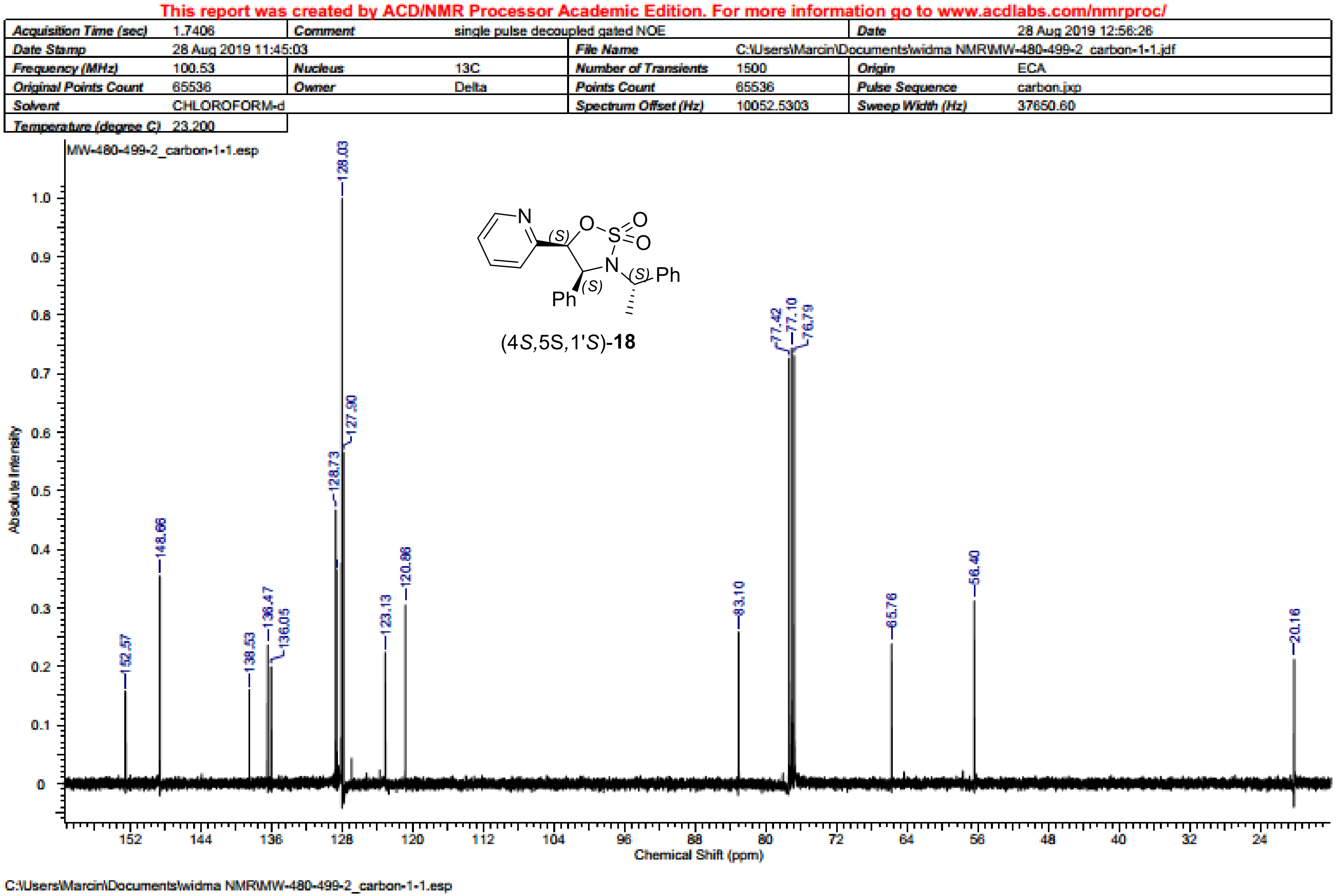

Figure S43. ^{13}C NMR spectrum (101 MHz, $CDCl_3$) for (4*S*,5*S*,1'*S*)-**18**

This report was created by ACD/NMR Processor Academic Edition. For more information go to www.acdlabs.com/nmrproc/

Acquisition Time (sec)	3.2716	Comment	single_pulse	Date	03 Oct 2019 08:44:45		
Date Stamp	03 Oct 2019 08:41:15			File Name	C:\Users\Marcin\Documents\widma NMR\MW-491-516-1_Proton-1-1.jdf		
Frequency (MHz)	399.78	Nucleus	1H	Number of Transients	32	Origin	ECA
Original Points Count	32768	Owner	Delta	Points Count	32768	Pulse Sequence	proton.jxp
Solvent	CHLOROFORM-d			Spectrum Offset (Hz)	2398.6931	Sweep Width (Hz)	10016.03
Temperature (degree C)	22.700						

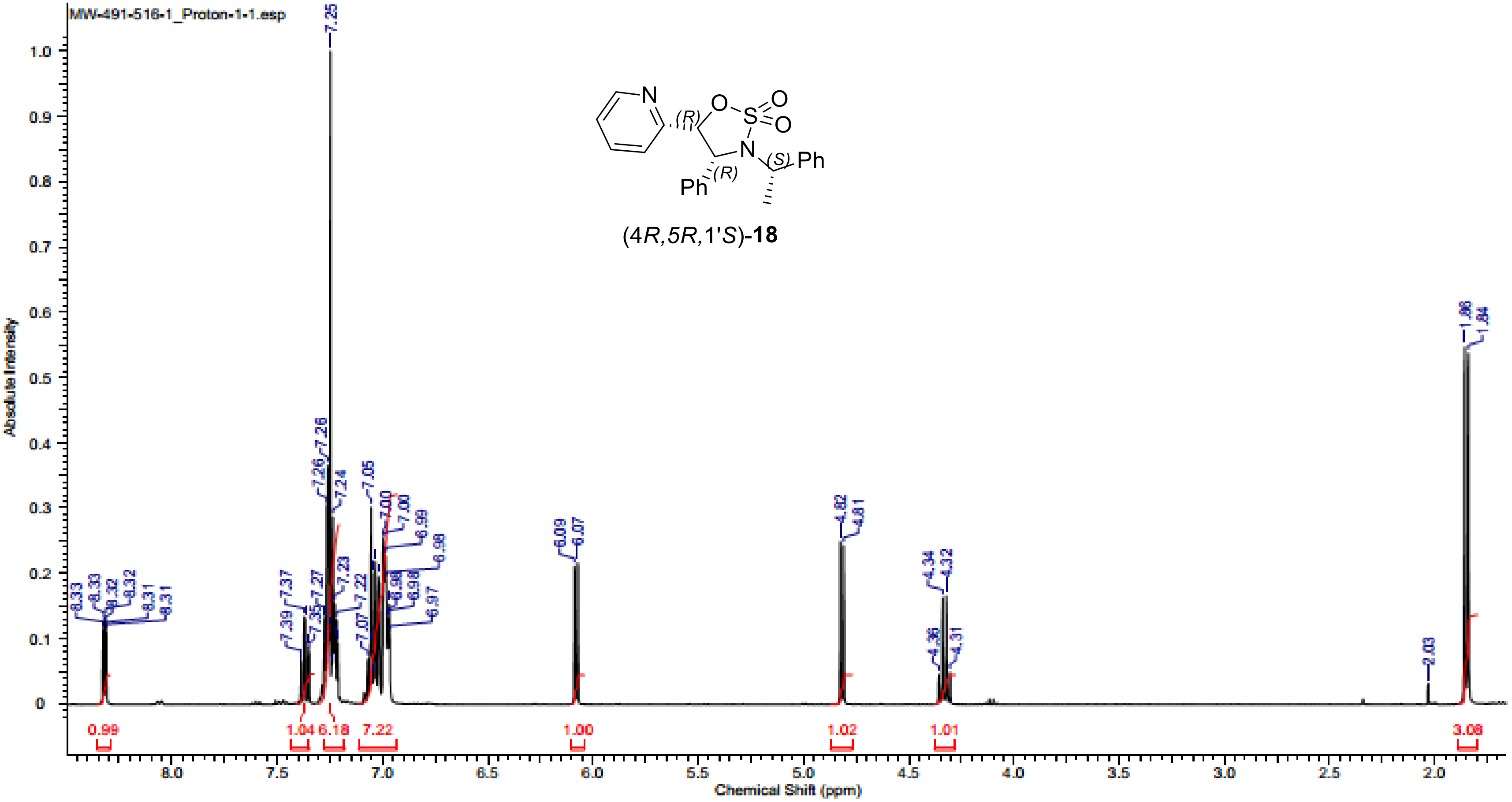

C:\Users\Marcin\Documents\widma NMR\MW-491-516-1_Proton-1-1.esp

Figure S44. ^{1}H NMR spectrum (400 MHz, $CDCl_3$) for (4*R*,5*R*,1'*S*)-**18**

This report was created by ACD/NMR Processor Academic Edition. For more information go to www.acdlabs.com/nmrproc/

Acquisition Time (sec)	1.7406	*Comment*	single pulse decoupled gated NOE			*Date*	07 Oct 2019 15:13:29
Date Stamp	07 Oct 2019 13:38:24			*File Name*	C:\Users\Marcin\Documents\widma NMR\MW-491-516-1_carbon-1-1.jdf		
Frequency (MHz)	100.53	*Nucleus*	13C	*Number of Transients*	2000	*Origin*	ECA
Original Points Count	65536	*Owner*	Delta	*Points Count*	65536	*Pulse Sequence*	carbon.jxp
Solvent	CHLOROFORM-d			*Spectrum Offset (Hz)*	10052.5303	*Sweep Width (Hz)*	37650.60
Temperature (degree C)	22.900						

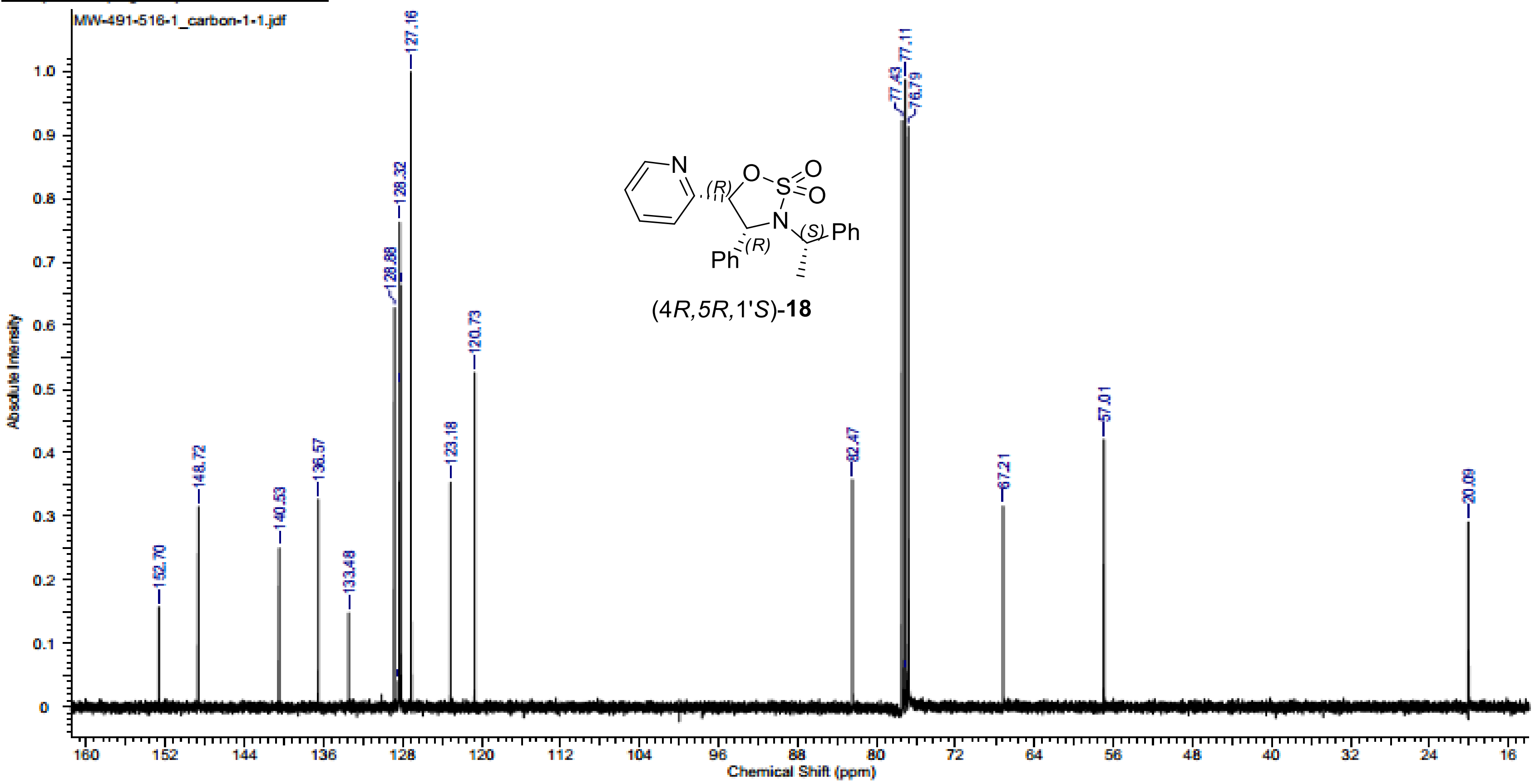

C:\Users\Marcin\Documents\widma NMR\MW-491-516-1_carbon-1-1.jdf

Figure S45. ^{13}C NMR spectrum (101 MHz, $CDCl_3$) for (4*R*,5*R*,1'*S*)-**18**

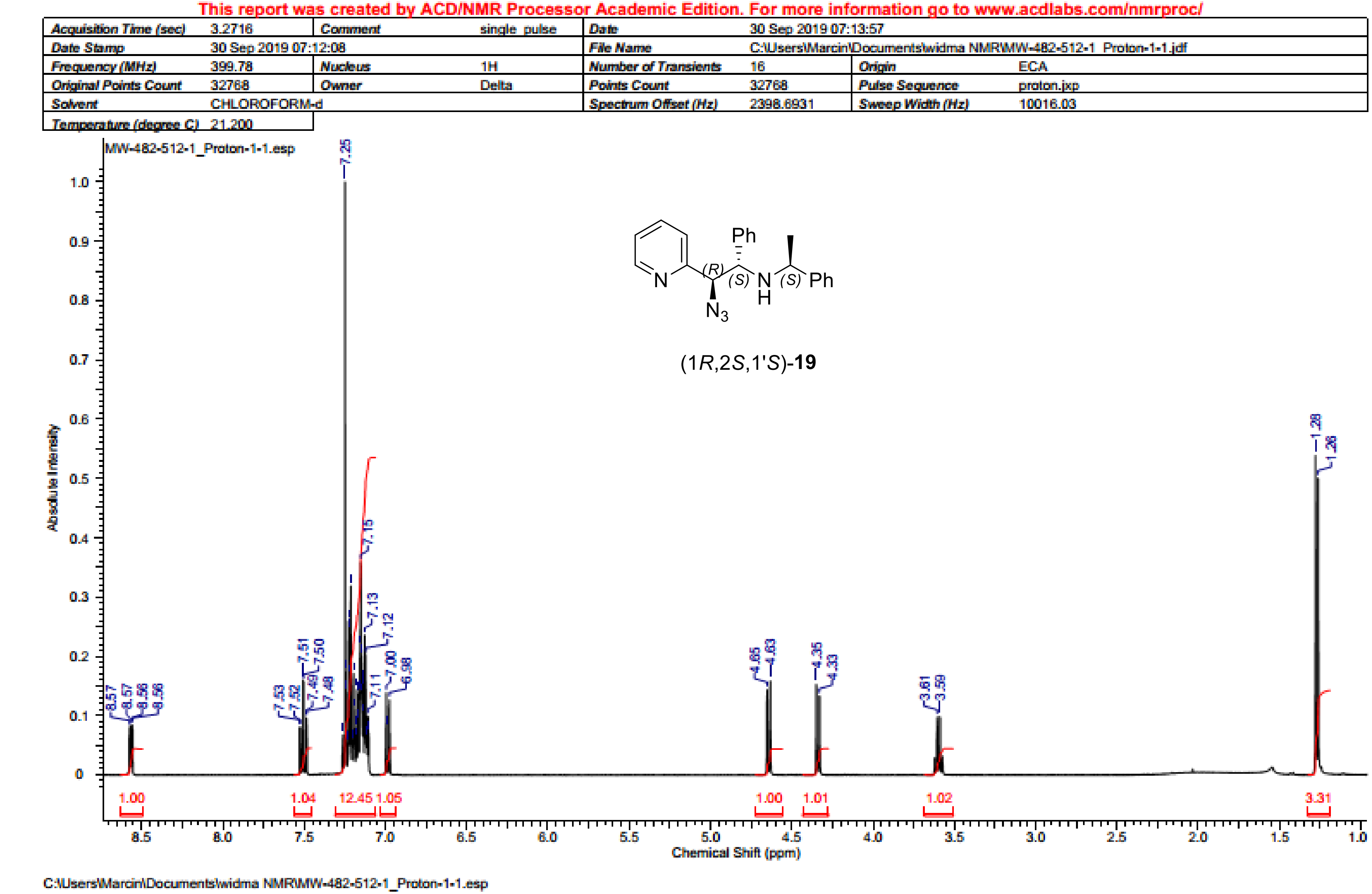

Figure S46. ^{1}H NMR spectrum (400 MHz, $CDCl_3$) for (1*R*,2*S*,1’*S*)-**19**

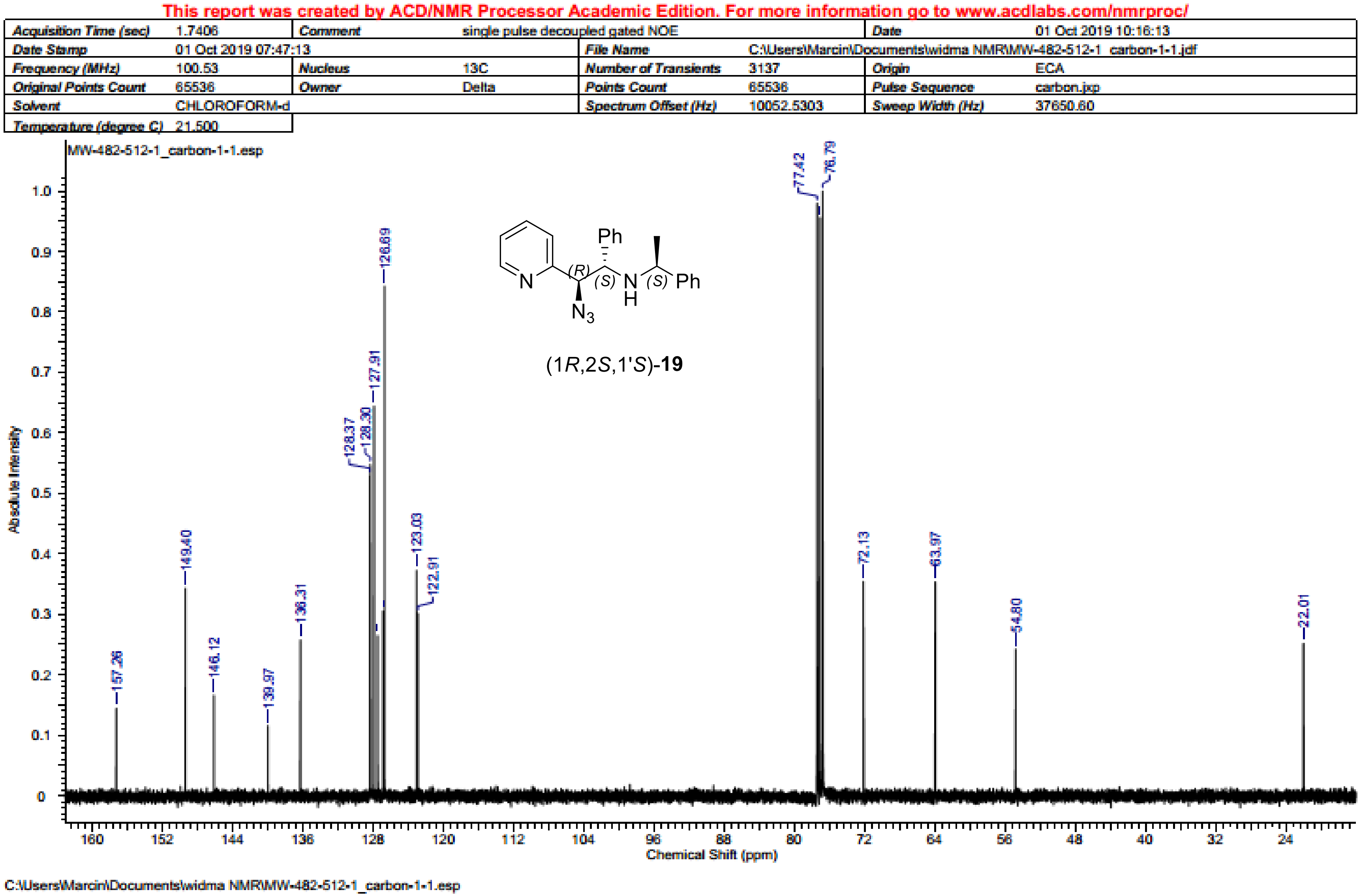

Figure S47. ^{13}C NMR spectrum (101 MHz, $CDCl_3$) for (1*R*,2*S*,1'*S*)-**19**

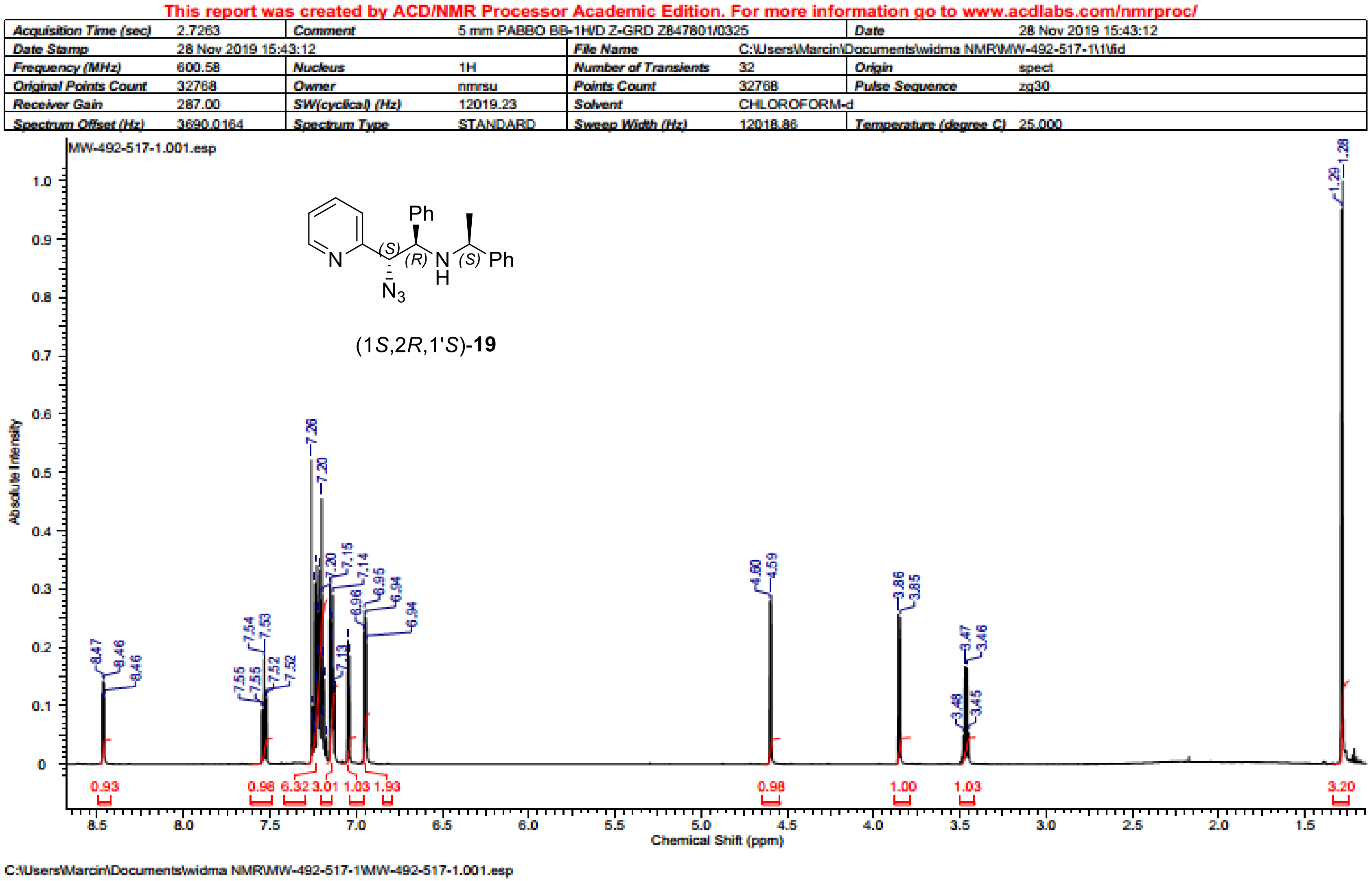

Figure S48. ^{1}H NMR spectrum (600 MHz, $CDCl_3$) for (1*S*,2*R*,1'*S*)-**19**

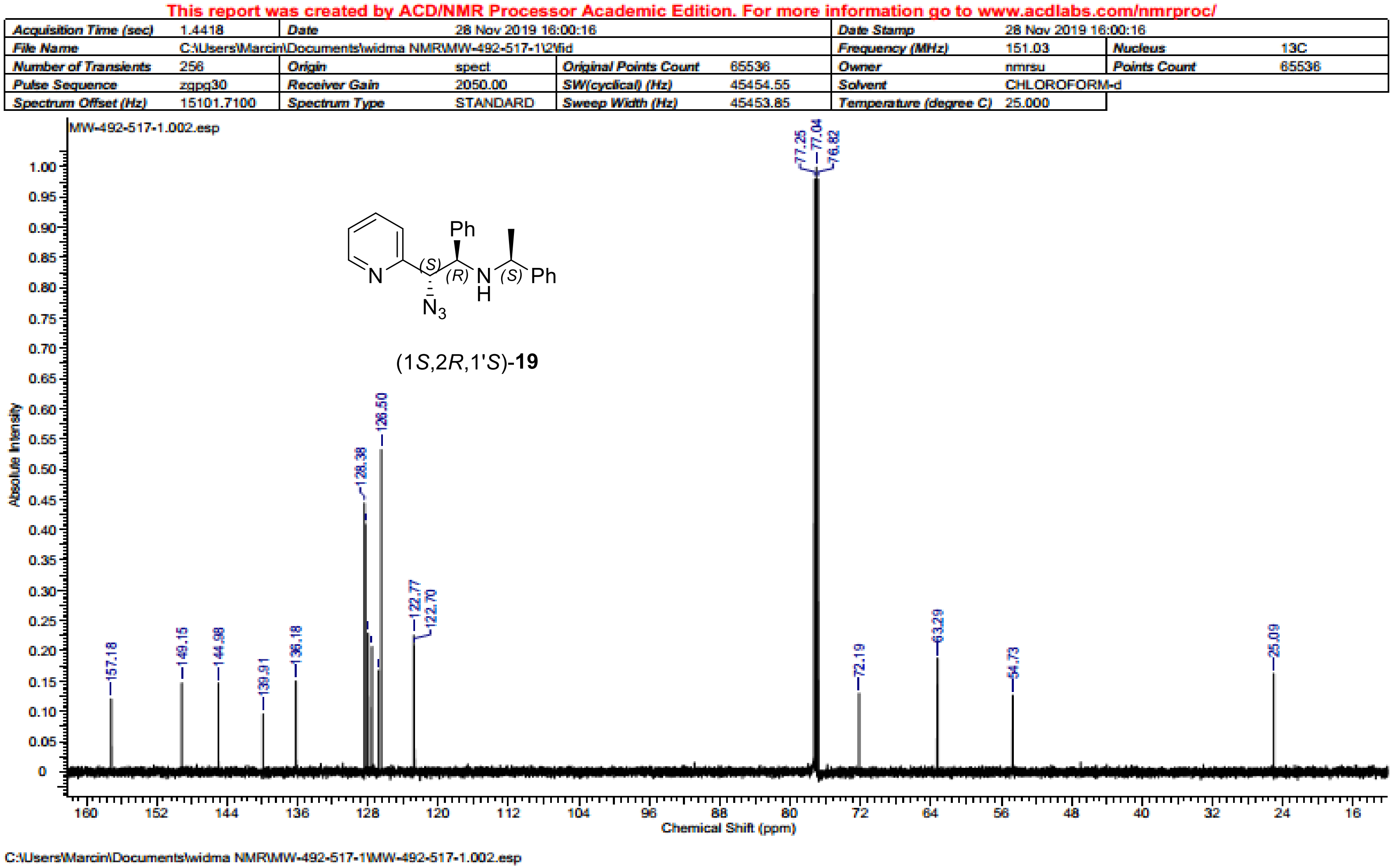

Figure S49. ^{13}C NMR spectrum (151 MHz, $CDCl_3$) for (1*S*,2*R*,1’*S*)-**19**

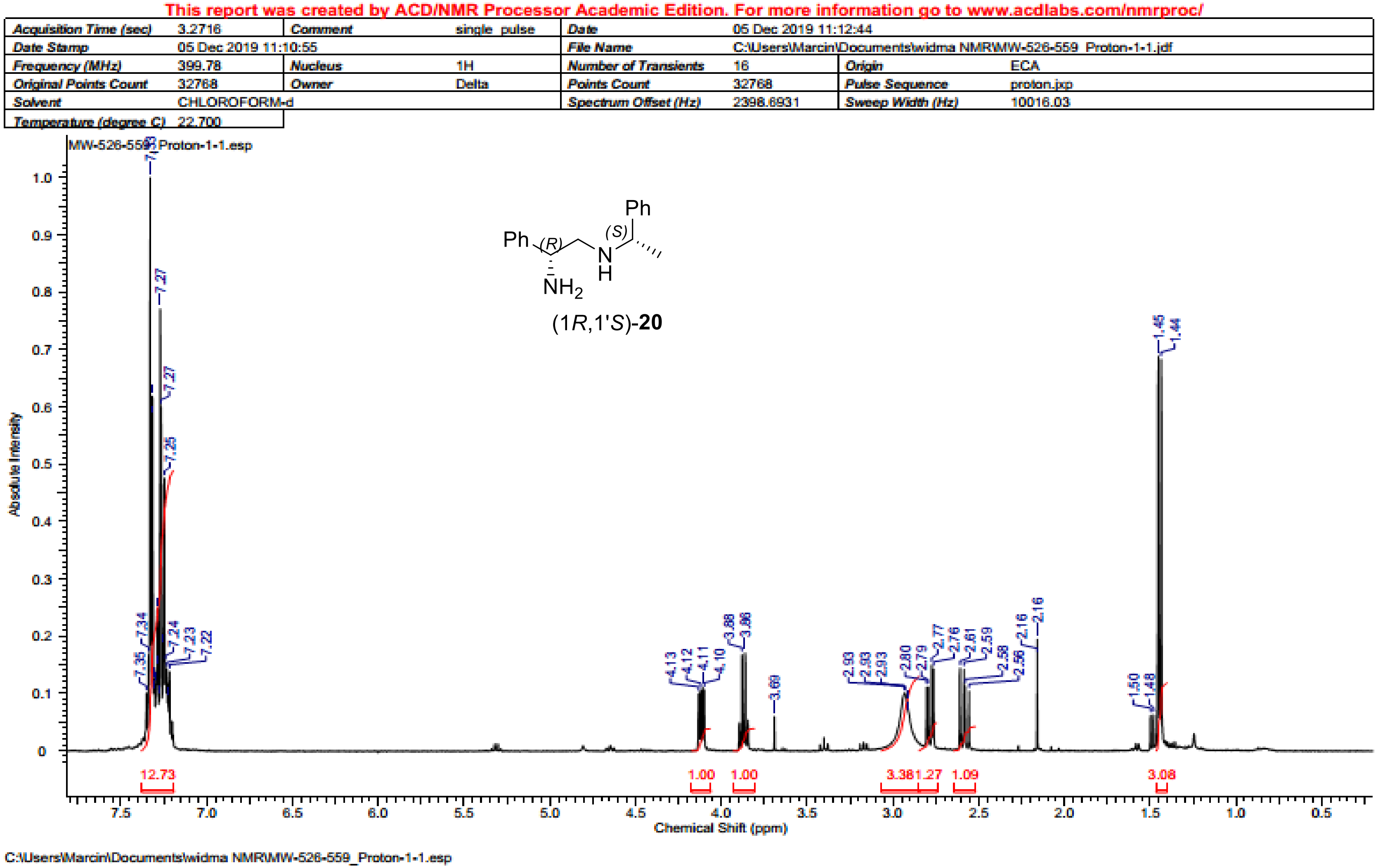

Figure S50. ^{1}H NMR spectrum (400 MHz, $CDCl_3$) for (1*R*,1'*S*)-**20**

This report was created by ACD/NMR Processor Academic Edition. For more information go to www.acdlabs.com/nmrproc/

Acquisition Time (sec)	1.7406	*Comment*	single pulse decoupled gated NOE			*Date*	14 Dec 2019 03:58:24
Date Stamp	13 Dec 2019 16:30:31			*File Name*	C:\Users\Marcin\Documents\widma NMR\MW-526-559_carbon-1-1.jdf		
Frequency (MHz)	100.53	*Nucleus*	13C	*Number of Transients*	7168	*Origin*	ECA
Original Points Count	65536	*Owner*	Delta	*Points Count*	65536	*Pulse Sequence*	carbon.jxp
Solvent	CHLOROFORM-d			*Spectrum Offset (Hz)*	10052.5303	*Sweep Width (Hz)*	37650.60
Temperature (degree C)	21.800						

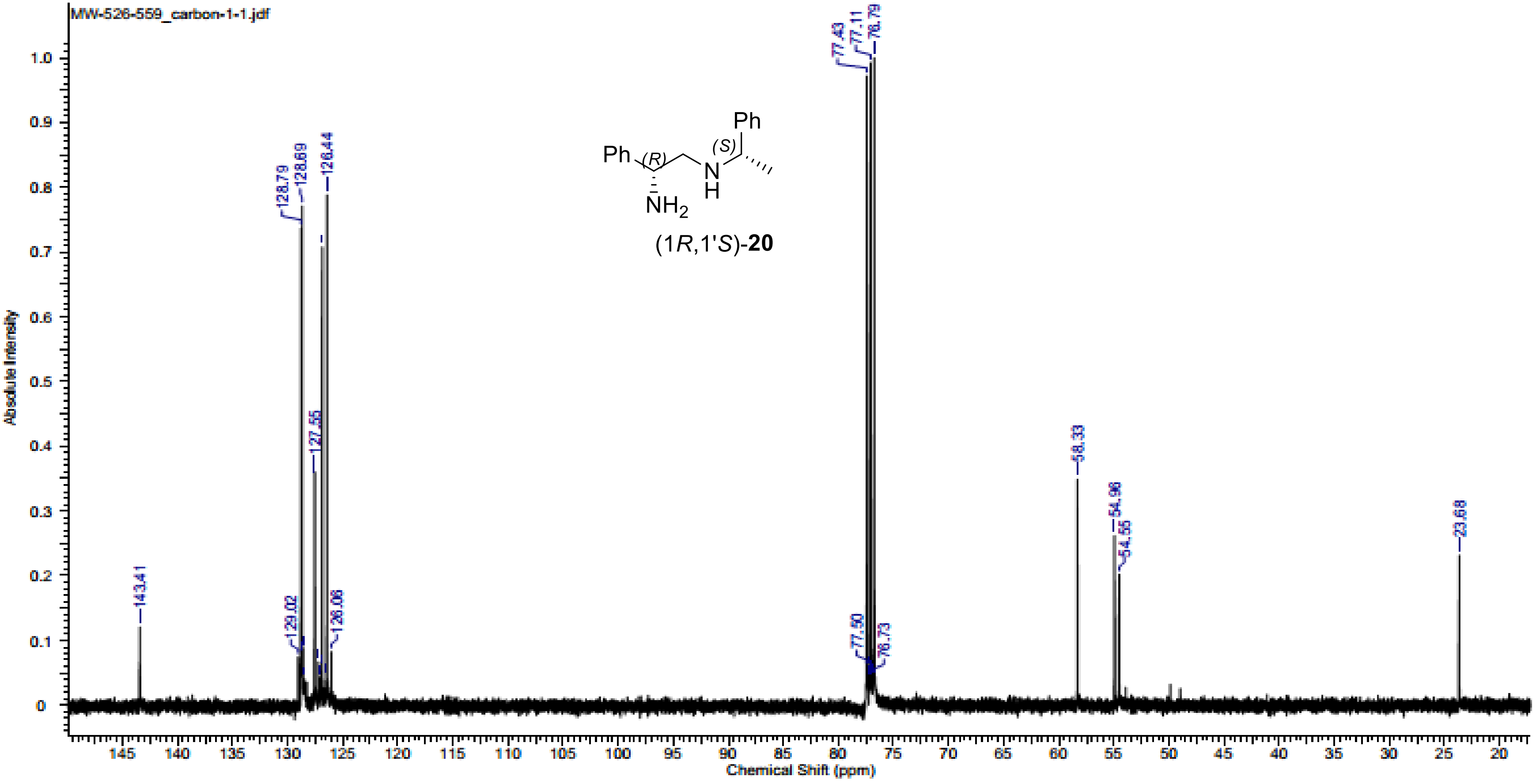

C:\Users\Marcin\Documents\widma NMR\MW-526-559_carbon-1-1.jdf

Figure S51. ^{13}C NMR spectrum (101 MHz, $CDCl_3$) for (1*R*,1'*S*)-**20**

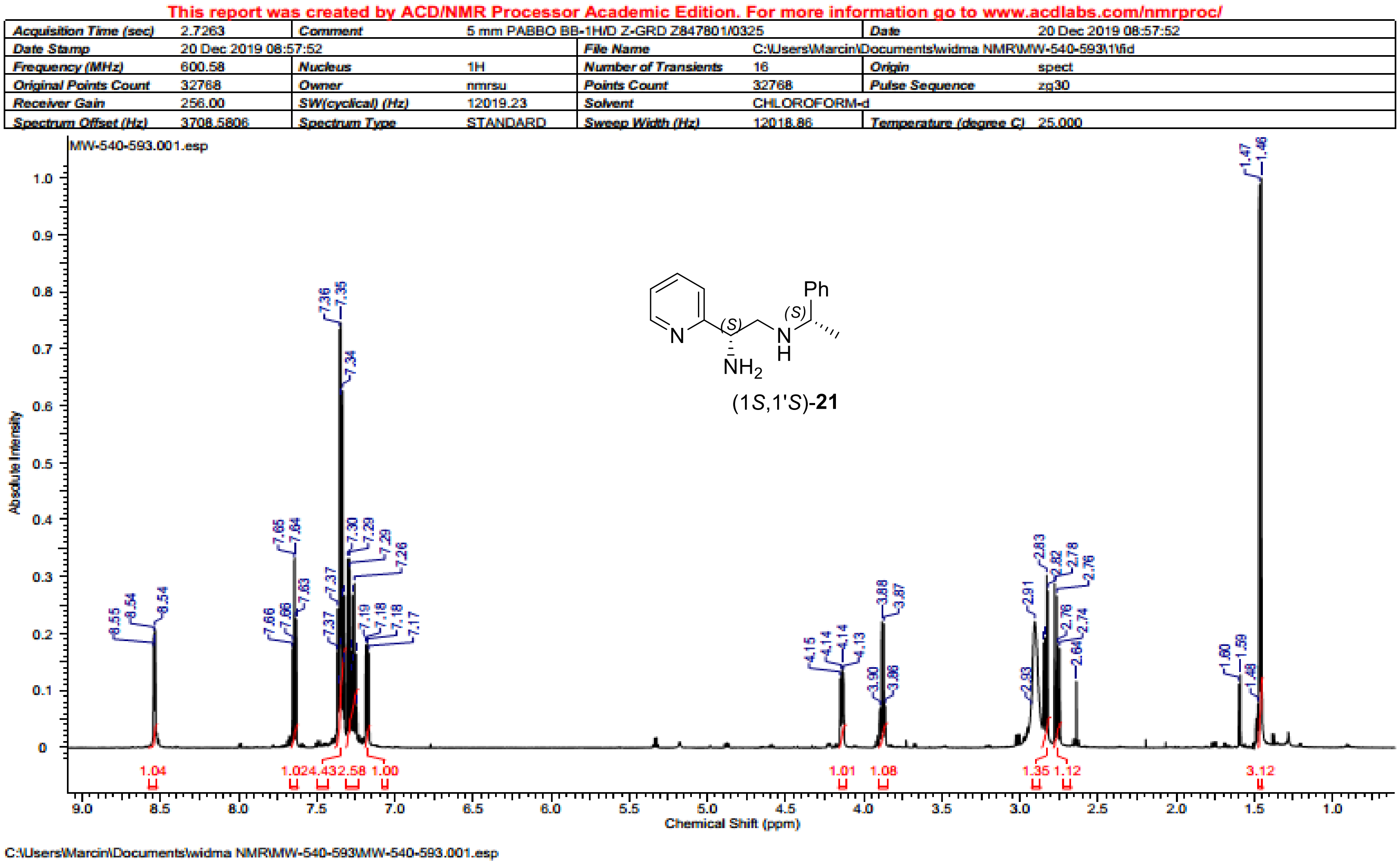

Figure S52. ^{1}H NMR spectrum (600 MHz, $CDCl_3$) for (1*S*,1'*S*)-**21**

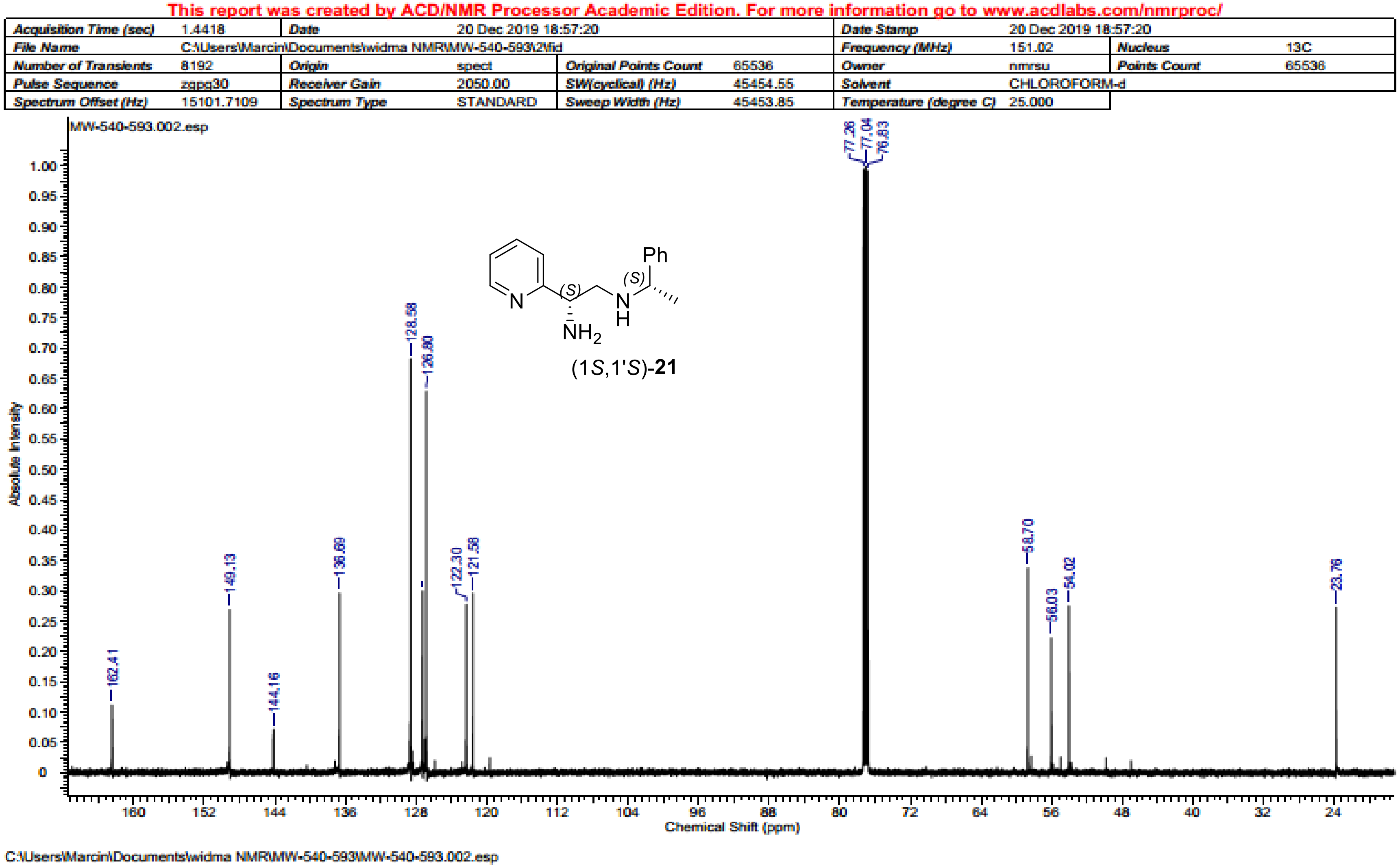

Figure S53. ^{13}C NMR spectrum (151 MHz, $CDCl_3$) for (1*S*,1'*S*)-**21**

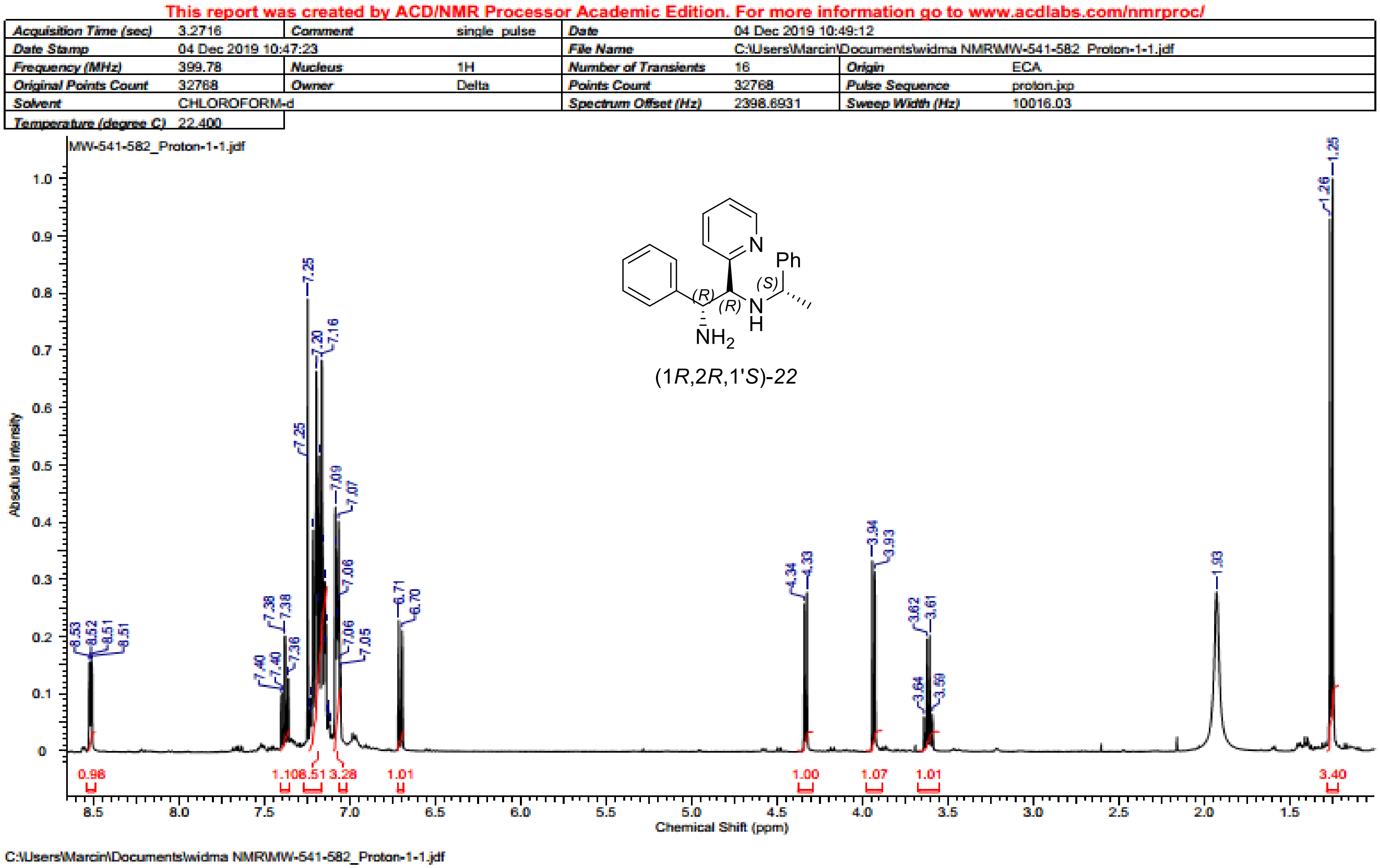

Figure S54. ^{1}H NMR spectrum (400 MHz, $CDCl_3$) for (1*R*,2*R*,1'*S*)-**22**

This report was created by ACD/NMR Processor Academic Edition. For more information go to www.acdlabs.com/nmrproc/

Acquisition Time (sec)	1.7406	Comment	single pulse decoupled gated NOE			Date	05 Dec 2019 17:31:02
Date Stamp	05 Dec 2019 15:55:57			File Name	C:\Users\Marcin\Documents\widma NMR\MW-541-582_carbon-1-1.jdf		
Frequency (MHz)	100.53	Nucleus	13C	Number of Transients	2000	Origin	ECA
Original Points Count	65536	Owner	Delta	Points Count	65536	Pulse Sequence	carbon.jxp
Solvent	CHLOROFORM-d			Spectrum Offset (Hz)	10052.5303	Sweep Width (Hz)	37650.60
Temperature (degree C)	22.300						

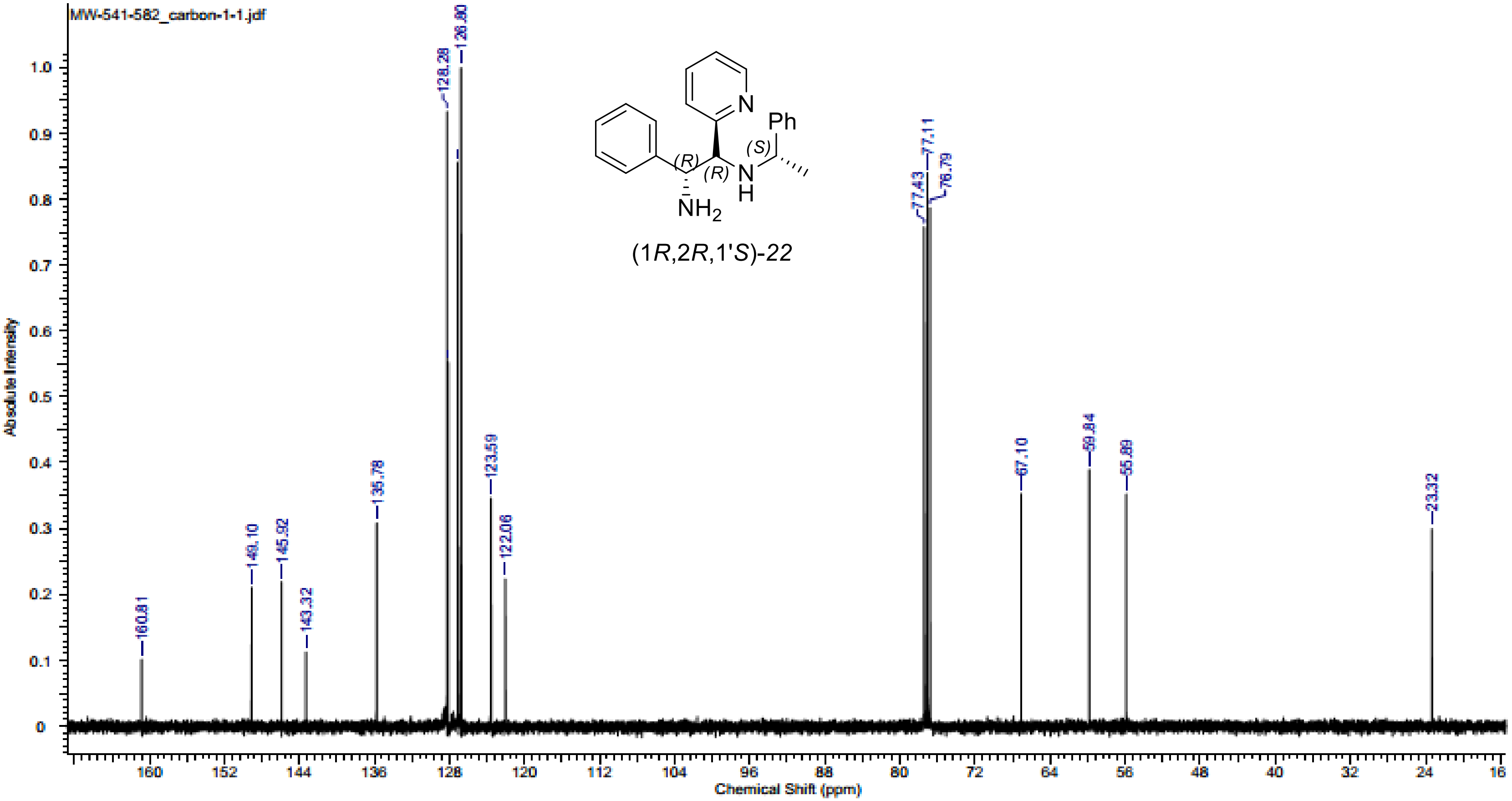

C:\Users\Marcin\Documents\widma NMR\MW-541-582_carbon-1-1.jdf

Figure S55. ^{13}C NMR spectrum (101 MHz, $CDCl_3$) for (1*R*,2*R*,1'*S*)-**22**

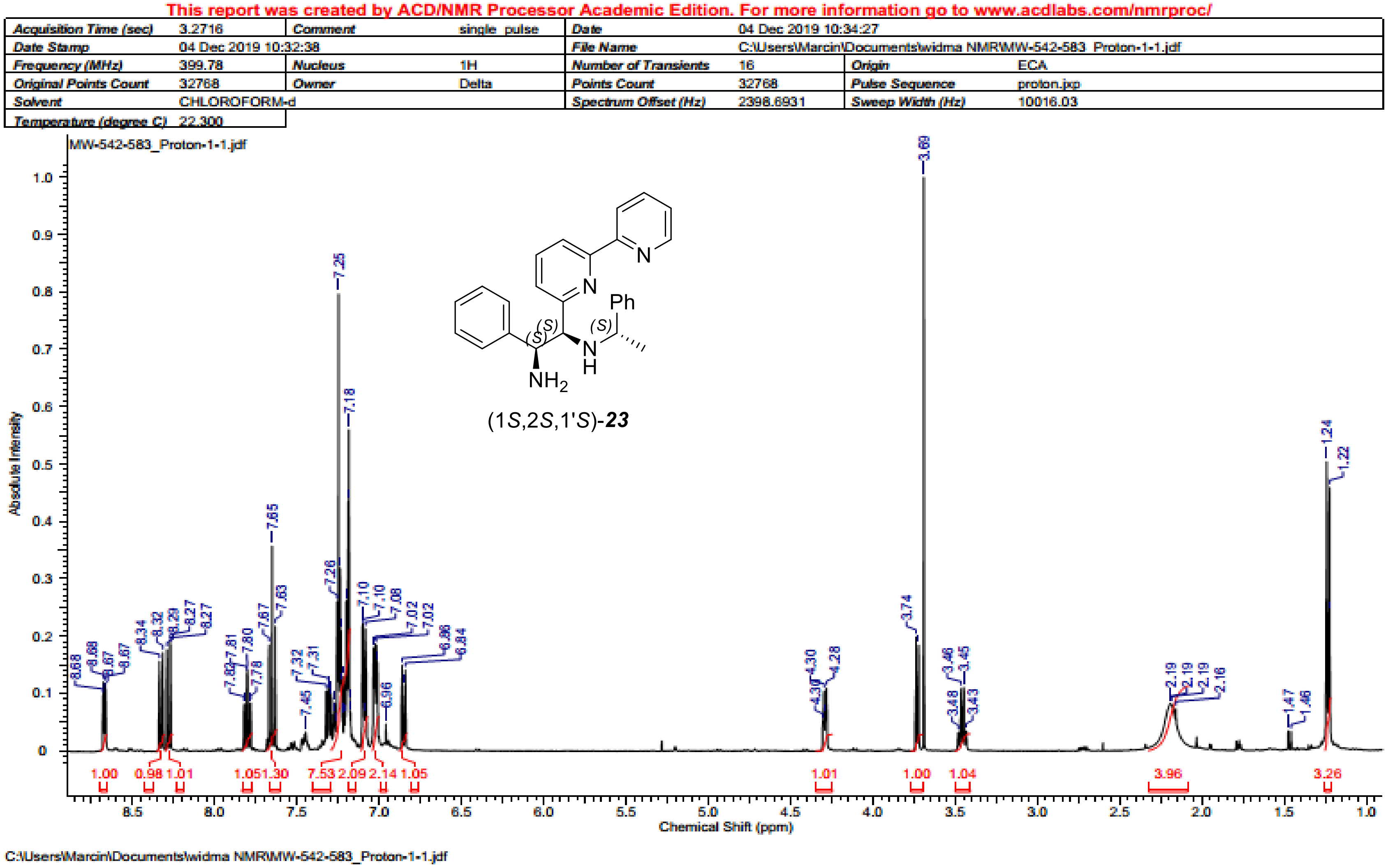

Figure S56. ^{1}H NMR spectrum (400 MHz, $CDCl_3$) for (1*S*,2*S*,1'*S*)-**23**

This report was created by ACD/NMR Processor Academic Edition. For more information go to www.acdlabs.com/nmrproc/

Acquisition Time (sec)	1.7406	*Comment*	single pulse decoupled gated NOE	*Date*	05 Dec 2019 10:22:40
Date Stamp	05 Dec 2019 09:35:23	*File Name*	C:\Users\Marcin\Documents\widma NMR\MW-542-583_carbon-1-1.jdf		
Frequency (MHz)	100.53	*Nucleus*	13C	*Number of Transients*	992
Original Points Count	65536	*Owner*	Delta	*Points Count*	65536
Origin	ECA	*Pulse Sequence*	carbon.jxp	*Solvent*	CHLOROFORM-d
Spectrum Offset (Hz)	10052.5303	*Sweep Width (Hz)*	37650.60	*Temperature (degree C)*	22.500

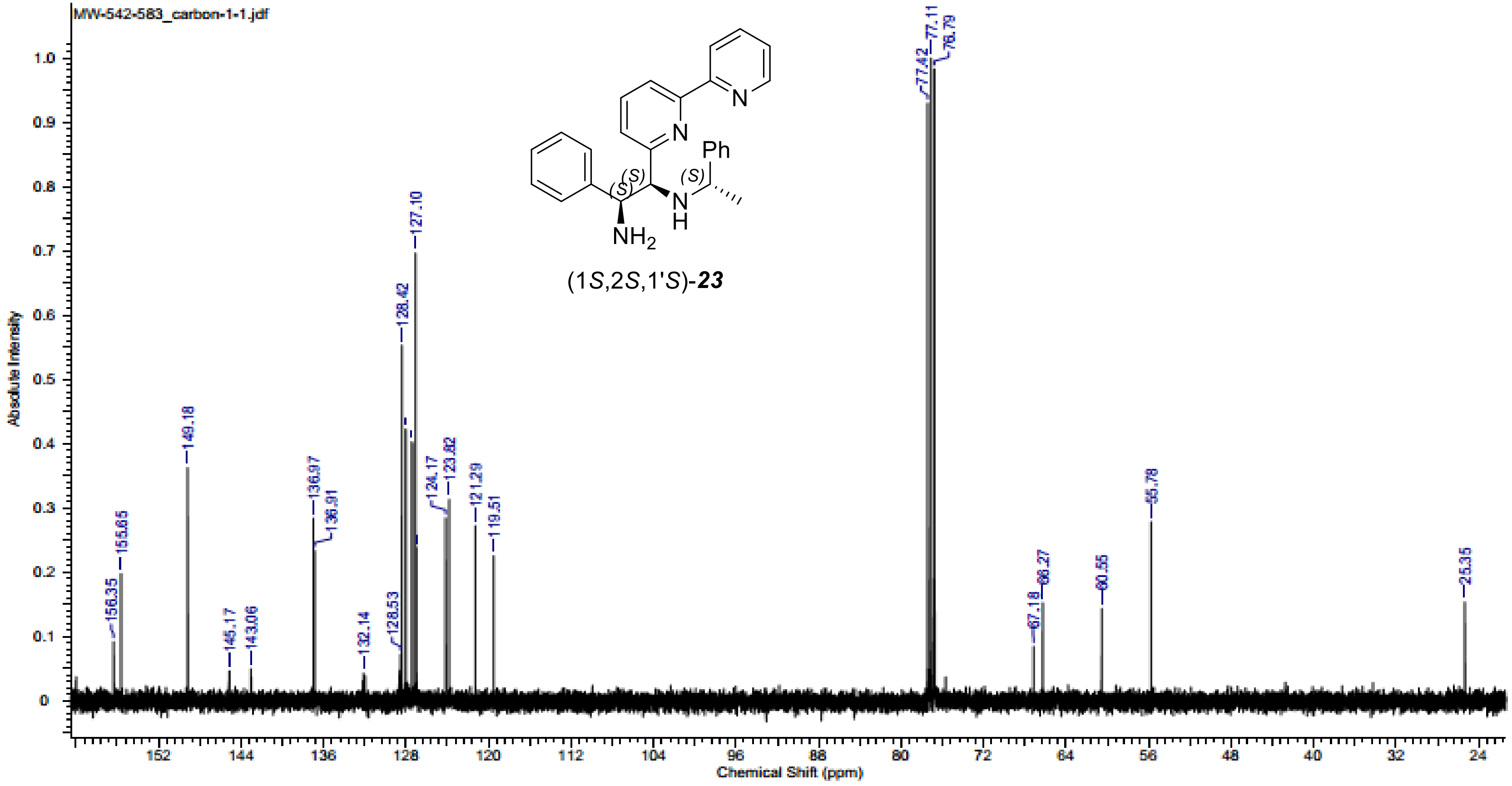

C:\Users\Marcin\Documents\widma NMR\MW-542-583_carbon-1-1.jdf

Figure S57. ^{13}C NMR spectrum (101 MHz, $CDCl_3$) for (1*S*,2*S*,1’*S*)-**23**

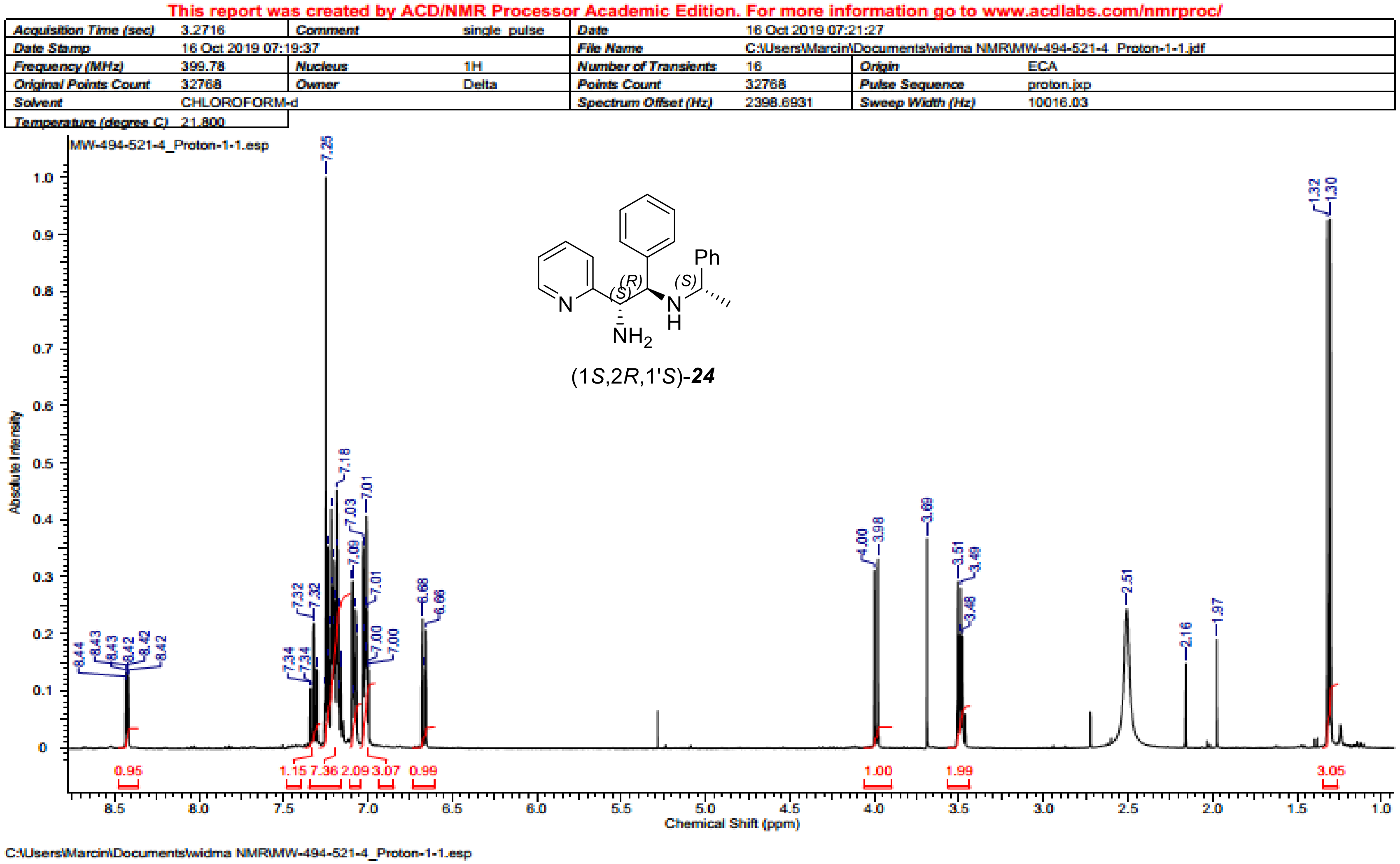

Figure S58. ^{1}H NMR spectrum (400 MHz, $CDCl_3$) for (1*S*,2*R*,1'*S*)-**24**

This report was created by ACD/NMR Processor Academic Edition. For more information go to www.acdlabs.com/nmrproc/

Acquisition Time (sec)	0.9088	*Comment*	5 mm PABBO BB-1H/D Z-GRD Z847801/0325			*Date*	24 Oct 2019 14:35:12
Date Stamp	24 Oct 2019 14:35:12			*File Name*	C:\Users\Marcin\Documents\widma NMR\MW-494-521-4\1\fid		
Frequency (MHz)	151.02	*Nucleus*	13C	*Number of Transients*	5941	*Origin*	spect
Original Points Count	32768	*Owner*	nmrsu	*Points Count*	32768	*Pulse Sequence*	zgpg30
Receiver Gain	2050.00	*SW(cyclical) (Hz)*	36057.69	*Solvent*	CHLOROFORM-d		
Spectrum Offset (Hz)	15100.6848	*Spectrum Type*	STANDARD	*Sweep Width (Hz)*	36056.59	*Temperature (degree C)*	25.000

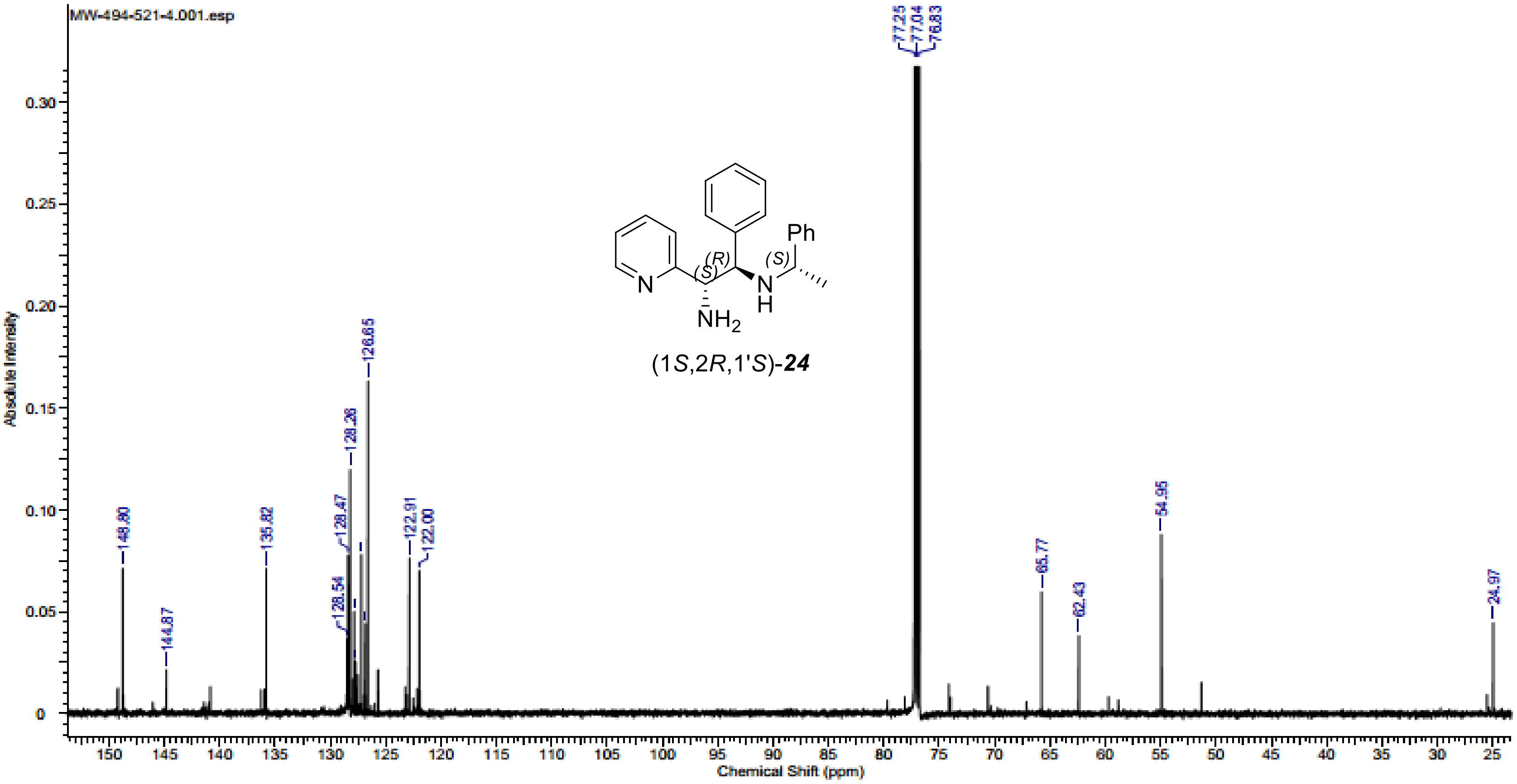

C:\Users\Marcin\Documents\widma NMR\MW-494-521-4\MW-494-521-4.001.esp

Figure S59. ^{13}C NMR spectrum (151 MHz, $CDCl_3$) for (1*S*,2*R*,1'*S*)-**24**

This report was created by ACD/NMR Processor Academic Edition. For more information go to www.acdlabs.com/nmrproc/

Acquisition Time (sec)	2.7263	*Comment*	5 mm PABBO BB-1H/D Z-GRD Z847801/0325			*Date*	29 Jan 2020 12:52:32
Date Stamp	29 Jan 2020 12:52:32			*File Name*	C:\Users\Marcin\Documents\widma NMR\MW-561-612\1\fid		
Frequency (MHz)	600.58	*Nucleus*	1H	*Number of Transients*	16	*Origin*	spect
Original Points Count	32768	*Owner*	nmrsu	*Points Count*	32768	*Pulse Sequence*	zg30
Receiver Gain	114.00	*SW(cyclical) (Hz)*	12019.23	*Solvent*	CHLOROFORM-d		
Spectrum Offset (Hz)	3708.5806	*Spectrum Type*	STANDARD	*Sweep Width (Hz)*	12018.86	*Temperature (degree C)*	25.000

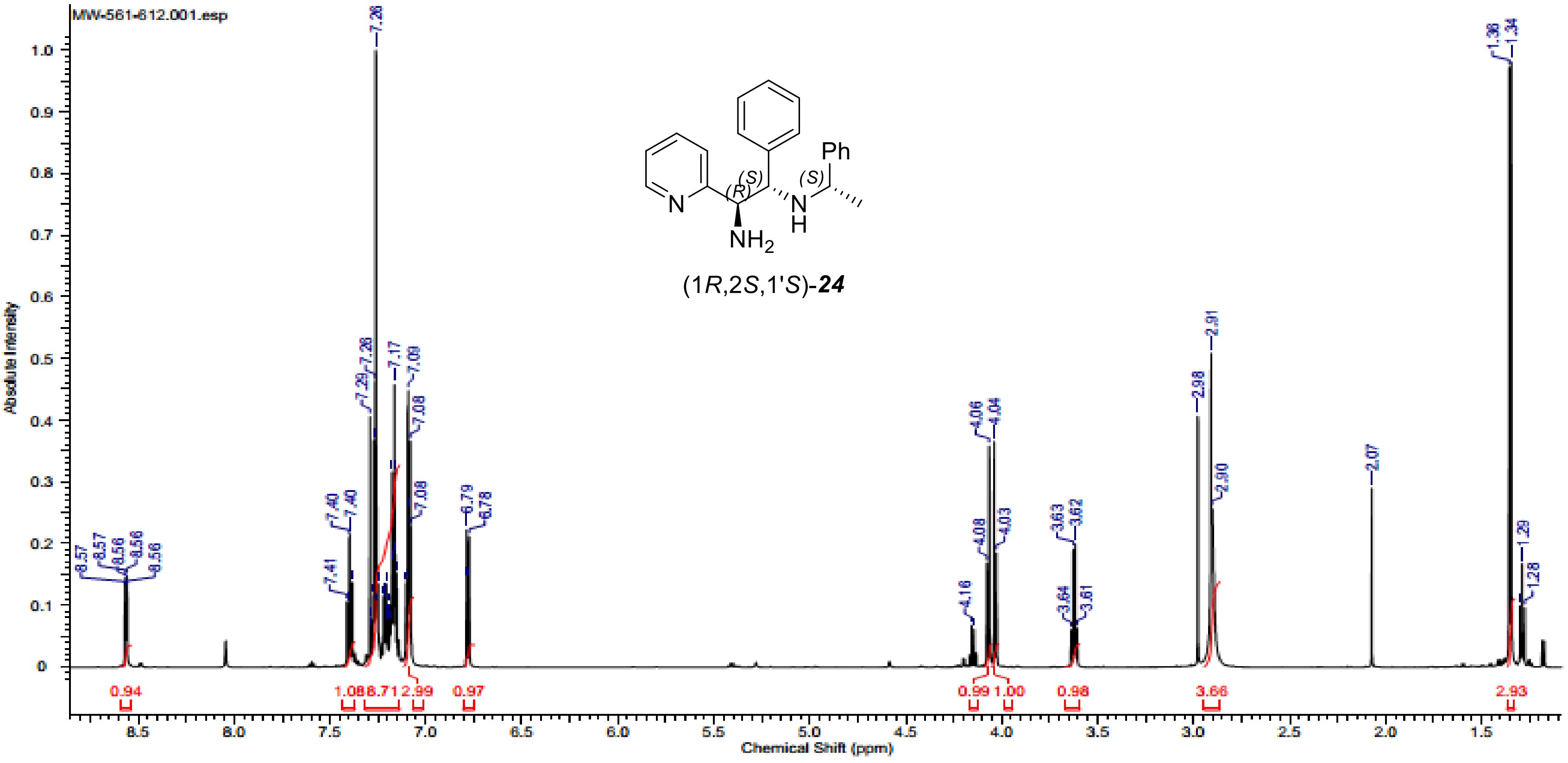

C:\Users\Marcin\Documents\widma NMR\MW-561-612\MW-561-612.001.esp

Figure S60. ^{1}H NMR spectrum (600 MHz, $CDCl_3$) for (1*R*,2*S*,1'*S*)-**24**

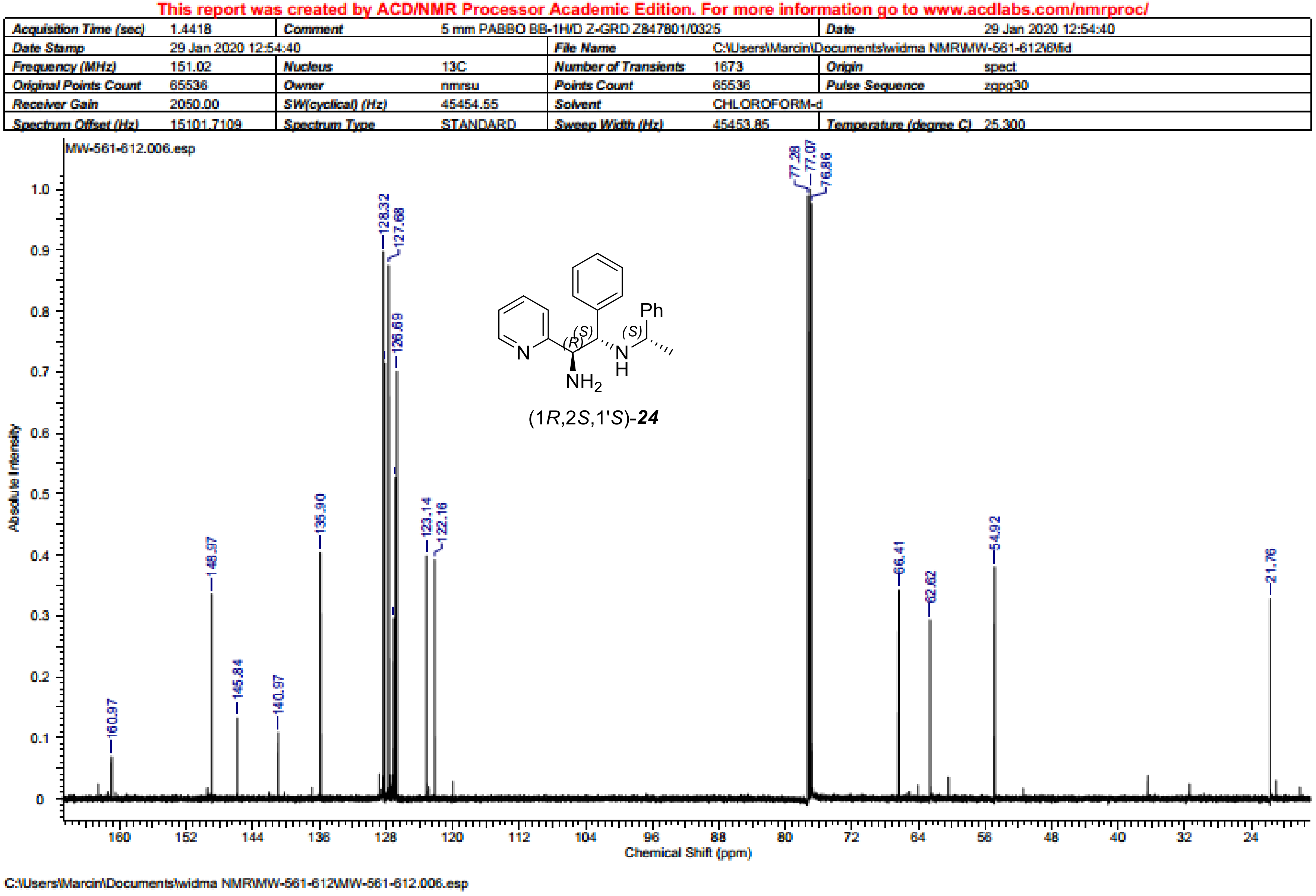

Figure S61. ^{1}H NMR spectrum (151 MHz, $CDCl_3$) for (1*R*,2*S*,1'*S*)-**24**

6. DFT/B3LYP/CC-pVDZ geometries listings

(1*S*,2*S*,3*R*)-**10**

```
C        -0.459858    0.635679   -0.599285
C        -1.474910    0.261520    0.459101
H        -0.765139    0.524180   -1.645123
H        -1.340591    0.829318    1.387878
N        -0.409632   -0.661744    0.102340
C        -2.889451   -0.087217    0.122907
C        -3.199445   -1.185723   -0.692964
C        -3.936528    0.705541    0.618290
C        -4.527296   -1.477438   -1.013788
H        -2.383997   -1.812875   -1.057391
C        -5.264502    0.414434    0.296657
H        -3.706965    1.558978    1.262452
C        -5.564769   -0.678074   -0.522859
H        -4.754683   -2.338542   -1.647204
H        -6.068358    1.040954    0.690786
H        -6.602902   -0.908449   -0.773286
C         0.503028    1.757469   -0.381726
C         1.123507    2.354039   -1.492806
C         1.588101    3.195473    1.054127
C         2.001228    3.417928   -1.293056
H         0.915874    1.981578   -2.497817
C         2.243547    3.853991    0.011151
H         1.751694    3.504839    2.092044
H         2.491092    3.898315   -2.143283
H         2.921746    4.683568    0.219155
N         0.735391    2.179342    0.876142
C         0.639240   -0.949836    1.096398
H         0.884434   -0.051160    1.688320
C         0.098368   -2.025422    2.050422
H        -0.158875   -2.942466    1.497525
H         0.854054   -2.277575    2.810285
H        -0.806091   -1.667508    2.568357
C         1.909700   -1.415039    0.398595
C         3.163130   -1.088519    0.936413
C         1.862541   -2.209059   -0.757609
C         4.341866   -1.548978    0.342645
H         3.215807   -0.461116    1.830635
C         3.039425   -2.667060   -1.356088
H         0.891478   -2.457275   -1.189138
C         4.283792   -2.341035   -0.807540
H         5.308773   -1.282220    0.776478
H         2.984611   -3.282624   -2.257633
H         5.203448   -2.699161   -1.276318
 Number of imaginary frequencies:                0
 Zero-point correction=                          0.356222
(Hartree/Particle)
 Thermal correction to Energy=                   0.375467
 Thermal correction to Enthalpy=                 0.376411
 Thermal correction to Gibbs Free Energy=        0.305772
```

Sum of electronic and zero-point Energies= -921.446668
Sum of electronic and thermal Energies= -921.427423
Sum of electronic and thermal Enthalpies= -921.426479
Sum of electronic and thermal Free Energies= -921.497118

(1*R*,2*S*,3*R*)-**10**

C	-1.506830	0.240225	0.402129
C	-0.502521	0.644604	-0.642542
H	-1.441923	0.788039	1.348958
H	-0.803044	0.507113	-1.688533
N	-0.402441	-0.652174	0.059707
C	0.446730	1.790958	-0.435840
C	0.123004	2.893226	0.370278
C	1.675715	1.795888	-1.118504
C	1.010889	3.965851	0.503848
H	-0.839663	2.922743	0.884398
C	2.563806	2.864559	-0.983705
H	1.938751	0.947809	-1.754676
C	2.234734	3.953425	-0.169441
H	0.739833	4.816774	1.133269
H	3.517087	2.847130	-1.517086
H	2.928275	4.791075	-0.065079
C	-2.907213	-0.163489	0.070950
C	-3.184636	-1.248854	-0.773297
C	-5.141701	0.288204	0.343771
C	-4.515206	-1.550894	-1.058718
H	-2.358956	-1.837527	-1.174650
C	-5.521818	-0.764631	-0.492211
H	-5.901635	0.921370	0.814622
H	-4.765055	-2.391799	-1.710491
H	-6.577586	-0.964021	-0.686042
N	-3.868943	0.592198	0.629733
C	0.634330	-0.850788	1.084822
H	0.877917	0.102288	1.594558
C	0.096276	-1.832434	2.137060
H	-0.160117	-2.795746	1.669028
H	0.854638	-2.015664	2.913554
H	-0.807127	-1.431237	2.623949
C	1.915183	-1.379922	0.451772
C	3.159731	-1.049944	1.008140
C	1.881954	-2.239648	-0.656324
C	4.343507	-1.569361	0.477475
H	3.202566	-0.370761	1.864248
C	3.065202	-2.756924	-1.192185
H	0.917706	-2.491942	-1.100150
C	4.300267	-2.425904	-0.626711
H	5.303381	-1.297796	0.923551
H	3.021687	-3.422993	-2.057664
H	5.224533	-2.829895	-1.046434

Number of imaginary frequencies: 0

Zero-point correction= (Hartree/Particle)		0.356123
Thermal correction to Energy=		0.375379
Thermal correction to Enthalpy=		0.376323
Thermal correction to Gibbs Free Energy=		0.305543
Sum of electronic and zero-point Energies=		-921.442962
Sum of electronic and thermal Energies=		-921.423707
Sum of electronic and thermal Enthalpies=		-921.422762
Sum of electronic and thermal Free Energies=		-921.493542

Complex of (1*S*,2*S*,3*R*)-**10** with zinc acetate

C	-0.267721	-1.839760	-0.762913
C	-1.125293	-1.307844	0.354854
H	-0.822767	-2.313371	-1.576978
H	-0.653593	-1.226963	1.339627
N	-0.426019	-0.397934	-0.595366
C	-2.605705	-1.569601	0.411362
C	-3.180316	-2.722383	-0.152940
C	-3.434041	-0.692313	1.133456
C	-4.549178	-2.978685	-0.025979
H	-2.553775	-3.437399	-0.691845
C	-4.802430	-0.944070	1.256384
H	-3.002731	0.200000	1.593287
C	-5.366510	-2.086456	0.674354
H	-4.975669	-3.882084	-0.472539
H	-5.430633	-0.243699	1.812918
H	-6.436748	-2.283905	0.772741
C	1.088656	-2.438749	-0.559215
C	1.316400	-3.798185	-0.810825
C	3.318296	-2.125064	0.022380
C	2.593897	-4.319796	-0.615452
H	0.497113	-4.431546	-1.156669
C	3.618059	-3.468203	-0.192218
H	4.077017	-1.397529	0.316212
H	2.791644	-5.377874	-0.801859
H	4.635450	-3.831400	-0.040704
N	2.078949	-1.630020	-0.145812
C	-1.066396	0.453524	-1.642891
H	-0.193712	0.777266	-2.243287
C	-2.055269	-0.258261	-2.590702
H	-2.976404	-0.583018	-2.078475
H	-2.342380	0.424505	-3.402389
H	-1.588924	-1.130512	-3.082251
C	-1.646526	1.733510	-1.018864
C	-2.354673	2.661489	-1.836184
C	-1.439640	2.065012	0.332184
C	-2.860670	3.860187	-1.318957
H	-2.507851	2.463416	-2.902348
C	-1.948428	3.265825	0.851370
H	-0.859824	1.404385	0.994647
C	-2.666026	4.166720	0.034066
H	-3.406101	4.559693	-1.981722

H	-1.767663	3.497658	1.906423
H	-3.059239	5.104485	0.441505
Zn	1.647065	0.434378	0.263075
C	3.085792	1.694184	-1.328818
O	3.723719	0.829920	-0.679861
C	1.633926	1.007650	2.799454
O	2.629481	1.547515	2.295205
O	1.810215	1.821630	-1.186377
O	0.809382	0.305164	2.076147
C	3.788574	2.639787	-2.276118
H	3.874004	3.625670	-1.786479
H	4.795480	2.271239	-2.515228
H	3.194818	2.779915	-3.193114
C	1.309127	1.124248	4.276033
H	0.301954	1.553903	4.403546
H	1.290851	0.120024	4.731236
H	2.051444	1.751053	4.785906

(1*S*,2*R*,3*S*)-**13**

C	0.026440	0.883443	0.263839
C	-1.207018	0.862766	-0.601543
H	-0.091246	0.495076	1.280180
H	-1.259755	1.630925	-1.381263
N	-0.073645	-0.063679	-0.845273
C	-2.564758	0.453215	-0.113435
C	-2.835393	0.005145	1.191575
C	-3.639131	0.547094	-1.018282
C	-4.133309	-0.353297	1.570458
H	-2.035119	-0.051792	1.930959
C	-4.936159	0.192244	-0.640553
H	-3.450962	0.906684	-2.033550
C	-5.188178	-0.263691	0.657596
H	-4.320007	-0.695576	2.591259
H	-5.752901	0.275342	-1.361805
H	-6.201647	-0.540045	0.957899
C	1.028366	1.990472	0.188173
C	1.592405	2.401064	-1.029812
C	2.210831	3.588721	1.346762
C	2.500542	3.458419	-1.027791
H	1.323549	1.883446	-1.951646
C	2.819081	4.072504	0.186950
H	2.440307	4.038535	2.318778
H	2.957424	3.797521	-1.960801
H	3.525654	4.903277	0.237858
N	1.335942	2.571949	1.362162
C	-0.214094	-1.495595	-0.513693
H	-0.887813	-1.645323	0.349388
C	-0.820218	-2.217491	-1.724187
H	-0.177231	-2.098923	-2.611183
H	-0.929550	-3.293618	-1.516396
H	-1.816195	-1.811571	-1.962264
C	1.141719	-2.078547	-0.135139

```
C         1.257377   -2.924081    0.978248
C         2.289584   -1.821167   -0.903156
C         2.484833   -3.504643    1.316704
H         0.373868   -3.131512    1.588730
C         3.518098   -2.394802   -0.564466
H         2.219214   -1.158316   -1.767568
C         3.620595   -3.240758    0.545942
H         2.553053   -4.159970    2.188749
H         4.401839   -2.179519   -1.170679
H         4.581756   -3.688631    0.810005
 Number of imaginary frequencies:                 0
 Zero-point correction=                           0.356095
(Hartree/Particle)
 Thermal correction to Energy=                    0.375321
 Thermal correction to Enthalpy=                  0.376265
 Thermal correction to Gibbs Free Energy=         0.305986
 Sum of electronic and zero-point Energies=           -921.468019
 Sum of electronic and thermal Energies=              -921.448793
 Sum of electronic and thermal Enthalpies=            -921.447848
 Sum of electronic and thermal Free Energies=         -921.518128
```

(1*R*,2*R*,3*S*)-**10**

```
C        -1.260653    0.768308   -0.672420
C        -0.070229    0.865462    0.254287
H        -1.330145    1.513919   -1.471413
H        -0.272170    0.477373    1.258827
N        -0.078595   -0.106621   -0.833154
C         0.885713    2.013782    0.204515
C         1.493434    2.417037   -0.995933
C         1.173989    2.727029    1.379221
C         2.363102    3.510173   -1.020155
H         1.287945    1.855670   -1.909595
C         2.043725    3.821557    1.355586
H         0.713276    2.418052    2.321613
C         2.640953    4.218471    0.154833
H         2.830842    3.809098   -1.961805
H         2.258620    4.363448    2.280110
H         3.322755    5.072142    0.134897
C        -2.600317    0.326047   -0.175946
C        -3.747036    0.667638   -0.914028
C        -3.886267   -0.742279    1.412789
C        -5.001319    0.282491   -0.441803
H        -3.648856    1.230975   -1.844166
C        -5.078362   -0.440963    0.750339
H        -3.903826   -1.308933    2.349788
H        -5.905340    0.543006   -0.997369
H        -6.036475   -0.764058    1.161623
N        -2.675502   -0.370174    0.975628
C        -0.156343   -1.544259   -0.496255
H        -0.812301   -1.712542    0.373719
C        -0.740909   -2.295115   -1.699016
H        -0.119406   -2.146051   -2.596839
```

```
H        -0.794306   -3.375267   -1.489937
H        -1.759938   -1.941020   -1.924393
C         1.232089   -2.056064   -0.133692
C         1.411919   -2.847619    1.010331
C         2.348956   -1.782704   -0.941114
C         2.671379   -3.361532    1.339698
H         0.553455   -3.064338    1.652266
C         3.608809   -2.290135   -0.612654
H         2.229946   -1.158427   -1.828695
C         3.775168   -3.083476    0.528640
H         2.789608   -3.975656    2.236108
H         4.467209   -2.063589   -1.250401
H         4.760979   -3.479332    0.784997
 Number of imaginary frequencies:                 0
 Zero-point correction=                           0.355913
(Hartree/Particle)
 Thermal correction to Energy=                    0.375183
 Thermal correction to Enthalpy=                  0.376127
 Thermal correction to Gibbs Free Energy=         0.305374
 Sum of electronic and zero-point Energies=           -921.471695
 Sum of electronic and thermal Energies=              -921.452426
 Sum of electronic and thermal Enthalpies=            -921.451482
 Sum of electronic and thermal Free Energies=         -921.522235
```

(1*S*,2*S*,3*R*)-**12**

```
C        -1.238589   -0.051912    1.038778
C        -1.774221   -0.871897   -0.111469
H        -1.918288    0.138080    1.875518
H        -0.993817   -1.430504   -0.640170
N        -1.715106    0.580719   -0.210419
C        -3.106027   -1.551890   -0.062912
C        -4.293712   -0.834840    0.153245
C        -3.177405   -2.945708   -0.221529
C        -5.522053   -1.498300    0.217602
H        -4.244377    0.250696    0.256701
C        -4.405776   -3.609736   -0.156133
H        -2.260205   -3.514426   -0.398668
C        -5.583540   -2.888127    0.065557
H        -6.438393   -0.925867    0.383291
H        -4.443247   -4.694676   -0.282697
H        -6.544714   -3.405462    0.114902
C         0.194373   -0.130258    1.463921
C         0.548227    0.365886    2.730282
C         2.385143   -0.753485    1.028243
C         1.878943    0.284860    3.137364
H        -0.212668    0.807303    3.376419
C         2.818034   -0.280648    2.277370
H         2.182014    0.660625    4.117370
H         3.869270   -0.368107    2.546663
N         1.099228   -0.680550    0.636437
C        -0.767941    1.181447   -1.168378
```

H	0.182346	0.621835	-1.195934
C	-1.401344	1.119342	-2.565491
H	-2.359030	1.664052	-2.588588
H	-0.728105	1.570287	-3.311406
H	-1.590656	0.075064	-2.863256
C	-0.452468	2.616820	-0.766114
C	0.845645	3.122314	-0.934894
C	-1.446154	3.474957	-0.267189
C	1.145674	4.452366	-0.622221
H	1.633064	2.464087	-1.312731
C	-1.148359	4.803573	0.050029
H	-2.457638	3.091940	-0.120025
C	0.148327	5.298711	-0.128057
H	2.163863	4.825526	-0.758979
H	-1.933783	5.456560	0.439677
H	0.380509	6.337157	0.120867
C	3.354351	-1.375924	0.075864
C	2.929249	-1.935407	-1.140716
N	4.649174	-1.374817	0.449632
C	3.877417	-2.503484	-1.990618
H	1.871900	-1.922295	-1.399757
C	5.547769	-1.922909	-0.375367
C	5.219841	-2.499179	-1.605951
H	3.570760	-2.945606	-2.941494
H	6.588679	-1.901193	-0.035814
H	5.997024	-2.931968	-2.238720

Number of imaginary frequencies: 0
Zero-point correction= 0.424698 (Hartree/Particle)
Thermal correction to Energy= 0.448577
Thermal correction to Enthalpy= 0.449521
Thermal correction to Gibbs Free Energy= 0.367040
Sum of electronic and zero-point Energies= -1168.525306
Sum of electronic and thermal Energies= -1168.501427
Sum of electronic and thermal Enthalpies= -1168.500483
Sum of electronic and thermal Free Energies= -1168.582964

(1*R*,2*S*,3*R*)-**12**

C	-0.059946	-0.148413	0.109148
C	0.993630	0.347871	-0.848065
H	-0.228058	0.473060	0.994735
H	0.864915	0.061185	-1.897557
N	1.217306	-0.846266	0.001694
C	1.714069	1.654241	-0.694347
C	1.491125	2.561981	0.355812
C	2.657936	1.998120	-1.679851
C	2.201998	3.764722	0.425120
H	0.746730	2.344273	1.123275
C	3.366321	3.199716	-1.613648
H	2.838271	1.308646	-2.508727

C	3.142823	4.088392	-0.556786
H	2.010014	4.456513	1.248967
H	4.094349	3.443096	-2.391326
H	3.693712	5.030372	-0.502730
C	-1.305931	-0.833290	-0.357073
C	-1.265554	-1.995770	-1.141819
C	-3.620960	-0.800447	-0.383159
C	-2.472334	-2.559960	-1.553344
H	-0.304624	-2.439888	-1.402342
C	-3.672210	-1.957830	-1.175329
H	-2.479264	-3.466901	-2.162810
H	-4.638821	-2.362050	-1.471724
N	-2.457016	-0.254937	0.017676
C	2.126295	-0.781864	1.159315
H	2.121996	0.222353	1.622049
C	1.639277	-1.787956	2.212097
H	1.624184	-2.809737	1.799827
H	2.305817	-1.778390	3.088680
H	0.621728	-1.537463	2.553893
C	3.556185	-1.085405	0.727589
C	4.629430	-0.424463	1.344369
C	3.837462	-2.055264	-0.247963
C	5.951463	-0.727544	1.003428
H	4.426937	0.341702	2.098094
C	5.158221	-2.357230	-0.594023
H	3.011578	-2.568779	-0.743274
C	6.220922	-1.696437	0.031609
H	6.772977	-0.199285	1.493983
H	5.358232	-3.113050	-1.358068
H	7.252866	-1.932091	-0.239941
C	-4.873102	-0.116066	0.058005
C	-4.824373	1.056518	0.830471
N	-6.037586	-0.676911	-0.323206
C	-6.020083	1.660277	1.216149
H	-3.856973	1.470642	1.110989
C	-7.176976	-0.086633	0.054437
C	-7.228239	1.080098	0.822550
H	-6.008831	2.573175	1.816389
H	-8.102146	-0.571916	-0.274252
H	-8.189229	1.517138	1.100568

Number of imaginary frequencies: 0
Zero-point correction= 0.424650
(Hartree/Particle)
Thermal correction to Energy= 0.448595
Thermal correction to Enthalpy= 0.449539
Thermal correction to Gibbs Free Energy= 0.365924
Sum of electronic and zero-point Energies= -1168.522832
Sum of electronic and thermal Energies= -1168.498888
Sum of electronic and thermal Enthalpies= -1168.497943
Sum of electronic and thermal Free Energies= -1168.581558
